# Genome Divergence and Dynamics in the Thin-Tailed Desert Sheep From Sudan

**DOI:** 10.3389/fgene.2021.659507

**Published:** 2021-07-19

**Authors:** Adam Abied, Abulgasim M. Ahbara, Haile Berihulay, Lingyang Xu, Rabiul Islam, Faisal M. El-Hag, Mourad Rekik, Aynalem Haile, Jian-Lin Han, Yuehui Ma, Qianjun Zhao, Joram M. Mwacharo

**Affiliations:** ^1^Institute of Animal Science, Chinese Academy of Agricultural Sciences, Beijing, China; ^2^Dry Land Research Centre and Animal Production, Agricultural Research Corporation, Khartoum, Sudan; ^3^Small Ruminant Genomics, International Center for Agricultural Research in the Dry Areas (ICARDA), Addis Ababa, Ethiopia; ^4^Arid Land Research Centre, Tottori University, Tottori, Japan; ^5^International Center for Agricultural Research in the Dry Areas (ICARDA), Amman, Jordan; ^6^CAAS-ILRI Joint Laboratory on Livestock and Forage Genetic Resources, Institute of Animal Science, Chinese Academy of Agricultural Sciences, Beijing, China; ^7^Livestock Genetics Program, International Livestock Research Institute, Nairobi, Kenya; ^8^Animal and Veterinary Sciences, Scotland Rural College and Centre for Tropical Livestock Genetics and Health (CTLGH), Roslin Institute, Midlothian, United Kingdom

**Keywords:** adaptation, climate change, genetic diversity, selection signatures, SNP genotypes

## Abstract

With climate change bound to affect food and feed production, emphasis will shift to resilient and adapted indigenous livestock to sustain animal production. However, indigenous livestock comprise several varieties, strains and ecotypes whose genomes are poorly characterized. Here, we investigated genomic variation in an African thin-tailed Desert Sheep sampled in Sudan, using 600K genotype data generated from 92 individuals representing five ecotypes. We included data from 18 fat-tailed and 45 thin-tailed sheep from China, to investigate shared ancestry and perform comparative genomic analysis. We observed a clear genomic differentiation between the African thin-tailed Desert Sheep and the Chinese thin-tailed and fat-tailed sheep, suggesting a broad genetic structure between the fat-tailed and thin-tailed sheep in general, and that at least two autosomal gene pools comprise the genome profile of the thin-tailed sheep. Further analysis detected two distinct genetic clusters in both the African thin-tailed Desert Sheep and the Chinese thin-tailed sheep, suggesting a fine-scale and complex genome architecture in thin-tailed sheep. Selection signature analysis suggested differences in adaptation, production, reproduction and morphology likely underly the fine-scale genetic structure in the African thin-tailed Desert Sheep. This may need to be considered in designing breeding programs and genome-wide association studies.

## Introduction

A common research thread that links population and quantitative genomics is the elucidation of patterns and processes underlying population genetic structure. Whether such structure is stable in time and space is increasingly addressed for its utility in determining how many genetically distinct populations exist and their inter-relationships (Nielsen et al., 1999; Waples and Gaggiotti, 2006). Insights from such investigations inform management decisions that define conservation units and the design of genetic monitoring and breeding programs (Palsbøll et al., 2007; Schwartz et al., 2007). The tropics and sub-tropics are home to a large reservoir of indigenous livestock with a high degree of adaptive resilience, and which support agricultural and non-agricultural industries with minimal anthropogenic interventions (FAO, 2007). Indigenous livestock can therefore provide a foundation to sustain production under increasing challenges resulting from global warming and rising human demand for livestock products. It is also likely that future livestock production will come from marginal areas where arable agriculture is at high risk of failure and thus particular attention would have to be given to the uniqueness of genetic features, as it is difficult to predict the future importance of traits and alleles (Taberlet et al., 2008).

Domestic sheep (*Ovis aries*) are central to national economies as a source of cash, meat, milk, fiber, etc., and to traditional societies as repositories of socio-cultural values. Sheep are also essential components of diverse production systems due partly to their versatility to adapt to local biophysical and production environments. Domestic sheep comprise three broad types: thin-tailed, fat-tailed, and fat-rumped sheep (Porter, 2020). Thin-tailed sheep are the most ancient and in the African continent, two types are recognized: the long-legged (Sahelian) and the tropical Dwarf (Djallonké) sheep. The Sahelian is confined to the hot arid marginal environments in eastern, western and northern Africa, while the Djallonke is well adapted to sub-humid and humid tropics of western and central Africa. The analysis of mitochondrial genomes has shown that the Sahelian and Djallonke comprise separate maternal ancestries (Brahi et al., 2015).

The long-legged thin-tailed sheep found in Sudan represents the complexity that is typical of most indigenous livestock. They are subdivided into Desert, Nilotic, Arid upland, Arid equatorial, and West African populations, including their inter-crosses, following their eco-geographic distribution (Abualazayium, 2004). Within the Sudanese thin-tailed Desert Sheep, a long-legged Sahelian thin-tailed sheep, at least eight ecotypes, Hammari, Kabashi, Shanbali, Shugor, Dubasi, Watish, Al Ahamda, and Borouge, have been described (Abualazayium, 2004). Whether these ecotypes represent real underlying genetic variation remains unknown. If confirmed, they could offer a powerful genetic model to investigate drivers of divergence in indigenous livestock. Understanding fine-scale genetic structure is also important to control confounding effects of population stratification in association studies (Price et al., 2010). In this study, we applied distance- and model-based comparative genomic approaches to 600K single nucleotide polymorphism (SNP) genotype data from 121 individuals of five ecotypes of the Sudanese thin-tailed Desert Sheep, to investigate their genome architecture and dynamics. We analyzed the dataset alongside similar data from one fat-tailed and four thin-tailed breeds of sheep from China, to investigate their shared genome ancestry and for comparative genomic assessment.

## Materials and Methods

### Sample Collection and DNA Extraction

Whole blood was collected through jugular venipuncture from 121 animals representing five ecotypes of thin-tailed Desert Sheep in Sudan (Supplementary Tables 1, 2). Blood samples were also collected from 65 individuals of four breeds of Chinese sheep, including one fat-tailed breed (Tan Sheep) from a dry lowland environment in Ningxia Province and three thin-tailed breeds (Oula, Zeku, and Black Tibetan) from the alpine high-altitude in Qinghai Province (Supplementary Table 1); these were used for comparative genomic analysis. The DNeasy^®^ Blood and Tissue Kit (Qiagen Inc., United States) and the phenol-chloroform method were used to extract DNA from the Sudanese and Chinese samples, respectively. The samples were genotyped with the Ovine Infinium 600K BeadChip at GeneSeek Inc. (Sudanese samples) and Compass Biotechnology (Chinese samples). Of the 606,006 SNPs present in the BeadChip, 577,401 are autosomal, 27,314 are on the X-chromosome and 1,291 remain unmapped.

### Data Screening and Quality Control

The genotypes from both sets of samples were merged and the data were screened for quality with PLINK (Chang et al., 2015). Samples with more than 10% missing genotypes, SNPs with less than 90% genotype call rates, Hardy-Weinberg equilibrium (HWE) threshold of 1e-10 and minor allele frequency (MAF) < 0.01 were discarded. Using the “genome – min 0.05 – max 1” flag, the Pi-HAT statistic was calculated to assess the level of genetic relatedness between individuals and determine outliers with the objective of excluding the outliers and at least one amongst a pair of individuals that showed close genetic relationship. The value of 0.1875 which represents the half-way point between the 2nd and 3rd degree relatives was used as the cut-off threshold. These filtering thresholds left 155 samples (Table 1) and 519,711 SNPs which were used for selection signature analyses. Using PLINK, the 519,711 SNPs were subjected to linkage disequilibrium (LD) pruning using the parameters 50, 5, and 0.5 representing window size (kb), step size (kb), and *r*^2^ threshold, respectively, resulting in 390,243 SNPs that were used for population structure and phylogenetic analysis.

**TABLE 1:** Genetic diversity estimates in the five ecotypes of Sudanese thin-tailed Desert Sheep and the four breeds of sheep from China analyzed in this study.

Ecotype/Breed	Abb.	*N*	H_*O*_ (mean ± SD)	H_*E*_ (mean ± SD)	Pi-HAT	ROH (Mb) (mean ± SD)	*F*_*ROH*_ (mean ± SD)	*F* (mean ± SD)	Origin
Al-Ahamda	AL	19	0.345 ± 0.010	0.347 ± 0.0001	0.092 ± 0.009	1.886 ± 0.634	0.014 ± 0.026	0.006 ± 0.031	Sudan
Buzee	BU	18	0.347 ± 0.008	0.348 ± 0.0001	0.096 ± 0.013	1.735 ± 0.324	0.015 ± 0.013	0.002 ± 0.023	
Hammari	HA	23	0.341 ± 0.004	0.343 ± 0.0000	0.081 ± 0.005	1.841 ± 0.526	0.013 ± 0.009	0.005 ± 0.011	
Kabashi	KA	17	0.353 ± 0.010	0.357 ± 0.0001	0.112 ± 0.039	1.748 ± 0.718	0.019 ± 0.012	0.011 ± 0.029	
Shanbali	SH	15	0.355 ± 0.008	0.353 ± 0.0001	0.110 ± 0.025	1.504 ± 0.227	0.009 ± 0.010	−0.005 ± 0.024	
Thin-tail Desert	**SDN**	**92**	**0.329 ± 0.008**	**0.332 ± 0.0001**	**0.056 ± 0.016**	**1.852 ± 0.335**	**0.014 ± 0.015**	**0.009 ± 0.023**	
Black Tibetan	HZ	15	0.336 ± 0.045	0.332 ± 0.0001	0.253 ± 0.068	1.208 ± 0.125	0.005 ± 0.006	−0.011 ± 0.135	China
Oula	QOL	15	0.346 ± 0.020	0.353 ± 0.0001	0.096 ± 0.031	1.169 ± 0.108	0.001 ± 0.001	0.018 ± 0.020	
Zeku	ZK	15	0.348 ± 0.005	0.352 ± 0.0002	0.103 ± 0.049	1.247 ± 0.124	0.001 ± 0.001	0.011 ± 0.016	
Tan	TS	18	0.346 ± 0.019	0.360 ± 0.0000	0.083 ± 0.034	1.351 ± 0.203	0.009 ± 0.011	0.039 ± 0.053	
Chinese sheep	**CHN**	**63**	**0.329 ± 0.008**	**0.346 ± 0.0001**	**0.097 ± 0.036**	**1.319 ± 0.176**	**0.004 ± 0.007**	**0.089 ± 0.072**	

*N: sample size; H_E_: expected heterozygosity; H_O_: observed heterozygosity; Pi-HAT: average relatedness; F: inbreeding coefficient; SD: standard deviation; Values in bold represent the average values for each statistic in across each sheep group from Sudan and China.*

### Genetic Diversity, Relationship, and Structure

The “het” and “ibc” options in PLINK were used to calculate the observed (H_*O*_) and expected (H_*E*_) heterozygosity, inbreeding coefficient *F* and Pi-HAT statistics. The “detectRUNS” package (Biscarini et al., 2019) was used to scan the genomes for runs of homozygosity (ROH) using the consecutive method (Marras et al., 2015). The parameters used to detect ROH were: (i) minimum number of SNPs in a sliding window = 50, (ii) minimum ROH length = 1 Mb, (iii) minimum number of consecutive SNPs for each ROH = 50, (iv) number of heterozygous SNPs per window = 1, (v) missing SNP calls per window = 5, (vi) minimum SNP density = 1 SNP/100 kb, and (vii) maximum gap between consecutive SNPs = 1 Mb. The ROH coefficient depicting genome-wide inbreeding (*F*_*ROH*_) was computed as the ratio of the total length of ROH to the length of autosomes (2.45 Gb) (McQuillan et al., 2008).

To explore demographic dynamics in the African thin-tailed Desert Sheep and in the thin-tailed and fat-tailed sheep from China, the trends in LD over genomic distances were examined by calculating the correlation coefficient (*r*^2^) between alleles at two SNP loci using the “indep” option in PLINK. Following Sved (1971), the effective population size (N_*E*_) was estimated with the equation *N*_*Et*_ = (1/4*c*) (1/*r*^2^ - 1), where *N*_*Et*_ represents the effective population size *t* generations ago, *t* = 1/2c, *r*^2^ is the LD between pairwise SNPs and *c* is the genetic distance in Morgan between a pair of SNPs (Tortereau et al., 2012).

Weir and Cockerham (1984) *F*_*ST*_ statistic was calculated to determine the extent of genetic differentiation using Arlequin v.3.5.2 (Excoffier and Lischer, 2010). The significance of the pairwise *F*_*ST*_ values was determined following 1,000 permutation replications of the dataset.

To investigate genetic structure, we performed neighbour-joining (NJ) phylogeny, principal component analysis (PCA) and ADMIXTURE modeling. The NJ tree was generated to visualize relationships using pairwise *F*_*ST*_ genetic distances. MEGA7 (Kumar et al., 2016) was used to construct the NJ tree with the five ecotypes of thin-tailed Desert Sheep and the four sheep breeds from China anchoring at the nodes. PCA was performed using the “pca” option in PLINK and the first two PCs were visualized using GENESIS (Buchmann and Hazelhurst, 2014). Genomic ancestry and admixture were investigated using the unsupervised clustering algorithm implemented in the ADMIXTURE toolkit v1.3 (Alexander et al., 2009). The patterns of population structure were explored by varying the number of assumed ancestral clusters between 2 ≤ *K* ≤ 8. Five iterations were performed for each *K*, summarized using CLUMPP (Jakobsson and Rosenberg, 2007) and visualized with GENESIS.

### Genome-Wide Scans for Signatures of Divergent Selection

The NJ tree, PCA and ADMIXTURE revealed evidence of broad- and fine-scale genetic structures in the datasets. To detect genomic regions showing divergence between the observed genetic structures, we analyzed the dataset for signatures of divergent selection.

Using the detectRUNS package, we implemented the frequency-based threshold approach to define genome-wide ROH islands in each genetic cluster that was revealed by the NJ tree, PCA and ADMIXTURE. This approach set a percentage of animals within a genetic cluster or a population having a SNP in an ROH region as the threshold. The threshold used in our analysis to select the genomic regions with a high frequency of ROH islands was 50%. Private ROH islands were determined by filtering out homozygous variants in ROHs in a specific genetic cluster, but which could not be found in ROHs of the other genetic clusters. This allowed the detection of either whole or segments of ROHs that were either shared or were private to a genetic cluster. The frequency of ROHs were plotted against their physical genomic positions and visualized with a Manhattan plot.

Signatures of selection were investigated using *F*_*ST*_ (Weir and Cockerham, 1984). This approach analyses allele frequency differentiation between populations and detects almost complete or long-term selection signatures. The *F*_*ST*_ algorithm was implemented with HIERFSTAT (Goudet, 2005) using a window size of 200 kb and a sliding step size of 60 kb. The window and slide-step sizes were inferred from LD decay patterns. The pairwise *F*_*ST*_ values for each SNP in each window between the genetic clusters tested were estimated as:

$$F_{\mathrm{ST}}=1-\frac{p1q1+p2q2}{2prqr}$$

where *p*1, *p*2 and *q*1, *q*2 are the frequencies of alleles “A” and “a” in the first and second genetic clusters, respectively, and *pr* and *qr* are the frequencies of alleles “A” and “a,” respectively, across the tested genetic clusters (Zhi et al., 2018). The *F*_*ST*_ values were standardized into *Z*-scores as follows:

$${\text{ZF}}_{\mathrm{ST}}\frac{{\text{F}}_{\mathrm{ST}}-\mu {\text{F}}_{\mathrm{ST}}}{\sigma {\text{F}}_{\mathrm{ST}}}$$

where μ*F*_*ST*_ is the overall average value of *F*_*ST*_ and σ*F*_*ST*_ is the standard deviation (SD) derived from all the windows tested for a given comparison. Supplementary Figure 1 shows the distribution of the Z*F*_*ST*_ values. We set the top 0.1% positive values of Z*F*_*ST*_ as the threshold to identify the candidate regions under selection.

We also investigated signatures of selection using XP-EHH (Sabeti et al., 2007). It assesses haplotype differences between two test populations, and it can detect a wider range of selection scenarios, including selection on standing variation or incomplete sweeps or on-going selection (Sabeti et al., 2007; Innan and Kim, 2008). The test uses the integrated EHH (iHH) of a core SNP in two test populations, rather than two alleles in one test population. The unstandardized XP-EHH statistic is calculated as:

unstandardizedXP-EHH=ln(iHHA/iHHB)

where iHHA and iHHB are the integrated EHH of a given core SNP in the A and B test populations, respectively. A large and positive value of XP-EHH at a locus suggests selection in reference population while a negative value suggests selection in the alternate one. We used the software developed by Pickrell and Pritchard (2012) to estimate the unstandardized XP-EHH statistics for all SNPs in each comparison. These were then standardized using their means and variances, and the *p*-values were estimated from the standard normal distribution (Supplementary Figure 2). For each comparison, we determined the regions under selection based on the threshold *P* value < 0.001.

To investigate the functional significance of selection signatures, the candidate regions revealed by ROH, *F*_*ST*_, and XP-EHH were identified using the intersectBed function of BedTools software (Quinlan and Hall, 2010). The *O. aries* reference genome assembly (OAR_v3.0) was used to annotate the candidate regions using the BioMart Tool in Ensembl and NCBI databases. The functional annotation tool in DAVID v6.8 (Huang et al., 2009) was used to perform enrichment and gene ontology (GO) analyses using *O. aries* as the background. The functions of genes overlapping the candidate regions were also determined from literature.

## Results

### Genetic Diversity and Differentiation

The average values of H_*O*_ and H_*E*_ for the Sudanese thin-tailed Desert Sheep and the Chinese breeds exceeded 0.300 (Table 1). Among the Sudan thin-tailed Desert sheep, Hammari (HA) had the lowest values of H_*O*_ (0.341 ± 0.004) and H_*E*_ (0.343 ± 0.000) while the highest values were observed in Shanbali (SH) (H_*O*_ = 0.355 ± 0.008) and Kabashi (KA) (H_*E*_ = 0.357 ± 0.000). Among the Chinese breeds, the Black Tibetan (HZ) had the lowest values of H_*O*_ (0.336 ± 0.045) and H_*E*_ (0.332 ± 0.0001), whereas the highest values were present in Zeku (ZK) (H_*O*_ = 0.348 ± 0.005) and Tan Sheep (H_*E*_ = 0.360 ± 0.000). The average Pi-HAT value for the Sudan thin-tailed Desert Sheep was 0.056 ± 0.016, ranging between 0.081 and 0.112 (Table 1). This supported a low level of genetic relatedness between the individuals, which were confirmed by the low values of inbreeding (average *F*_*ROH*_ = 0.014 and *F* = 0.009). This validated our sampling strategy which targeted only mature unrelated animals. The Sudanese thin-tailed Desert Sheep had on average longer lengths of ROH, higher *F*_*ROH*_ but lower *F* compared to the Chinese breeds.

**FIGURE 1:**
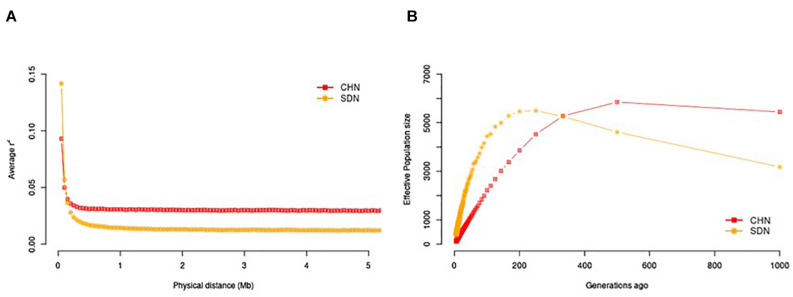
**(A)** LD decay pattern within 5 Mb distance in Sudanese and Chinese sheep. **(B)** N_*E*_ across 1,000 generations in Sudanese and Chinese sheep.

We investigated the trends in LD decay over genomic distances (Figure 1A) and in N_*E*_ over generation time (Figure 1B). Across all genomic distance intervals, the Sudanese thin-tailed Desert Sheep showed lower LD compared to the Chinese breeds. At 1,000 generations ago, the Sudanese thin-tailed Desert Sheep had lower N_*E*_ compared to the Chinese sheep. However, the thin-tailed Desert Sheep showed an expansion in N_*E*_ up to around 270 generations ago, after which it started to contract drastically up to the present time. The N_*E*_ for the Chinese breeds remained stable up to around 500 generations ago, upon which it also started to contract but at a gradual pace to the present time.

Genetic differentiation was assessed by calculating pairwise *F*_*ST*_ values between ecotypes and breeds (Supplementary Table 3). The overall highest levels of genetic differentiation were between the fat-tailed Tan Sheep against all the other ecotypes and breeds. Among the Sudanese thin-tailed Desert Sheep, AL-Ahamda (AL) showed the highest *F*_*ST*_ values with the other ecotypes while the lowest values were between Buzee (BU) with both HA and SH. Among the Chinese breeds, the lowest values of *F*_*ST*_ occurred between the thin-tailed Oula (QOL) and Zeku (ZK).

**FIGURE 2:**
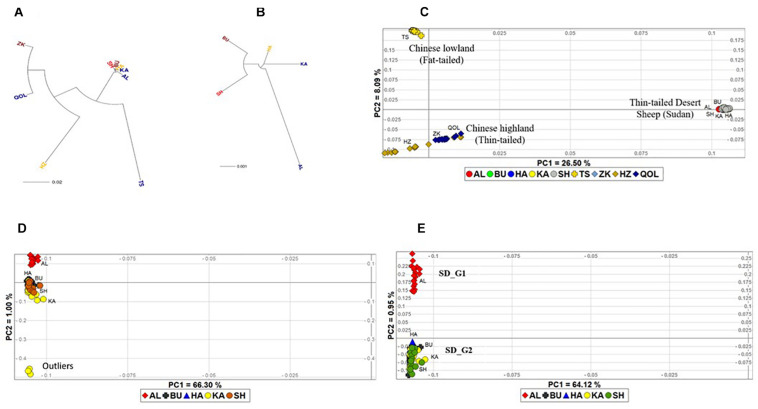
Neighbor Joining tree of **(A)** all the five Sudanese thin-tailed Desert Sheep ecotypes and four breeds of Chinese sheep; **(B)** the five ecotypes of Sudanese thin-tailed Desert Sheep only; **(C)** PCA plot of all the individuals analyzed in this study; **(D)** PCA of Sudanese thin tailed Desert Sheep only; **(E)** PCA of the Sudanese thin-tailed Desert Sheep excluding the three outliers of the Kabashi ecotype.

To visualize the extent of genetic differentiation, we generated an NJ phylogeny with the ecotypes and breeds at the nodes using the pairwise *F*_*ST*_ genetic distance matrix (Figure 2A). The tree revealed a clear separation of the fat-tailed Tan Sheep from the Chinese thin-tailed sheep and the Sudanese thin-tailed Desert Sheep. The latter clustered very close together, suggesting lower genetic differentiation. The HZ was separated from both ZK and QOL, suggesting a genetic divergence within the Chinese thin-tailed sheep. ZK and QOL had the lowest value of *F*_*ST*_ between them. An NJ phylogeny for the five ecotypes of Sudanese thin-tailed Desert Sheep was generated to obtain a better picture of the extent of their genetic relationships (Figure 2B). It showed that AL was genetically differentiated from BU, HA, KA, and SH.

We used individuals as the taxonomic units and performed a PCA to ascertain the divergence revealed by the NJ tree. In the first instance, all the study individuals were included in the PCA, and PC1 and PC2 explained 26.50 and 8.09% of the total genetic variation, respectively (Figure 2C). PC1 separated the Chinese breeds into two groups showing a marked concordance with their tail type and geographic origin. One group included the Tan Sheep (fat-tailed sheep from the lowlands, Ningxia Province) while the other group comprised HZ, QOL, and ZK (thin-tailed sheep from the alpine high-altitude, Qinghai Province). This grouping corresponded to the NJ phylogeny. Although HZ appeared to be separated from both QOL and ZK, its divergence was not as distinct as on the NJ tree. PC2 separated the Chinese breeds from the five ecotypes of Sudanese thin-tailed Desert Sheep which, as in the NJ tree, clustered close together. To further investigate the clustering pattern in the latter, we performed the PCA but excluding the Chinese breeds. It revealed three outlier individuals of KA and a slight separation of AL from the other ecotypes (Figure 2D). Given the likelihood that the three outliers could be masking the divergence of AL, we performed another PCA while excluding them. It resulted in a clear divergence of AL from the other ecotypes (Figure 2E). Given this result, we excluded the three outliers from subsequent analyses as their extreme divergence could not be explained.

**FIGURE 3:**
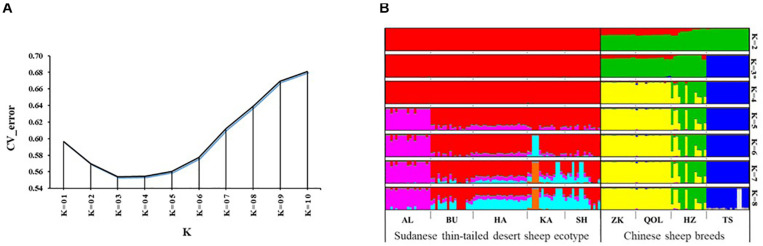
**(A)** CV plot of the admixture analysis involving all the samples analyzed in the study. **(B)** Admixture plot showing the assignment probabilities of all the individuals analyzed in the current study for 2 ≤ *K* ≤ 8.

We used the ADMIXTURE tool to investigate genome architecture and complement the NJ phylogeny and PCA. Running ADMIXTURE using all the study individuals resulted in the lowest CV error at *K* = 3 (Figure 3A). At this *K*, three gene pools were observed (Figure 3B). The first comprised the thin-tailed Desert Sheep from Sudan, the second was made up of the thin-tailed sheep from China (ZK, QOL, and HZ) and the third is exclusive to the Chinese fat-tailed Tan Sheep. We suggest that this reveals the broad-scale genetic structure defining the dataset. Considering the results of the NJ phylogeny and PCA, which showed AL to be genetically divergent and a slight separation of HZ from both ZK and QOL, we investigated the ADMIXTURE patterns at 4 ≤ *K* ≤ 8 (Figure 3B). At *K* = 4, HZ diverges from ZK and QOL and this divergence is retained up to *K* = 8. At *K* = 5, AL diverges from the other ecotypes of Sudanese thin-tailed Desert Sheep by its unique genetic background which is also retained up to *K* = 8. As the pattern of genetic structure remains consistent from 5 ≤ *K* ≤ 8 and it corresponds to the clusters revealed by the NJ phylogeny (Figures 2A,B) and PCA (Figures 2C,E), we suggest that *K* = 5 represents the fine-scale genomic structure in the study individuals. We therefore refer the two genetic clusters in the Sudanese thin-tailed Desert sheep as SD_G1 (AL) and SD_G2 (BU, HA, KA, and SH). We also classify the Chinese breeds into three genetic clusters, i.e., CN_G1 (Tan Sheep), CN_G2 (ZK and QOL), and CN_G3 (HZ).

### Signatures of Selection

Based on these genetic clusters, we used ROH, *F*_*ST*_ and XP-EHH to investigate the genetic signatures of their divergence. We performed the ROH analysis to identify ROH segments that were specific to SD_G1 or SD_G2. The *F*_*ST*_ and XP-EHH were used for comparative selection signature analyses that contrasted the two genetic clusters of the Sudanese thin-tailed Desert Sheep with each other and with two of the Chinese clusters. That was, for SD_G1, the following comparative analyses were performed: SD_G1 verses SD_G2, CN_G1, or CN_G2. A similar comparative analysis was performed for SD_G2. We excluded CN_G3 from these analyses because its genome showed an admixed profile. All the candidate regions and genes identified by each approach for each comparison involving SD_G1 and SD_G2 are shown in Supplementary Tables 4–10.

**FIGURE 4:**
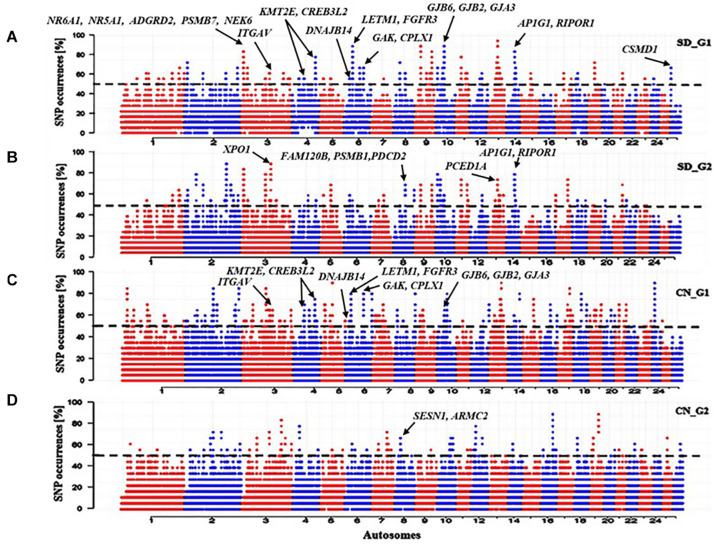
Manhattan plots of genome-wide distribution frequency of SNPs in stretches of ROH regions for **(A)** SD_G1 genetic group of thin-tailed Desert Sheep from Sudan; **(B)** SD_G2 genetic group of thin-tailed Desert Sheep from Sudan; **(C)** CN_G1 genetic group of Chinese sheep; **(D)** CN_G2 genetic group of Chinese sheep. The dashed lines indicate the 50% cut-off threshold for each groups of individuals.

The ROH analysis identified 107 ROH regions, overlapping 286 genes, that were specific to SD_G1 (Figure 4A), and 78 ROH regions, spanning 281 genes, that were specific to SD_G2 (Figure 4B). In total, 88 ROH regions were common between SD_G1 and SD_G2. The most significant ROH region which was common to SD_G1 and SD_G2 occurred on OAR3 (10,700,001–11,800,000 bp) and spanned 19 genes (Table 2). For the Chinese groups, 146 and 69 ROH islands spanning 257 and 43 genes were specific to CN_G1 (Figure 4C) and CN_G2 (Figure 4D), respectively.

**TABLE 2:** Candidate regions detected by at least two approaches of selection signature analysis in the SD_G1 (AL) sheep group.

Reg	Oar	Start	Stop	Size (Mb)	Method			Compression	No. of genes	Genes
1	1	222482061	222559568	0.078	ROH	XP-EHH	_	SD_G2	–	
2		25525099	25681293	0.156	ROH	_	*F*_*ST*_	SD_G2	4	*TTC39A*, *EPS15*, *LOC105609505*, *LOC105609934*
3		252663891	252805582	0.142	ROH	_	*F*_*ST*_	SD_G2	2	*KY*, *LOC105604327*
4		25525099	25681293	0.156	ROH	XP-EHH	_	CN_G2	2	*TTC39A*, *EPS15*
5		106050370	106283918	0.234	ROH	_	*F*_*ST*_	CN_G1	2	*ENSOARG00000006782*, *ENSOARG00000006800*
6		68580173	68776536	0.196	ROH	_	*F*_*ST*_	CN_G2	5	*BTBD8*, *U6*, *C1orf146*, *GLMN*, *RPAP2*
7	2	9128447	9157161	0.029	ROH	XP-EHH	_	CN_G1	1	*ATP6V1G1*
8		182815842	182824846	0.009	ROH	XP-EHH	_	CN_G1	–	
9		14616917	14797915	0.181	ROH	XP-EHH	_	CN_G2	1	*U6*
10	3	10700001	11800000	1.099	ROH	XP-EHH	_	CN_G1, CN_G2	19	*HSPA5*, *RABEPK*, *PPP6C*, *SCAI*, *ENSOARG00000025028*, *GOLGA1*, *ARPC5L*, *ENSOARG00000013275*, *WDR38*, *U6*, *OLFML2A*, *ENSOARG00000023155*, *oar-mir-181a-2*, *NR6A1*, *NR5A1*, *ADGRD2*, *PSMB7*, *NEK6*, *LHX2*
11		131800001	132600000	0.799	ROH	XP-EHH	_	SD_G2	19	*PPP1R1A*, *PDE1B*, *NCKAP1L*, *GTSF1*, *ITGA5*, *ZNF385A*, *COPZ1*, *NFE2*, *CBX5*, *HOXC4*, *HOXC5*, *HOXC6*, *HOXC8*, *HOXC9*, *HOXC10*, *HOXC11*, *HOXC12*, *HOXC13*, *STAB2*
12		12404574	12497707	0.093	ROH	XP-EHH	_	CN_G1, CN_G2	1	*U6*
13		206745061	206865854	0.121	ROH	XP-EHH	_	CN_G1	4	*LOC105614286*, *LOC105608615*, *LOC105608603*, *LOC105614853*
14		135904027	135905257	0.001	ROH	XP-EHH	_	CN_G2	1	*LIMA1*
15		135983081	135983081	0.000	ROH	XP-EHH	_	CN_G2	1	*CERS5*
16		153813836	153863913	0.050	ROH	_	*F*_*ST*_	CN_G1	3	*ENSOARG00000002929*, *ENSOARG00000023603*, *LOC105609946*
17		185666225	185873353	0.207	ROH	_	*F*_*ST*_	CN_G1	–	
18	5	107400001	108000000	0.599	_	_	*F*_*ST*_	SD_G2, CN_G1	5	*ENSOARG00000000146*, *CAMK4*, *STARD4*, *ENSOARG00000010495*, *WDR36*, *ENSOARG00000025339*, *ENSOARG00000025340*, *ENSOARG00000025340*
19		41700001	42000000	0.299	_	XP-EHH	_	CN_G2	11	*ENSOARG00000013233*, *ENSOARG00000013103*, *SOWAHA*, *SHROOM1*, *GDF9*, *UQCRQ*, *LEAP2*, *AFF4*, *U6*, *ZCCHC10*, *ENSOARG00000013524*
20		49700001	50000000	0.299	_	XP-EHH	_	CN_G2	10	*PCDHB14*, *PCDHB15*, *ENSOARG00000014442*, *TAF7*, *PCDHGA1*, *PCDHGA2*, *ENSOARG00000000218*, *PCDHGC3*, *ENSOARG00000023940*, *DIAPH1*
21		48997231	49137648	0.140	ROH	XP-EHH	_	CN_G2	9	*LOC101121602*, *5S_rRNA*, *U6*, *SRA1*, *APBB3*, *LOC105615270*, *AO21*, *AO45*, *SLC35A4*, *ENSOARG00000018230*
22		49139142	49171418	0.032	ROH	XP-EHH	_	CN_G2	5	*ENSOARG00000018230*, *CD14*, *TMCO6*, *NDUFA2*, *IK*
23		49589412	49704569	0.115	ROH	XP-EHH	_	CN_G2	5	*PCDHB6*, *PCDHB7*, *LOC101103233*, *LOC101102062*, *PCDHB14*
24	6	69896247	70000135	0.104	ROH	XP-EHH	*F*_*ST*_	SD_G2	2	*LOC105613062*, *LOC105613064*
25		85447324	85695088	0.248	ROH	XP-EHH	*F*_*ST*_	SD_G2, CN_G1, CN_G2	6	*ENSOARG00000011228*, *ENSOARG00000008596*, *AMTN*, *AMBN*, *ENAM*, *JCHAIN*
26		116051931	116125336	0.073	ROH	_	*F*_*ST*_	SD_G2	4	*NSD2*, *LETM1*, *FGFR3*, *ENSOARG00000015218*
27		69700001	70000000	0.300	ROH	XP-EHH	*F*_*ST*_	SD_G2, CN_G2	2	*PDGFRA*, *ENSOARG00000021645*
28		73500001	73900000	0.400	_	XP-EHH	*F*_*ST*_	SD_G2	2	*LOC101118699*, *TRNAC-GCA*
29		83200001	83600000	0.400	_	XP-EHH	*F*_*ST*_	SD_G2	7	*CENPC*, *STAP1*, *UBA6*, *GNRHR*, *ENSOARG00000007652*, *TMPRSS11D*, *TMPRSS11A*
30		69896247	70000135	0.104	ROH	_	*F*_*ST*_	CN_G2	2	*LOC105613062*, *LOC105613064*
31	8	27957061	28318981	0.362	ROH	XP-EHH	_	CN_G1	8	*ENSOARG00000010506*, *ENSOARG00000010556*, *ENSOARG00000010591*, *CEP57L1*, *SESN1*, *5S_rRNA*, *U12*, *ARMC2*
32	10	71700001	72100000	0.400	_	XP-EHH	*F*_*ST*_	SD_G2	5	*LOC101109370*, *LOC101109105*, *LOC105616210*, *LOC101107612*, *LOC101109626*
33		36524922	36651187	0.126	ROH	XP-EHH	_	CN_G2	4	*MPHOSPH8*, *PARP4*, *ENSOARG00000024226*, *U6*
34		70812659	70863686	0.051	ROH	_	*F*_*ST*_	CN_G1, CN_G2	2	*ENSOARG00000001156*, *ENSOARG00000001163*
35	11	24600001	24800000	0.200	ROH	_	*F*_*ST*_	CN_G1	7	*SPNS2*, *MYBBP1A*, *GGT6*, *TEKT1*, *SMTNL2*, *FBXO39*, *XAF1*
36	13	56200001	56600000	0.399	_	_	*F*_*ST*_	SD_G1, CN_G1	3	*PHACTR3*, *ENSOARG00000015902*, *ZNF831*
37		42500001	42800000	0.300	ROH	_	*F*_*ST*_	CN_G1	8	*LOC101109370*, *LOC101109105*, *LOC105616210*, *LOC101107612*, *LOC101109626*, *LOC101109633*, *LOC101109111*, *LOC101109377*
38	14	34400001	34600000	0.200	ROH	XP-EHH	*F*_*ST*_	CN_G2	10	*ATP6V0D1*, *AGRP*, *RIPOR1*, *CTCF*, *CARMIL2*, *ACD*, *PARD6A*, *ENKD1*, *C16orf86*, *GFOD2*
39		38400001	38600000	1.999	ROH	XP-EHH	_	CN_G1	6	*PKD1L3*, *IST1*, *U6*, *ZNF821*, *ATXN1L*, *AP1G1*
40	15	42448209	42461989	0.014	ROH	_	*F*_*ST*_	CN_G2	1	*SBF2*
41	17	34524230	34545454	0.021	ROH	XP-EHH	*F*_*ST*_	SD_G2	1	*FGF2*
42		34400001	34700000	0.300	_	XP-EHH	*F*_*ST*_	SD_G2	5	*FGF2*, *ENSOARG00000023095*, *NUDT6*, *U4*, *LOC101104012*
43		34524230	34545454	0.021	ROH	XP-EHH	_	CN_G2	1	*FGF2*
44	18	17316593	17437561	0.121	ROH	XP-EHH	_	CN_G1, CN_G2	1	*LOC105603074*
45		23475785	23580801	0.105	ROH	_	*F*_*ST*_	SD_G2, CN_G2	1	*EFL1 (EFTUD1)*
46	21	36959560	37178672	0.219	ROH	_	*F*_*ST*_	CN_G2	6	*PAG11*, *LOC105604177*, *LOC101123081*, *PAG3*, *LOC105604087*, *LOC105604088*
47	26	1506153	1925293	0.419	ROH	XP-EHH	_	CN_G1, CN_G2	1	*CSMD1*
48		1344691	1394564	0.050	ROH	XP-EHH	_	CN_G2	1	*ENSOARG00000026782*

*Reg = candidate region; Oar = ovine chromosome.*

**FIGURE 5:**
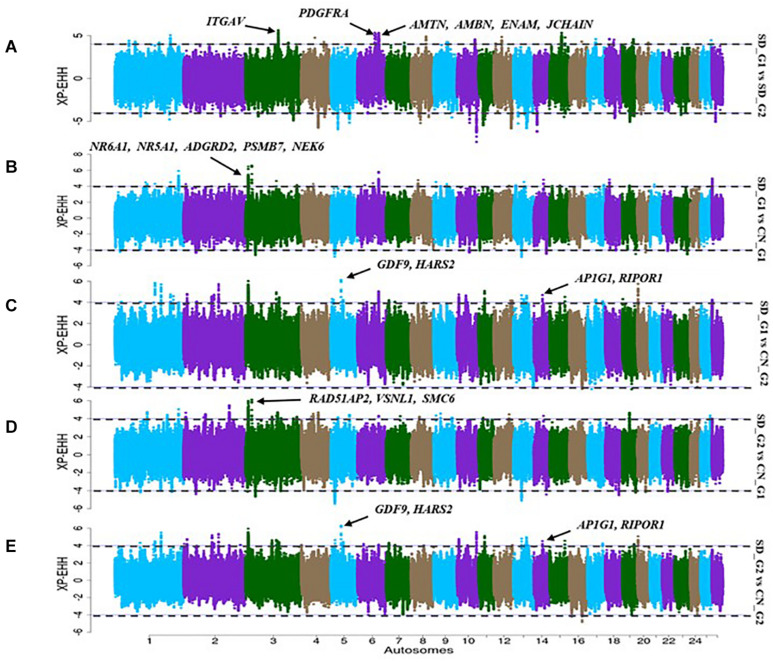
Manhattan plots showing the candidate signatures of selection as determined with *XP-EHH.* **(A)** SD_G1 vs. SD_G2; **(B)** SD_G1 vs. CN_G1; **(C)** SD_G1 vs. CN_G2; **(D)** SD_G2 vs. CN_G1; **(E)** SD_G2 vs. CN_G2.

The XP-EHH identified 32 candidate regions in the comparative analysis between SD_G1 and SD_G2 (Figure 5A). These regions spanned 73 putative genes and the most significant region was the same as that identified by ROH on OAR3 (Table 2). Between SD_G1 and CN_G1, XP-EHH identified 34 candidate regions (Figure 5B) and against CN_G2, it identified 46 candidate regions (Figure 5C). These regions spanned 83 and 225 genes, respectively. The most significant region with CN_G1 was on OAR6 (85,447,324–85,695,088 bp) and spanned six genes while the one with CN_G2 occurred on OAR14 (34,400,001–34,600,000 bp) and spanned 10 genes (Table 2). The XP-EHH analysis between SD_G2 and CN_G1 (Figure 5D) or CN_G2 (Figure 5E) identified 31 and 46 candidate regions which spanned 83 and 208 genes, respectively. The most significant regions occurred on OAR10 (78,200,001–78,500,000 bp) and OAR14 (34,400,001–34,600,000 bp) spanning 7 and 10 putative genes (Table 3), respectively.

**TABLE 3:** Candidate regions detected by at least two approaches of selection signature analysis in the SD_G2 sheep group.

Reg	Oar	Start	Stop	Size (Mb)	Method			Compression	No. of genes	Genes
1	1	102680214	102786943	0.107	ROH	XP-EHH	_	SD_G1	3	*NPR1*, *INTS3*, *SLC27A3*
2		105567842	105641892	0.074	ROH	_	*F*_*ST*_	CN_G1	3	*ENSOARG00000022142*, *ENSOARG00000006704*, *ETV3*
3		129000001	129200000	0.200	_	XP_EHH	*F*_*ST*_	CN_G1	3	*MRPL39*, *MIR155*, *U6*
4		255100001	255300000	0.200	_	XP_EHH	*F*_*ST*_	CN_G1	4	*ACAD11*, *ACKR4*, *DNAJC13*, *ENSOARG00000009070*
5	2	184000001	184500000	0.500	_	XP_EHH	*F*_*ST*_	CN_G1	5	*PTPN4*, *EPB41L5*, *U4*, *TMEM185B*, *RALB*
6	3	10700001	11800000	1.099	ROH	XP-EHH	_	CN_G1, CN_G2	19	*HSPA5*, *RABEPK*, *PPP6C*, *SCAI*, *ENSOARG00000025028*, *GOLGA1*, *ARPC5L*, *ENSOARG00000013275*, *WDR38*, *U6*, *OLFML2A*, *ENSOARG00000023155*, *oar-mir-181a-2*, *NR6A1*, *NR5A1*, *ADGRD2*, *PSMB7*, *NEK6*, *LHX2*
7		192700001	193100000	0.400	_	XP-EHH	*F*_*ST*_	SD_G1	5	*ST8SIA1*, *ENSOARG00000023574*, *LOC101115359*, *CMAS*, *ABCC9*
8		11763552	11825668	0.062	ROH	XP_EHH	_	CN_G1, CN_G2	2	*DENND1*, *ENSOARG00000013754*
9		10512650	10583705	0.071	ROH	XP_EHH	_	CN_G2	2	*MAPKAP1*, *U5*
10		107100001	107300000	0.200	_	XP_EHH	*F*_*ST*_	CN_G1	1	*TSPAN8*
11		129700001	129900000	0.200	_	XP_EHH	*F*_*ST*_	CN_G1, CN_G2	2	*SOCS2*, *CRADD*
12	4	94273495	94445213	0.172	ROH	XP-EHH	_	SD_G1	10	*ENSOARG00000022095*, *ENSOARG00000021327*, *ENSOARG00000022560*, *MEST*, *MIR335*, *COPG2*, *ENSOARG00000025252*, *ENSOARG00000025253*, *ENSOARG00000025253*, *TSGA13*
13		87332113	87348849	0.017	ROH	_	*F*_*ST*_	SD_G1	–	
14		48500001	48800000	0.300	_	XP_EHH	*F*_*ST*_	CN_G1	8	*COG5*, *GPR22*, *DUS4L*, *ENSOARG00000005784*, *ENSOARG00000024106*, *SLC26A4*, *CBLL1*, *SLC26A3*
15	5	41700001	42000000	0.299	_	XP-EHH	_	CN_G2	11	*ENSOARG00000013233*, *ENSOARG00000013103*, *SOWAHA*, *SHROOM1*, *GDF9*, *UQCRQ*, *LEAP2*, *AFF4*, *U6*, *ZCCHC10*, *ENSOARG00000013524*
16		49700001	50000000	0.299	_	XP-EHH	_	CN_G2	10	*PCDHB14*, *PCDHB15*, *ENSOARG00000014442*, *TAF7*, *PCDHGA1*, *PCDHGA2*, *ENSOARG00000000218*, *PCDHGC3*, *ENSOARG00000023940*, *DIAPH1*
17		107463423	107559150	0.096	ROH	_	*F*_*ST*_	SD_G1	2	*CAMK4*, *ENSOARG00000025339*
18		28500001	28900000	0.400	_	XP-EHH	*F*_*ST*_	SD_G1	1	*SNCAIP*
19		74969987	75083385	0.113	ROH	XP_EHH	_	CN_G1	1	*LOC105606744*
20		49868921	49929170	0.060	ROH	XP_EHH	_	CN_G2	2	*LOC101105495*, *LOC101104318*
21	6	24953079	24968246	0.015	ROH	XP_EHH	_	CN_G2	1	*LOC105615434*
22	7	55764564	55901243	0.137	ROH	XP_EHH	_	CN_G1	1	*DMXL2*
23		34293259	34382138	0.089	ROH	XP_EHH	_	CN_G2	2	*SPTBN5*, *EHD4*
24	8	90416289	90680086	0.264	ROH	_	*F*_*ST*_	SD_G1, CN_G1, CN_G2	8	*ENSOARG00000005108*, *FAM120B*, *PSMB1*, *ENSOARG00000005142*, *PDCD2*, *ENSOARG00000027055*, *ENSOARG00000027056*, *ENSOARG00000027057*
25	10	50000001	50400000	0.400	_	XP-EHH	*F*_*ST*_	SD_G1	–	
26		78200001	78500000	0.300	_	XP-EHH	*F*_*ST*_	SD_G1	7	*TPP2*, *METTL21C*, *ENSOARG00000005160*, *TEX30*, *POGLUT2 (KDELC1)*, *ERCC5*, *LOC101114712*
27		7300001	7600000	0.300	_	XP_EHH	*F*_*ST*_	CN_G2	2	*ENSOARG00000006632*, *ENSOARG00000006641*
28	11	51283288	51361005	0.078	ROH	_	*F*_*ST*_	CN_G2	1	*RNF213*
29	12	69500001	70100000	0.600	_	XP-EHH	*F*_*ST*_	SD_G1	7	*DTL*, *INTS7*, *LPGAT1*, *ENSOARG00000022944*, *NEK2*, *ENSOARG00000004286*, *SLC30A1*
30		61370423	61387625	0.017	ROH	XP_EHH	_	CN_G1	–	
31	13	56326781	56522417	0.196	ROH	XP-EHH	*F*_*ST*_	SD_G1	2	*ENSOARG00000015902*, *ZNF831*
32		46300001	46700000	0.400	_	XP-EHH	*F*_*ST*_	SD_G1	6	*RASSF2*, *SLC23A2*, *TMEM230*, *PCNA*, *CDS2*, *ENSOARG00000022618*
33		55900001	56400000	0.500	_	XP-EHH	*F*_*ST*_	SD_G1	6	*FAM217B*, *PPP1R3D*, *SYCP2*, *PHACTR3*, *ENSOARG00000021945*, *ENSOARG00000015902*
34		56400001	56700000	0.300	_	XP-EHH	*F*_*ST*_	SD_G1	7	*ENSOARG00000015902*, *ZNF831*, *ZNF831*, *PRELID3B*, *ATP5F1E*, *TUBB1*, *CTSZ*
35		33415419	33514937	0.100	ROH	XP_EHH	_	CN_G2	1	*ZNF438*
36	14	34400001	34600000	0.200	ROH	XP-EHH	_	CN_G2	10	*ATP6V0D1*, *AGRP*, *RIPOR1*, *CTCF*, *CARMIL2*, *ACD*, *PARD6A*, *ENKD1*, *C16orf86*, *GFOD2*
37		38400001	38600000	1.999	ROH	XP-EHH	_	CN_G1	6	*PKD1L3*, *IST1*, *U6*, *ZNF821*, *ATXN1L*, *AP1G1*
38	16	70528271	70733910	0.206	ROH	_	*F*_*ST*_	CN_G1	8	*ENSOARG00000026989*, *ENSOARG00000015678*, *ENSOARG00000015729*, *ENSOARG00000015756*, *5S_rRNA*, *EXOC3*, *ENSOARG00000026990*, *SLC9A3*
39	18	19336673	19349659	0.013	ROH	_	*F*_*ST*_	CN_G2	1	*LOC101122139*
40	19	31400001	31700000	0.300	_	XP-EHH	*F*_*ST*_	SD_G1	2	*ENSOARG00000009783*, *MITF*
41		37200001	37700000	0.500	_	XP-EHH	*F*_*ST*_	SD_G1	6	*PRICKLE2*, *PSMD6*, *ATXN7*, *THOC7*, *C3orf49*, *ENSOARG00000011488*
42		48100001	48400000	0.300	_	XP-EHH	*F*_*ST*_	SD_G1	15	*NEK4*, *ENSOARG00000024525*, *SPCS1*, *GLT8D1*, *GNL3*, *SNORD69*, *SNORD19C*, *SNORD19B*, *SNORD19*, *PBRM1*, *PBRM1*, *SMIM4*, *NT5DC2*, *_*ST*_AB1*, *NISCH*
43		47900001	48100000	0.200	_	XP_EHH	*F*_*ST*_	CN_G2	6	*SFMBT1*, *ENSOARG00000000541*, *ENSOARG00000000606*, *ITIH4*, *ITIH3*, *ITIH1*
44	20	16646531	16720282	0.074	ROH	XP-EHH	*F*_*ST*_	SD_G1	7	*PTCRA*, *CNPY3*, *GNMT*, *PEX6*, *PPP2R5D*, *MEA1*, *KLHDC3*
45	21	46517968	46631317	0.113	ROH	_	*F*_*ST*_	SD_G1	1	*ANO1*
46		38518624	38519133	0.001	ROH	_	*F*_*ST*_	CN_G2	1	*ENSOARG00000026107*
47	25	39166409	39205820	0.039	ROH	XP_EHH	_	CN_G1	1	*ENSOARG00000000408 (LOC101114083)*

*Reg = candidate region; Oar = ovine chromosome.*

**FIGURE 6:**
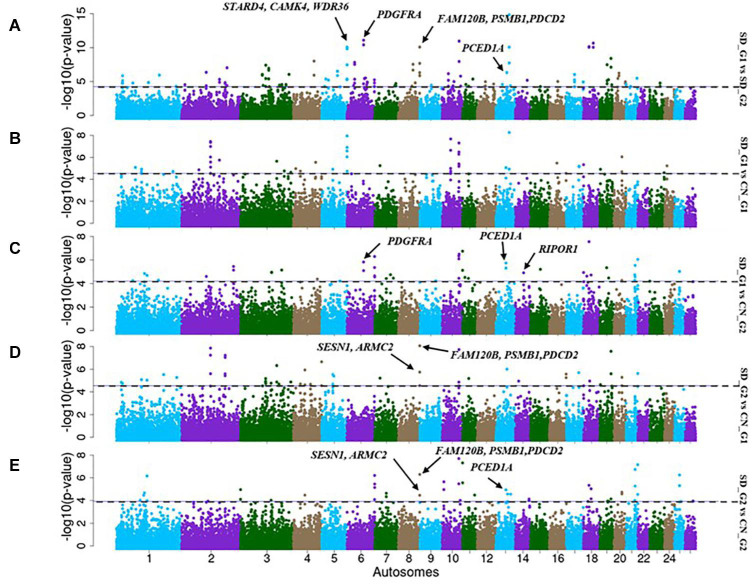
Manhattan plots showing the candidate signatures of selection as determined with *F*_*ST*_. **(A)** SD_G1 vs. SD_G2; **(B)** SD_G1 vs. CN_G1; **(C)** SD_G1 vs. CN_G2; **(D)** SD_G2 vs. CN_G1; **(E)** SD_G2 vs. CN_G2.

The *F*_*ST*_ analysis identified 73 candidate regions with extreme allele frequency differentiation between SD_G1 and SD_G2 (Figure 6A), which spanned 288 putative genes. The most significant region was on OAR6 (69,700,001–70,000,000 bp) spanning two genes (Table 2). Between SD_G1 and CN_G1, *F*_*ST*_ revealed 24 regions spanning 56 genes (Figure 6B) and between SD_G1 and CN_G2, it identified 38 regions spanning 89 genes (Figure 6C). For SD_G2, *F*_*ST*_ identified 35 and 33 candidate regions that differentiated the group with CN_G1 and CN_G2, respectively. These regions spanned 107 and 68 genes, respectively, and the most significant regions occurred on OAR20 (16,646,531–16,720,282 bp) and OAR3 (129,700,001–129,900,000 bp) spanning seven and two genes (Table 3), respectively.

In general, there were 48 candidate regions in SD_G1, overlapping 206 genes that were simultaneously identified by a combination of at least two methods (ROH, *F*_*ST*_ and/or XP-EHH) and/or two comparative analyses (SD_G1 verses SD_G2/CN_G1/CN_G2) (Table 2). Among these regions, 39 were identified by ROH with either *F*_*ST*_ or XP-EHH, four by all the three approaches and five by *F*_*ST*_ and XP-EHH. For SD_G2, 47 candidate regions overlapping 209 genes across 18 autosomes were identified by a combination of at least two approaches and/or two comparative analyses (Table 3). Among these regions, 24 were identified by a combination of ROH with either *F*_*ST*_ or XP-EHH, two by all the three approaches and 19 by *F*_*ST*_ and XP-EHH.

The three approaches (ROH, XP-EHH, and *F*_*ST*_) identified a total of 697 and 765 putative genes that overlapped with candidate regions identified in SD_G1 (Table 4) and SD_G2 (Table 5), respectively. These genes were used for GO and enrichment analyses for each group and the top-most significant GO terms and KEGG pathways are shown in Tables 4, 5. The “hyaluronan metabolic process (GO:0030212)” is the most common GO term across the comparisons. Since it is associated with a wide range of functions (Stern et al., 2006), it may be relevant to the two groups of Sudanese thin-tailed Desert sheep.

**TABLE 4:** Enriched functional terms and their enrichment scores following *DAVID* analysis for genes identified by all methodologies in the SD_G1 (AL) Sudanese sheep group.

Group	Type	Category	TermGene count	*P* value	Genes	Benjamini
SD_G1 vs. SD_G2 (334 annotated genes)	GOTERM_BP_DIRECT	GO:0009952	Anterior/posterior pattern specification10	0.00001	*HOXC5*, *HOXC4*, *HOXC9*, *GRSF1*, *HOXC8*, *HOXC11*, *GLI3*, *HOXC10*, *ACVR2A*, *HOXC6*	0.00933
	GOTERM_MF_DIRECT	GO:0004861	Cyclin-dependent protein serine/threonine kinase inhibitor activity4	0.00042	*CDKN2D*, *KAT2B*, *CDKN2C*, *INCA1*	0.08136
	GOTERM_CC_DIRECT	GO:0005737	Cytoplasm58	0.00052	*SLC23A2*, *STAB2*, *SRA1*, *HOXC11*, *EFTUD2*, *EPB41L4B*, *PPP2R5E*, *SESN1*, *MMP28*, *MEIOC*, *ARIH1*, *TRIM21*, *PDGFRA*, *PARP4*, *ARID1B*, *GLTPD2*, *RNF167*, *NEIL2*, *RUFY3*, *SLFN14*, *PICK1*, *CACTIN*, *DTL*, *ARF5*, *GTSF1*, *ATG4D*, *GTF2A1*, *ITIH4*, *UBA6*, *SRF*, *PRICKLE2*, *DBF4B*, *PLA2G6*, *RPAP2*, *CENPC*, *EPB41L5*, *ORC4*, *STK36*, *PSMB1*, *STXBP5*, *APBB3*, *HOXC6*, *CDKN2D*, *KLHDC3*, *RRM1*, *ZFHX3*, *TGFB1*, *PPIL2*, *CDKN2C*, *STAT1*, *SNCAIP*, *ACVR2A*, *COPS2*, *NF1*, *TPP2*, *PDCD2*, *PTPN4*, *MPHOSPH8*	0.10205
	KEGG_PATHWAY	oas05152	Tuberculosis10	0.00244	*TGFB1*, *SC5*, *STAT1*, *IFNGR1*, *CATHL3*, *AKT3*, *MAPK1*, *CD14*, *FADD*, *BAC5*	0.27089
	GOTERM_MF_DIRECT	GO:0043022	Ribosome binding5	0.00255	*LETM1*, *SPCS1*, *EFL1*, *SLFN14*, *RICTOR*	0.24848
	KEGG_PATHWAY	oas05212	Pancreatic cancer6	0.00279	*RALB*, *TGFB1*, *STAT1*, *AKT3*, *TGFA*, *MAPK1*	0.27089
	GOTERM_BP_DIRECT	GO:0030212	Hyaluronan metabolic process3	0.00407	*ITIH4*, *ITIH3*, *ITIH1*	1.00000
	GOTERM_CC_DIRECT	GO:0005730	Nucleolus19	0.00507	*ZFHX3*, *WDR36*, *IK*, *CBX5*, *RNMT*, *UTP3*, *STAT1*, *NAT10*, *KRI1*, *RPAP2*, *GNL3*, *ORC4*, *EXOSC5*, *NOVA1*, *CAMK4*, *TRIM68*, *NOL10*, *DTL*, *MPHOSPH8*	0.49461
	GOTERM_MF_DIRECT	GO:0008289	Lipid binding6	0.00607	*BPIFA1*, *STARD4*, *BPIFB1*, *FABP5*, *BPIFA3*, *FABP12*	0.39428
	GOTERM_BP_DIRECT	GO:0019985	Translesion synthesis3	0.00832	*POLN*, *SPRTN*, *DTL*	1.00000
SD_G1 vs. CN_G1 (224 annotated genes)	GOTERM_BP_DIRECT	GO:0045921	Positive regulation of exocytosis3	0.01215	*CADPS2*, *VSNL1*, *CFTR*	1.00000
	GOTERM_CC_DIRECT	GO:0005922	Connexon complex3	0.01325	*GJB2*, *GJA3*, *GJB6*	1.00000
	GOTERM_MF_DIRECT	GO:0030345	Structural constituent of tooth enamel2	0.02370	*ENAM*, *AMBN*	1.00000
	GOTERM_BP_DIRECT	GO:0070175	Positive regulation of enamel mineralization2	0.02610	*ENAM*, *AMTN*	1.00000
	GOTERM_CC_DIRECT	GO:0005730	Nucleolus12	0.02915	*RSL1D1*, *ZFHX3*, *WDR36*, *IK*, *PSPC1*, *CAMK4*, *ATXN1L*, *CRYL1*, *NOL10*, *SNRPB2*, *RPAP2*, *MPHOSPH8*	1.00000
SD_G1 vs. CN_G2 (365 annotated genes)	GOTERM_CC_DIRECT	GO:0005737	Cytoplasm60	0.00031	GMEB2, BICDL1, SRA1, STMN3, MSI1, HOXC11, XPO4, SIN3A, SESN1, RNF17, ARIH1, AZI2, KPNA1, PARP4, DTX3L, MYT1, ARID1B, COMMD4, ASPM, LATS2, TIPRL, ZNF438, PICK1, TLN1, NEIL1, ACTL7B, PXN, TUBD1, PLA2G6, RPAP2, LIMA1, PSMB7, NUAK1, STK36, DND1, APBB3, GCN1, SCAI, HOXC6, GINS1, PTPN18, ACD, ZFHX3, SRMS, SPAG8, NEK6, PTK6, PARP14, PTPN14, MSMP, GDF9, CENPF, CDAN1, MYBBP1A, COPS2, NF1, PTPN9, MPHOSPH8, EEF1AKMT1, CDK5R2	0.06403
	GOTERM_BP_DIRECT	GO:0009952	Anterior/posterior pattern specification8	0.00050	*HOXC5*, *HOXC4*, *HOXC9*, *HOXC8*, *HOXC11*, *GLI3*, *HOXC10*, *HOXC6*	0.37470
	GOTERM_MF_DIRECT	GO:0003950	NAD+ ADP-ribosyltransferase activity4	0.00244	*PARP4*, *SIRT4*, *PARP9*, *PARP14*	0.44686
	GOTERM_BP_DIRECT	GO:0007194	Negative regulation of adenylate cyclase activity3	0.00839	*P2RY13*, *GPR87*, *DRD3*	1.00000
	GOTERM_MF_DIRECT	GO:0043565	Sequence-specific DNA binding11	0.01275	*NR5A1*, *NFE2*, *ZFHX3*, *ZGPAT*, *LHX2*, *FEV*, *HOXC5*, *HOXC4*, *HOXC9*, *HOXC8*, *HOXC6*	0.87513
	GOTERM_BP_DIRECT	GO:0019731	Antibacterial humoral response3	0.01400	*PLA2G1B*, *PLA2G6*, *JCHAIN*	1.00000
	GOTERM_MF_DIRECT	GO:0043022	Ribosome binding4	0.01621	*LETM1*, *HSPA5*, *EFL1*, *GCN1*	0.87513
	GOTERM_CC_DIRECT	GO:0008180	COP9 signalosome4	0.01830	*STOML2*, *HSPA5*, *COPS2*, *DOCK7*	0.97140
	KEGG_PATHWAY	oas04721	Synaptic vesicle cycle4	0.04776	*ATP6V1G1*, *CLTC*, *ATP6V0D1*, *CPLX1*	1.00000

**TABLE 5:** Enriched functional terms and their enrichment scores following *DAVID* analysis for genes identified by all methodologies in the SD_G2 (BU, HA, KA, and SH) Sudanese sheep group.

Group	Type	Category	TermGene count	*P* value	Genes	Benjamini
SD_G2 vs. SD_G1 (455 annotated genes)	GOTERM_CC_DIRECT	GO:0005737	Cytoplasm77	0.00016	*SLC23A2*, *LPGAT1*, *PREP*, *OXT*, *DNPH1*, *EFTUD2*, *HOXA10*, *EPB41L4B*, *XPO1*, *RASSF2*, *SOX15*, *MMP28*, *TUBB1*, *XPO5*, *MEIOC*, *POLH*, *PNISR*, *PDGFRA*, *USP45*, *ATP1B2*, *MYT1*, *THOC7*, *ENAH*, *GLTPD2*, *RNF167*, *NEIL2*, *IRF4*, *RUFY3*, *SLFN14*, *ZNF438*, *PRKD1*, *CACTIN*, *TP53*, *KIZ*, *DTL*, *ATG4D*, *GTF2A1*, *ITIH4*, *SPTBN5*, *PCNA*, *UBA6*, *TUBGCP2*, *SATB2*, *SRF*, *PRICKLE2*, *CACNA1A*, *DBF4B*, *PSMB10*, *CENPC*, *EPB41L5*, *ORC4*, *FXR2*, *ATXN7*, *PSMB1*, *STXBP5*, *TSNAXIP1*, *DTD2*, *GINS1*, *CDKN2D*, *KLHDC3*, *NOP58*, *TGFB1*, *PPIL2*, *CDKN2C*, *STAT1*, *MICAL3*, *MAD2L1BP*, *KLHL2*, *SNCAIP*, *ACVR2A*, *GNMT*, *NSFL1C*, *TPP2*, *PDCD2*, *PTPN4*, *TUBA8*, *WRAP53*	0.04116
	GOTERM_MF_DIRECT	GO:0043022	Ribosome binding6	0.00083	*LETM1*, *YTHDF1*, *SPCS1*, *EFL1*, *SLFN14*, *RICTOR*	0.21739
	KEGG_PATHWAY	oas04925	Aldosterone synthesis and secretion7	0.00587	*MC2R*, *NPR1*, *CAMK4*, *CREB3L2*, *ITPR2*, *CACNA1D*, *PRKD1*	0.95128
	GOTERM_CC_DIRECT	GO:0005654	Nucleoplasm46	0.00644	*PCNA*, *RNMT*, *TUBGCP2*, *MAPKAP1*, *KEAP1*, *DBF4B*, *IRF2BPL*, *EDC4*, *ORC4*, *GGA1*, *DHX33*, *CREB3L2*, *XPO5*, *PHACTR3*, *HMG20B*, *MAPK1*, *NUP88*, *POLH*, *CDKN2D*, *PNISR*, *PBRM1*, *PHC2*, *PPIL2*, *PHC1*, *POLN*, *SFMBT1*, *MICAL3*, *TMEM192*, *EVX1*, *ILF2*, *MYT1*, *THOC7*, *GATAD2B*, *GNL3*, *NSFL1C*, *STAG1*, *PRPF6*, *IRF4*, *CAMK4*, *GID8*, *ZNF438*, *TCEA2*, *RANGRF*, *CACTIN*, *TKT*, *DTL*	0.65751
	GOTERM_BP_DIRECT	GO:0030212	Hyaluronan metabolic process3	0.00746	*ITIH4*, *ITIH3*, *ITIH1*	1.00000
	GOTERM_CC_DIRECT	GO:0072562	Blood microparticle7	0.00777	*ITIH4*, *TGFB1*, *AHSG*, *HRG*, *ITIH1*, *KNG1*, *JCHAIN*	0.65751
	GOTERM_BP_DIRECT	GO:0006611	Protein export from nucleus4	0.00781	*XPO1*, *TGFB1*, *XPO5*, *NUTF2*	1.00000
	KEGG_PATHWAY	oas05212	Pancreatic cancer6	0.01308	*RALB*, *TGFB1*, *STAT1*, *TGFA*, *MAPK1*, *TP53*	0.95128
	GOTERM_MF_DIRECT	GO:0004869	Cysteine-type endopeptidase inhibitor activity4	0.01447	*AHSG*, *FETUB*, *HRG*, *KNG1*	1.00000
SD_G2 vs. CN_G1 (282 annotated genes)	GOTERM_BP_DIRECT	GO:0007265	Ras protein signal transduction5	0.00207	*RALB*, *CDK2*, *NF1*, *HRAS*, *TP53*	0.93013
	GOTERM_BP_DIRECT	GO:0030212	Hyaluronan metabolic process3	0.00302	*ITIH4*, *ITIH3*, *ITIH1*	0.93013
	GOTERM_BP_DIRECT	GO:0007093	Mitotic cell cycle checkpoint4	0.00431	*INTS3*, *MAD2L1BP*, *KNTC1*, *HRAS*	0.93013
	GOTERM_CC_DIRECT	GO:0070062	Extracellular exosome47	0.00828	*CRB2*, *FBLN7*, *NDUFB9*, *ITIH4*, *RAB5B*, *ITIH3*, *SPTBN5*, *RALB*, *ECHS1*, *CAB39L*, *ARPC5L*, *PDXP*, *LCAT*, *SAT2*, *PKD1L3*, *MLLT3*, *ITIH1*, *PSMB7*, *LGALS1*, *FXR2*, *GMDS*, *TSPAN8*, *PSMB1*, *GLUL*, *TSPAN1*, *MEST*, *ATP6V1C1*, *MPDU1*, *ATP6V1G1*, *HSPA5*, *IST1*, *TMEM192*, *NUTF2*, *DNAJC13*, *SLC9A3*, *ANO1*, *MYL6*, *EHD4*, *PTPRA*, *CAMK4*, *GPD1*, *TMBIM1*, *CPE*, *PDCD2*, *RPL26*, *SLC26A4*, *SHBG*	0.81138
	GOTERM_BP_DIRECT	GO:0051603	Proteolysis involved in cellular protein catabolic process4	0.00844	*PSMB7*, *HSPA5*, *PSMB1*, *PSMB10*	1.00000
	GOTERM_CC_DIRECT	GO:0005839	Proteasome core complex3	0.01096	*PSMB7*, *PSMB1*, *PSMB10*	0.81138
	GOTERM_CC_DIRECT	GO:0015030	Cajal body4	0.01261	*NOP58*, *CDK2*, *ZC3H8*, *WRAP53*	0.81138
	GOTERM_BP_DIRECT	GO:0045807	Positive regulation of endocytosis3	0.01837	*CALY*, *CBLL1*, *TSPAN1*	1.00000
	KEGG_PATHWAY	oas05231	Choline metabolism in cancer5	0.02594	*SLC44A5*, *DGKA*, *PLCG1*, *GPCPD1*, *HRAS*	1.00000
SD_G2 vs. CN_G2 (374 annotated genes)	GOTERM_CC_DIRECT	GO:0005737	Cytoplasm73	0.00000	*SLC23A2*, *GMEB2*, *BICDL1*, *STMN3*, *MSI1*, *OXT*, *ENDOV*, *XPO1*, *SIN3A*, *SOX15*, *POGZ*, *XPO5*, *TNFAIP8L1*, *POLH*, *KPNA1*, *DTX3L*, *ATP1B2*, *MYT1*, *RC3H2*, *COMMD4*, *ENAH*, *ASPM*, *IRF4*, *ALDH1A2*, *ZNF438*, *HCLS1*, *NSMF*, *NEIL1*, *TP53*, *KIZ*, *PLIN5*, *ITIH4*, *SPTBN5*, *PCNA*, *TUBGCP2*, *SATB2*, *ACTL7B*, *PXN*, *PSMB10*, *SOCS2*, *LIMA1*, *FBXO40*, *EPB41L5*, *PSMB7*, *PSMB4*, *NT5E*, *FXR2*, *DPP9*, *PSMB1*, *TSNAXIP1*, *GCN1*, *SCAI*, *GINS1*, *KLHDC3*, *ACD*, *NOP58*, *SRMS*, *CRADD*, *NEK6*, *MGA*, *MAD2L1BP*, *KLHL2*, *PTK6*, *PARP14*, *EIF2S2*, *GNMT*, *GDF9*, *CDAN1*, *MYBBP1A*, *TPP2*, *PTPN9*, *PDCD2*, *WRAP53*	0.00009
	GOTERM_CC_DIRECT	GO:0005839	Proteasome core complex4	0.00095	*PSMB7*, *PSMB4*, *PSMB1*, *PSMB10*	0.09601
	GOTERM_CC_DIRECT	GO:0070062	Extracellular exosome61	0.00149	*CAB39L*, *SNAP23*, *ARPC5L*, *SAT2*, *ZDHHC1*, *TXNDC17*, *MYDGF*, *FAM162A*, *UBXN6*, *CDK5RAP2*, *IQCB1*, *NUTF2*, *SLC9A3*, *ANO1*, *DNAJC5*, *GPD1*, *TMBIM1*, *RAB35*, *RPL26*, *ATP6V0D1*, *FBLN7*, *CRB2*, *ITIH4*, *ITIH3*, *SPTBN5*, *PCNA*, *ECHS1*, *RALB*, *ARL6*, *LCAT*, *ITIH1*, *SELENBP1*, *PSMB7*, *PSMB4*, *NT5E*, *FXR2*, *KIF3B*, *TSPAN8*, *GMDS*, *CREG1*, *PSMB1*, *MEST*, *TBC1D15*, *MPDU1*, *HSPA5*, *SPHKAP*, *TMEM192*, *UBE2G1*, *ASXL1*, *TPPP3*, *EHD4*, *LRG1*, *PTPRA*, *CAMK4*, *CPE*, *FNDC11*, *AGRP*, *PDCD2*, *SLC26A4*, *SHBG*, *LAMTOR3*	0.10010
	GOTERM_BP_DIRECT	GO:0051603	Proteolysis involved in cellular protein catabolic process5	0.00173	*PSMB7*, *PSMB4*, *HSPA5*, *PSMB1*, *PSMB10*	1.00000
	GOTERM_BP_DIRECT	GO:0030212	Hyaluronan metabolic process3	0.00463	*ITIH4*, *ITIH3*, *ITIH1*	1.00000
	GOTERM_MF_DIRECT	GO:0004298	Threonine-type endopeptidase activity4	0.00683	*PSMB7*, *PSMB4*, *PSMB1*, *PSMB10*	1.00000
	GOTERM_BP_DIRECT	GO:0045740	Positive regulation of DNA replication4	0.01049	*CTC1*, *PCNA*, *PLA2G1B*, *HRAS*	1.00000
	GOTERM_MF_DIRECT	GO:0043022	Ribosome binding4	0.01923	*SPCS1*, *HSPA5*, *EFL1*, *GCN1*	1.00000
	KEGG_PATHWAY	oas03050	Proteasome4	0.02103	*PSMB7*, *PSMB4*, *PSMB1*, *PSMB10*	1.00000
	KEGG_PATHWAY	oas00564	Glycerophospholipid metabolism5	0.04408	*PNPLA7*, *PLA2G1B*, *GPD1*, *LCAT*, *CDS2*	1.00000

## Discussion

The history of indigenous livestock and their physiological, anatomical and genetic responses to natural and artificial selection is at the core of their diversity (phenotypic and genetic) and resilience. Here, we present findings of the analysis of genomic variation in the indigenous African long-legged thin-tailed Desert Sheep from Sudan. The overall average H_*O*_ and H_*E*_ for the Sudanese thin-tailed Desert Sheep exceeded 0.300, suggesting high genetic variation. The values for the individual ecotypes are, however, similar to those reported in Barki sheep (Kim et al., 2016), an indigenous breed from a hot desert environment in Egypt, are higher than the values reported in Ethiopian sheep (Edea et al., 2017), but fall within the range reported in sheep raised by South African smallholder farmers (Molotsi et al., 2017) and in New Zealand breeds (Brito et al., 2017). The level of diversity in the four Chinese breeds analyzed here is similar to that of the Sudanese thin-tailed Desert Sheep in spite some of the breeds, such as the Tan Sheep and HZ having been exposed to artificial selection. Ascertainment bias and deliberate avoidance of inbreeding in the Chinese breeds could explain this result. The former should however be seen from the context that the Ovine Infinium^®^ HD SNP BeadChip carries assays for 606,006 loci with an average spacing of around 5 kb across the genome. These loci were selected from groups that differed in their MAF across 75 animals from an international panel of 34 domestic sheep breeds and two wild species of sheep (Bighorn and Thinhorn) (Anderson et al., 2014). The chip was also validated using 288 samples, generating 99% average call rates across SNPs and animals, and a call rate repeatability of 99.9978%.

The lack of stringent artificial selection coupled with random mating in the Sudanese thin-tailed Desert Sheep may explain their high levels of genetic diversity but low levels of genetic differentiation and inbreeding. The former may be enhancing their fitness and could be responsible for their adaptive resilience to the desert environments where they are raised.

We investigated demographic dynamics by assessing the trends in LD over genomic distances and N_*E*_ over the past 1,000 generations. All the samples analysed showed a rapid decay in LD within the first 300 kb. From around 0.1 Mb, the Chinese breeds had higher *r*^2^ values, which most likely reflected an attempt at their standardization as distinct breeds compared to the Sudanese thin-tailed Desert Sheep, where such efforts are lacking. However, for both subsets, the *r*^2^ values averaged below 0.15, suggesting very limited extension of high LD blocks across their genomes. This *r*^2^ value ranked below the minimum threshold range of 0.33 ≤ *r*^2^ ≤ 0.80 that is meaningful for GWAS (Ardlie et al., 2002; Carlson et al., 2004). Much denser marker coverage may thus be required for association analysis in the thin-tailed Desert Sheep and the Chinese breeds. Besides its importance for mapping traits, LD allows the estimation of N_*E*_ over generation time (Pritchard and Przeworski, 2001), acts as a significant genetic parameter for understanding population dynamics and can act as a measure of long-term performance of a population with regard to diversity and inbreeding (Fernández et al., 2005), and is useful for evaluating the risk status of populations/breeds (FAO, 1998; Duchev et al., 2006). A contraction in N_*E*_ from 270 and 500 generations ago was revealed in both the Sudanese thin-tailed Desert Sheep and the Chinese breeds, respectively. These contractions appeared not to have resulted in a concomitant reduction in genetic diversity. The contraction in the Chinese breeds may be associated with the start of their establishment as distinct breeds while that in the Sudanese thin-tailed Desert Sheep is difficult to explain. However, whole-genome sequence analysis has revealed declines in N_*E*_ in Ethiopian, Libyan and Sudanese sheep (Ahbara, 2019) and in Ethiopian goats based on the analysis of 50K SNP genotype data (Tarekegn et al., 2020). Mbole-Kariuki et al. (2014) also reported a bottleneck event in East African shorthorn Zebu cattle from western Kenya. The reduction in population sizes of the other African sheep and goat populations falls within the time period of the shrinkage in Sudanese thin-tailed Desert Sheep, while that of the East African shorthorn Zebu cattle started around 240 years ago. Historical fluctuations in climatic patterns resulting in cycles of favorable and deteriorating conditions in the continent (Verschuren, 2007) have been used to explain the fluctuations in N_*E*_.

The NJ phylogeny, PCA and ADMIXTURE allowed us to reveal the underlying genetic structure and divergence in the datasets analysed. We hypothesized that the ADMIXTURE pattern at *K* = 3, which was supported by NJ phylogeny and PCA, revealed an underlying broad-scale genetic structure as it showed (i) a separation of African sheep from the Chinese breeds, (ii) a separation of fat-tailed sheep (Tan breed) from both African and Chinese thin-tailed sheep, and (iii) different genetic backgrounds in both the African and the Chinese thin-tailed sheep. While the first result suggest genetic divergence that has most likely arisen due to reproductive isolation between sheep in different continents coupled with genetic drift, the second result confirm the existence of differences in genetic makeup of fat-tailed and thin-tailed sheep. A similar result based on the analysis of microsatellites was reported previously between African fat-tailed and thin-tailed sheep (Muigai, 2003). Furthermore, the divergence of the fat-tailed Tan Sheep from the other Chinese breeds can be due to artificial selection for lamb pelt and lustrous white curly fleece in the Tan sheep. The clear divergence between the Sudanese thin-tailed Desert Sheep and the Chinese thin-tailed sheep suggest at least two possibilities: (i) the domestication of at least two autosomal gene pools of thin-tailed sheep in the Fertile Crescent and their subsequent independent dispersal westwards to Africa and eastwards to China, and (ii) the dispersal of one gene pool followed by genetic divergence driven by genetic drift due to reproductive isolation and natural selection driving adaptation to low altitude hot arid environments in Africa or high altitude alpine arid environments in China. Although mitogenome analysis has identified two waves of sheep migration comprising three maternal lineages across eastern Eurasian (Lv et al., 2015), the study did not include sheep from Africa and therefore the first scenario is difficult to ascertain. We therefore favor the second scenario given that recent analysis of autosomal genomic data has provided compelling evidence for climate-mediated selection pressure driving genetic divergence in sheep (Lv et al., 2014) while differences in precipitation affecting feed availability has also been shown to drive variation in natural selection at a global scale (Siepielski et al., 2017).

A combination of NJ phylogeny, PCA and ADMIXTURE (*K* > 5) results also revealed what we hypothesized to be a fine-scale genetic structure among the individuals analyzed. The analyses revealed at least two distinct genetic clusters in the Sudanese thin-tailed Desert Sheep; one which was specific to AL and another to the four remaining ecotypes. The analyses also identified two genetic clusters in the Sudanese thin-tailed sheep from China: one cluster was exclusive to HZ and another occurred in QOL and ZK and at low frequency in HZ. Given that the five ecotypes of thin-tailed Desert Sheep are all derived from a low altitude hot arid environment while the Chinese thin-tailed breeds are adapted to an alpine/sub-alpine high-altitude rangeland, this fine-scale genetic structure was unexpected. It may hint at a complex genome architecture in the thin-tailed sheep because it cannot be explained by differential selection for adaptation. It is, however, likely that artificial selection may be driving the divergence in the Chinese thin-tailed sheep. With a common genomic background observed in QOL and ZK and the same occurring at a low frequency in HZ, it suggests that HZ has been gradually diverging from QOL and ZK. HZ has a predominantly black coat while QOL and ZK have mainly white coats with some black to brown patches around heads and legs. QOL and ZK also occur in close geographic proximity and can thus cross mate while HZ is isolated and farmers keeping this breed avoid mating it with other breeds, so as to maintain its distinct black coat, genetic purity and recognition.

We investigated molecular signatures of selection to gain insights on the divergence of SD_G1 and SD_G2 genetic clusters of the Sudanese thin-tailed Desert Sheep. For this reason, we used three approaches and contrasted SD_G1 and SD_G2 with each other and with two of the genetic clusters: CN_G1 and CN_G2 observed in Chinese sheep. We used the ROH analysis to provide evidence for selection within a cluster. If such signatures overlapped with those identified by *F*_*ST*_ and/or XP-EHH and were observed in at least two comparative analyses, then we took this to be a reliable evidence of positive selection in the specific genetic cluster. We therefore limited our discussion to the putative genes found within the candidate regions that overlapped between at least two approaches and in more than two comparative analyses involving the two genetic groups observed in the Sudanese thin-tailed Desert Sheep (Tables 2, 3).

**FIGURE 7:**
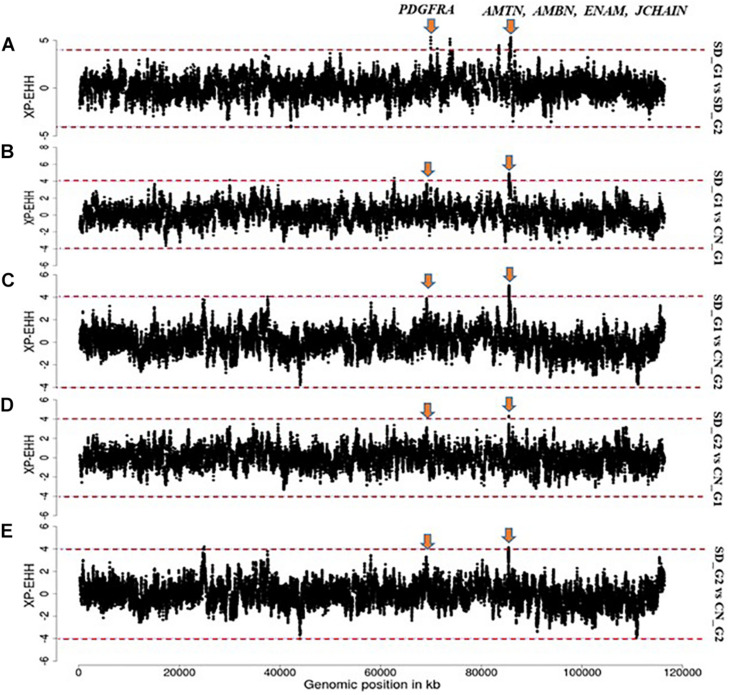
Manhattan plots showing the strongest candidate signatures of selection as determined with *XP-EHH* on OAR6. **(A)** SD_G1 vs. SD_G2; **(B)** SD_G1 vs. CN_G1; **(C)** SD_G1 vs. CN_G2; **(D)** SD_G2 vs. CN_G1; **(E)** SD_G2 vs. CN_G2.

**FIGURE 8:**
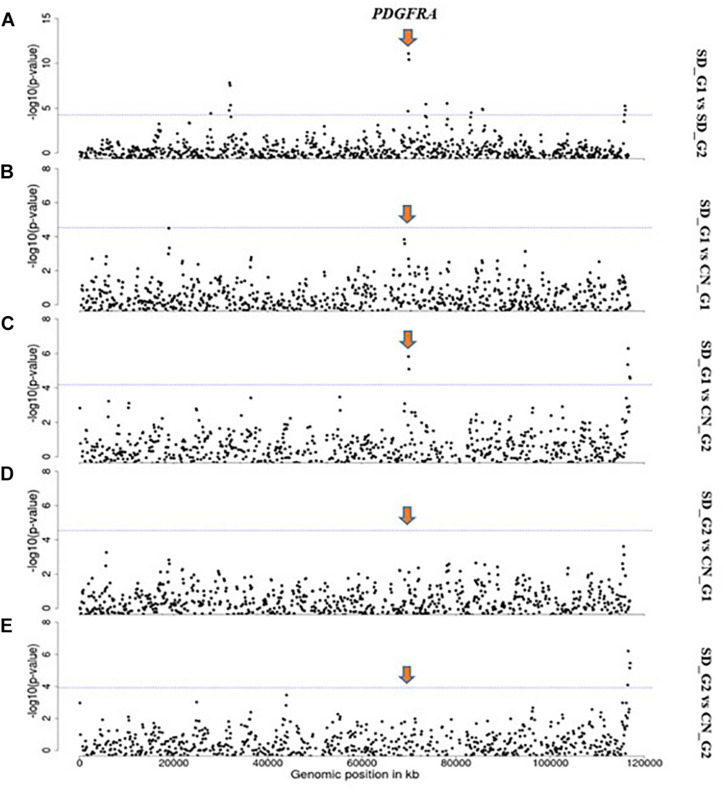
Manhattan plots showing the strongest candidate signatures of selection as determined with *F*_*ST*_ on OAR6. **(A)** SD_G1 vs. SD_G2; **(B)** SD_G1 vs. CN_G1; **(C)** SD_G1 vs. CN_G2; **(D)** SD_G2 vs. CN_G1; **(E)** SD_G2 vs. CN_G2.

Based on our criteria, nine candidate regions that overlapped between at least two approaches and were observed in at least two comparative analyses were identified in SD_G1. These candidate regions were located on OAR3, 5, 6 (two regions), 10, 13, 18 (two regions), and 26 (one region) (Table 2). Within these candidate regions, we found genes that are associated with functions relating to adaptation to abiotic and biotic factors and morphology. For instance, the most significant region on OAR6 (Figures 7, 8) spanned six genes, one of which was *AMBN* (ameloblastin), a candidate gene for gastrointestinal nematode resistance in sheep (Atlija et al., 2016; Berton et al., 2017). The *PDGFRA* gene occurred in another candidate region on OAR6 (Figures 7, 8). It has been reported to be a key gene in cytokine signaling and was observed to be amongst genes in biological pathways that are involved in host immune responses against parasitic infections (Benavides et al., 2015). *PDGFRA* has also been reported to be tightly associated with the dominant white coat color in the pig (Johansson et al., 1992; Moller et al., 1996). Nazari-Ghadikolaei et al. (2018) also found significant association between *PDGFRA* and *KIT* with white coat color in Markhoz goat from Iran. The white coat color predominated in AL based on field observations made by the first author during sampling. *PDGFRA* has also been linked to fat deposition in Libyan long fat-tailed sheep (Mastrangelo et al., 2019) while *CAMK4* found on OAR5 has been associated with tail morphology and fat deposition in sheep (Moradi et al., 2012; Ma et al., 2018; Ahbara et al., 2019; Li et al., 2020). These results likely suggest that divergent selection for parasite resistance, coat color, differential fat deposition and tail morphology may explain the divergence of SD_G1. With the lack of phenotypic data, our suggestions would need to be confirmed with more detailed studies that include an assessment of appropriate phenotypes.

Our criteria also revealed four candidate regions that overlapped between at least two approaches and in at least two comparative analyses in SD_G2 (Table 3). These regions were observed on OAR3 (three regions) and OAR8 (one region). The region on OAR8 was the strongest and it spanned three genes, *FAM120B*, *PSMB1*, and *PDCD2*. *FAM120B* has been suggested to be a potential candidate for twinning rate in humans (Painter et al., 2010) and in lowly ovulating mammals (Vinet et al., 2012). The *PSMB1*, which was the top candidate gene at this region, has been associated with functional adaptation of the transcriptome to mastitis-causing pathogens in mammary gland of livestock (Loor et al., 2011). The *SOCS2*, which was found in one of the candidate regions on OAR3, has been linked with body weight and milk production, and an increased susceptibility to inflammation of mammary glands (Rupp et al., 2015). It is important to take note that most pastoral societies in Africa prefer larger sized animals with higher milk production as offspring from such animals are thought to thrive better in hot arid environments. This indirect preference for such individuals may be responsible for this selection signal. *SOCS2* has also been reported to play important roles in key adaptive phenotypes in sheep, including general immune response following infection (Yang et al., 2015; Al Kalaldeh et al., 2019) and resistance to gastrointestinal nematode (*Haemonchus contortus*) (Estrada-Reyes et al., 2019). The proteasome *PSMB7* and the heat shock protein *HSPA5*, both found in the region on OAR3, were reported to be upregulated during blastocyst implantation in hamsters (Lei et al., 2014). These results suggest that the divergence of SD_G2 could be associated with differential resistance to bacterial infections, productive and reproductive performance. However, as for SD_G1, this finding will need to be investigated further with much detailed analyses of individuals with relevant phenotypes.

**FIGURE 9:**
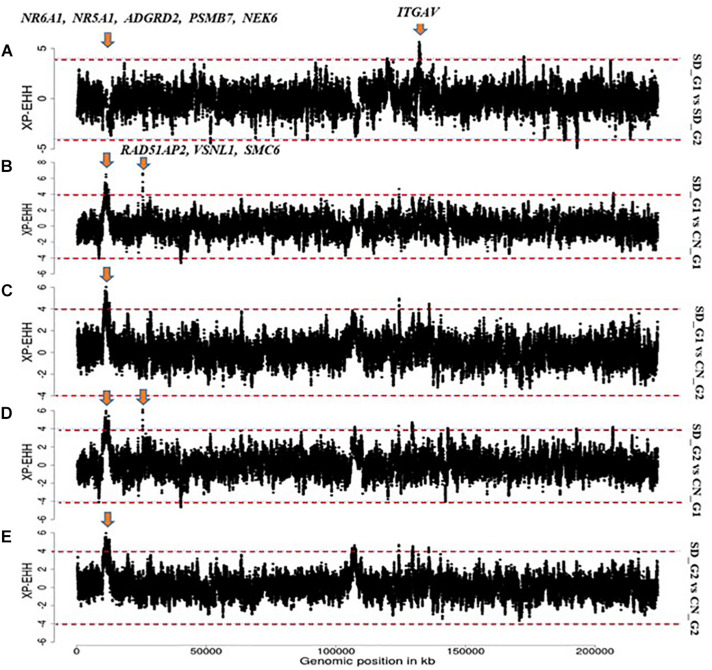
Manhattan plots showing the strongest candidate signatures of selection as determined with *XP-EHH* on OAR3. **(A)** SD_G1 vs. SD_G2; **(B)** SD_G1 vs. CN_G1; **(C)** SD_G1 vs. CN_G2; **(D)** SD_G2 vs. CN_G1; **(E)** SD_G2 vs. CN_G2.

The candidate region on OAR3 (10,700,001–11,800,000 bp) differentiated both SD_G1 (Table 2) and SD_G2 (Table 3) from both CN_G1 and CN_G2 (Figure 9) and was therefore of interest. The top significant window at this region spanned five genes (*NR6A1*, *NR5A1*, *ADGRD2*, *PSMB7*, and *NEK6*) and the top-most significant position was close to *NR5A1*. The expression of *NR5A1* drives Leydig cell differentiation from progenitor cells (Barsoum and Yao, 2010), thus initiating steroidogenesis (Martin and Tremblay, 2010). In mice, *NR5A1* has been shown to be essential in the formation of pituitary, gonad and adrenal glands (Luo et al., 1994). Another region that differentiated SD_G1 and SD_G2 from both CN_G1 and CN_G2 occurred on OAR14. Two genes were present in this region, one of which was *AP1G1*. Proteomic analysis of sheep uterus has revealed the role of *AP1G1* in prolificacy (La et al., 2020). Whether the function of *NR5A1* and *AP1G1* suggested inherent genetic differences in fertility and prolificacy between the Sudanese thin-tailed Desert Sheep and the Chinese breeds is difficult to say in the absence of appropriate phenotype data. Strong evidence suggests that *NR6A1* is a strong candidate gene underlying vertebrae number in domestic pigs (Mikawa et al., 2007; Rubin et al., 2012) and the number of thoracic vertebrae in domestic sheep (Li et al., 2019). From a phenotypic perspective, this result is interesting as it suggests that the Sudanese thin-tailed Desert Sheep can be differentiated from the Chinese thin-tailed sheep based on the number of vertebrae. Indeed, the tail of Sudanese sheep consists of 23 caudal vertebrae (Ahbara, 2019), while that of Chinese sheep comprises less than 18 (Zhi et al., 2018).

The African long-legged thin-tailed sheep is raised in desert environments where they are exposed year-round, to complex interacting biophysical stressors, including high temperatures, physical exhaustion, direct solar radiation, feed and water scarcity. In this context, the revelation of the GO biological term “hyaluronan metabolic process (GO:0030212)” in four out of the six comparisons involving SD_G1 and SD_G2 is relevant. Hyaluronan (HA) comprises a major component of the extracellular matrix (ECM) in vertebrates and is a straight chain glycosaminoglycan which mediates diverse functions depending on molecular size and tissue concentration, both of which are regulated by the balance between its biosynthesis and catabolism (Hascall and Esko, 2015). HA occurs virtually in all vertebrate tissues and fluids, but skin, the first defense against environmental insults, is its largest reservoir containing more than 50% of the total body HA. The HA content of dermis is far greater than that of epidermis, and accounts for most of the 50% of the total body HA in skin. HA has excellent water retention ability and remarkable tissue hydration capacity, and at high concentrations, as found in the ECM of dermis and epidermis, it regulates water balance and osmotic pressure, functions as an ion exchange resin and regulates ion flow (Stern, 2010). HA also functions in the reepithelization process due to several of its properties, including being an integral part of the ECM of basal keratinocytes, the major constituent of epidermis, its free-radical scavenging function and its role in keratinocyte proliferation and migration (Tammi et al., 1989). It has been observed that the content of HA increases in the presence of retinoic acid (vitamin A) (Tammi et al., 1989) and the effects of retinoic acid against UV-induced skin damage may be correlated, at least in part, with an increase in skin HA content, giving rise to increased tissue hydration. It has been suggested that the free-radical scavenging property of HA contributes to protection against repeated exposure to the sun’s UV radiation (Tuhkanen et al., 1998; Averbeck et al., 2007). The rapid turnover of HA in tissues suggests tightly controlled modes for modulating steady state levels of HA and this is important because rapid increases are required in situations of extreme stress (Kobayashi et al., 2020). Therefore, the ability to provide immediate high HA levels acts as a survival mechanism for vertebrates and may explain the rapid rates of HA turnover under basal conditions. Furthermore, HA plays a role in innate immunity. Although it binds to CD44, there is evidence showing that HA degradation products transduce inflammatory signal through toll-like receptor 2 (*TLR2*), *TLR4* or both in macrophages and dendritic cells. Thus, the HA metabolic process may be facilitating the adaptation to desert environments in African long-legged thin tailed sheep.

## Conclusion

In this study, we analyzed the genetic diversity, structure and signatures of selection in the African thin-tailed Desert Sheep sampled from Sudan. We included one breed of fat-tailed and three breeds of thin-tailed sheep from China for comparative genomic analysis. We found high levels of genetic diversity but low levels of genetic differentiation among the five ecotypes of Sudanese thin-tailed Desert Sheep. The analysis also revealed a broad- and fine-scale genetic structures in the sheep analyzed, suggesting that these would need to be accounted for in genome-wide association analysis in the discovery of the genetic basis of important traits and in breeding program design. Selection signature analysis identified candidate regions that could potentially differentiate the two genetic clusters observed in the African thin-tailed Desert Sheep from the two genetic clusters observed in the Chinese thin-tailed sheep. These regions spanned a set of potential candidate genes associated with traits of adaptive, production and reproduction significance as well as morphological differentiation. While our study provides a foundation for understanding the genome structure and dynamics of African indigenous sheep, it reveals findings that could form the basis of studies that combine genomic and phenomic approaches in the quest to understand the genome architecture of indigenous livestock.

## Data Availability Statement

The data presented in the study are deposited in the figshare repository, accession number doi: 10.6084/m9.figshare.14785278.v1.

## Ethics Statement

The blood samples from the Sudanese thin-tailed Desert Sheep were collected after consent was granted by flock owners and local administration officials in Sudan. No further permissions were required from the ethics committee of the Organization of Veterinary Service, Government of Sudan. All animal experiments in this study were fully approved by the Animal Care and Use Committee of the Institute of Animal Sciences, Chinese Academy of Agricultural Sciences (IAS-CAAS) with the following reference number: IASCAAS-AE-03, on September 1, 2014.

## Author Contributions

QZ, YM, and JM conceived, designed, and supervised the study and genotyped the Chinese breeds. JM, MR, and AH genotyped the Sudanese thin-tailed Desert Sheep. FE organised the sampling in Sudan. AAh and AAb analyzed the data with support from HB, RI and LX. AAb, AAh, J-LH, and JM wrote and revised the manuscript. All authors read and approved the final version.

## Conflict of Interest

The authors declare that the research was conducted in the absence of any commercial or financial relationships that could be construed as a potential conflict of interest.

## Supplementary Material

Supplementary Figure 1 Z-transformed average fixation index (Z*F*_*ST*_) for autosomal 200 kb windows.

Supplementary Figure 2 Normalized distribution of XP-EHH scores.

Supplementary Table 1 The sampling locations of the thin-tailed Desert Sheep ecotypes from Sudan and the breeds from China that were used in the current study.

Supplementary Table 2 Phenotypic characteristics of the thin-tailed Desert Sheep ecotypes and the climatic characteristics of their production environment.

Supplementary Table 3 Pairwise genetic differentiation (*F*_*ST*_) among the five ecotypes of Sudanese thin-tailed Desert Sheep and four breeds of sheep from China herein analyzed.

Supplementary Table 4 The candidate regions spanning genes within the SD_G1 sheep group identified through the analysis of ROH islands.

Supplementary Table 5 The candidate regions spanning genes within the SD_G2 sheep group identified through the analysis of ROH islands.

Supplementary Table 6 The candidate regions spanning genes within the SD_G1 (SD_G1 vs. SD_G2, CN_G1, and CN_G2) sheep group identified by XP-EHH.

Supplementary Table 7 The candidate regions spanning genes within the SD_G2 (SD_G2 vs. SD_G1, CN_G1, and CN_G2) sheep group identified by XP-EHH.

Supplementary Tables 8 The candidate regions spanning genes within the Sudanese sheep groups (SD_G1 vs. SD_G2) identified by *F*_*ST*_.

Supplementary Table 9 The candidate regions spanning genes within the SD_G1 (SD_G1 vs. CN_G1 and CN_G2) sheep group identified by *F*_*ST*_.

Supplementary Table 10 The candidate regions spanning genes within the SD_G2 (SD_G2 vs. CN_G1 and CN_G2) sheep group identified by *F*_*ST*_.
