# Supplementary material for: Genome Divergence and Dynamics in the Thin-Tailed Desert Sheep From Sudan
Source: Front Genet. 2021 Jul 19;12:659507. doi: 10.3389/fgene.2021.659507 (PMC8327097; doi:10.3389/fgene.2021.659507)
Supplement: Supplementary Figure 1 — Z-transformed average fixation index (ZFST) for autosomal 200 kb windows. [file Data_Sheet_1.docx]

Supplementary Materials

**Supplementary Figures**

**
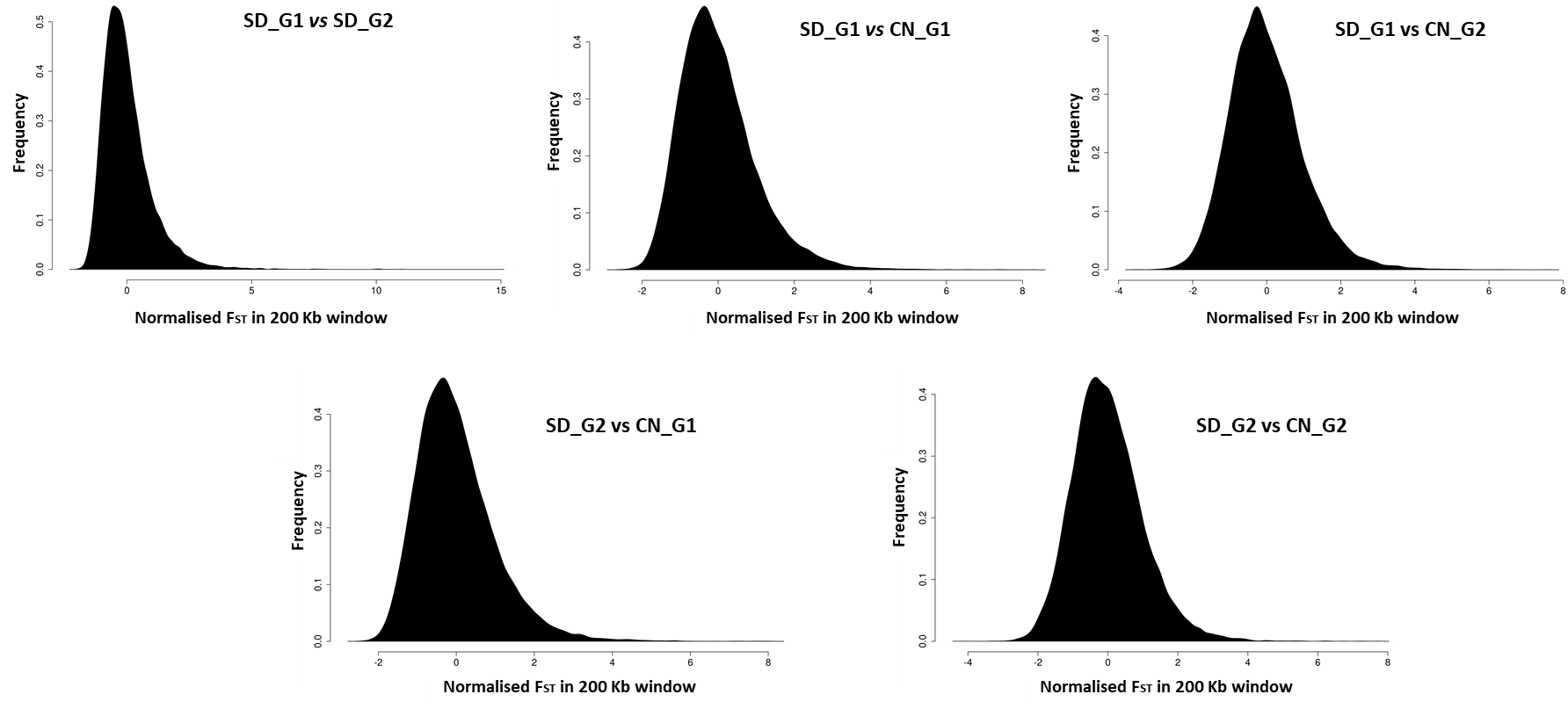
**

Supplementary Figure S1 Z-transformed average fixation index (ZF_st_) for autosomal 200-kb windows


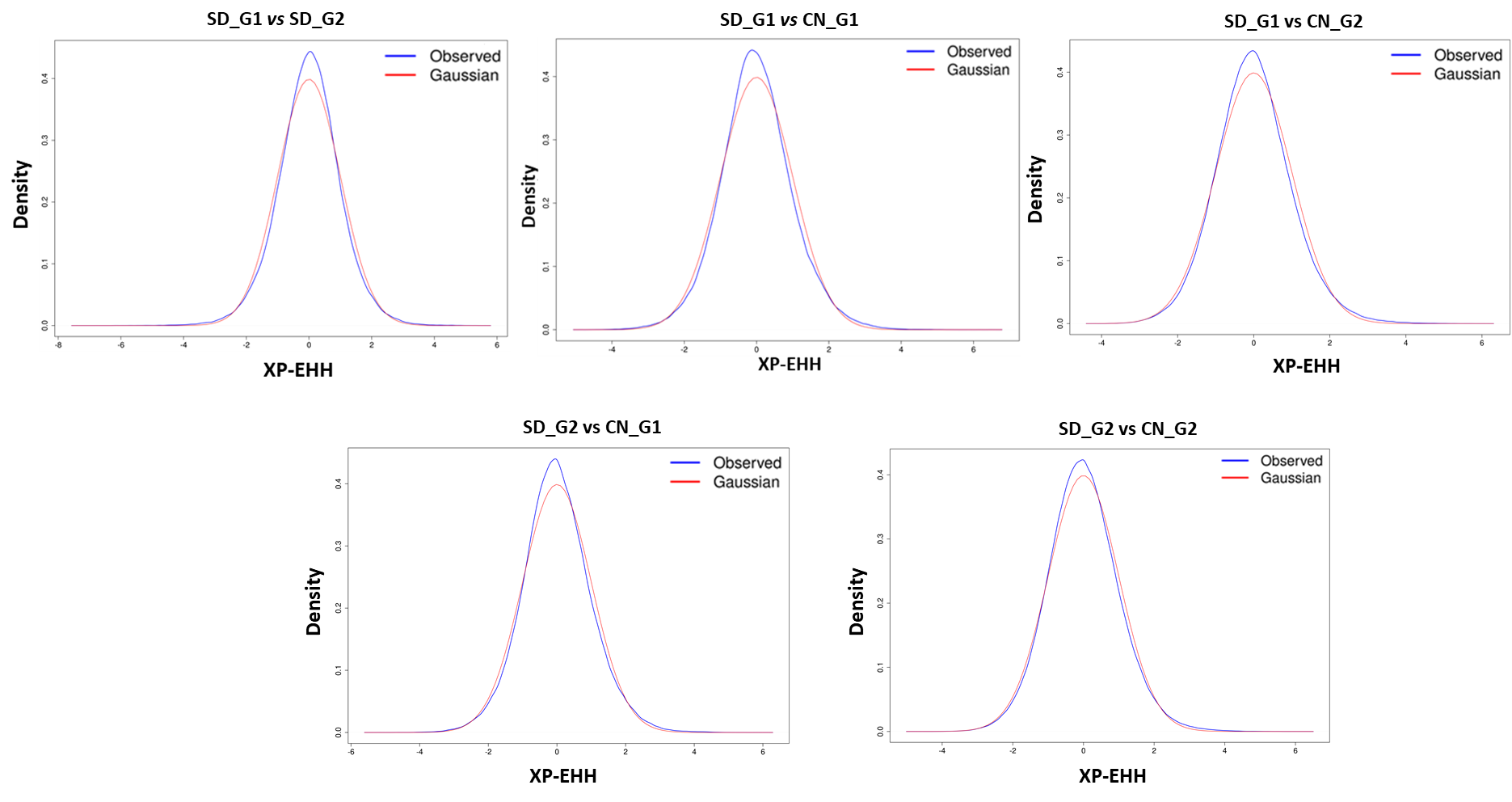


**Supplementary Figure S2** Normalized distribution of XP-EHH scores.

**Supplementary Tables**

Supplementary Table S1. The thin-tailed Desert Sheep Ecotypes from Sudan and the breeds from China that were used in the current study and their sampling locations

| **Country** | **Breed/Population** | **Code** | **Zone** | **Sample size** | **Latitude (N)** | **Longitude (E)** | **Altitude (meters)** | **Ecology** |
| --- | --- | --- | --- | --- | --- | --- | --- | --- |
| Sudan | Al Ahamda | AL | North Kordofan (Al-Rahad) | 25 | 12^O^ 43’ | 30^O^ 39’ | 506 | Lowland |
|  | Buzee | BU | North Kordofan (Umm-Ruwaba) | 23 | 12^O^ 54’ | 31^O^ 13’ | 457 | Lowland |
|  | Hammari | HA | West Kordofan (El-Khwai) | 24 | 13^O^ 09’ | 29^O^ 2’ | 519 | Lowland |
|  | Kabashi | KA | North Kordofan (Sowdari) | 25 | 15^O^ 14’ | 28^O^ 43’ | 517 | Lowland |
|  | Shanbali | SH | White Nile (Tendelti) | 24 | 13^O^ 1’ | 31^O^ 52’ | 422 | Lowland |
| China | Black Tibetan | HZ | Qinghai – Alpine frigid | 15 | 35^O^ 9’ | 101^O^ 23’ | 3400 | Highland |
|  | Oula | QOL | Qinghai – Alpine frigid | 15 | 34^O^ 30’ | 101^O^ 34’ | 3400 | Highland |
|  | Tan | TS | Ningxia Province | 18 | 38^O^ 29’ | 14^O^ 57’ | 1109 | Lowland |
|  | Zeku | ZK | Qinghai – Alpine frigid | 15 | 35^O^ 39’ | 101^O^ 24’ | 3400 | Highland |

Supplementary Table S2 Phenotypic characteristics of the thin-tailed Desert Sheep ecotypes and the climatic characteristics of their production environment

| **Phenotypic and environmental variables** | **Populations** | | | | |
| --- | --- | --- | --- | --- | --- |
|  | **Al Ahamda** | **Buzee** | **Hammari** | **Kabashi** | **Shanbali** |
| Coat Color | Mostly White with pigmentation | Red brown with White scrotum | Dark Brown | White with Red or Black pigmentation | Brown with White scrotum |
| Body Condition | Medium | Satisfactory to fat | Satisfactory to fat | Satisfactory | Satisfactory to fat |
| Uses | Meat/milk | Meat/milk | Meat/milk | Meat/milk | Meat/milk |
| Ears Orientations | Lateral | Drooping | Drooping | Drooping | Lateral |
| Nose Profile | Flat | Convex | Convex | Convex | Convex |
| Type Tail | Long thin-tail | Long thin-tail | Long thin-tail | Long thin-tail | Long thin-tail |
| Horn Orientation | Polled | Forward in males | Few males with little horns | Forward in males | Polled |
| Community | Al-Ahamda | Buzaa | Hammer | Kababish | Shanabla/Gwamaa |
| Temperature (^o^C) | 30 - 40 | 30-40 | 30-40 | 35-40 | 30-40 |
| Mean rain fall (mm) | 410 | 353 | 370 | 209 | 300 |
| Management | Pastoral/agro-pastoral systems | | | | |

Source: El-hag, FM., Fadlalla B., Mukhtar HK. (2001). Some Production Characteristics of Sudan Desert Sheep under Range Conditions in North Kordofan, Sudan. *Tropical Animal Health and Production* **33**, 229–239.

**Supplementary Table S3.** Pairwise genetic differentiation (F_ST_) among the five ecotypes of thin-tailed Desert Sheep and four breeds of sheep from China herein analysed

| Population | AL | BU | HA | KA | SH | HZ | QOL | TS | ZK |
| --- | --- | --- | --- | --- | --- | --- | --- | --- | --- |
| Al-Ahamda | * |  |  |  |  |  |  |  |  |
| Buzee | 0.009 | * |  |  |  |  |  |  |  |
| Hammari | 0.008 | 0.003 | * |  |  |  |  |  |  |
| Kabashi | 0.011 | 0.006 | 0.004 | * |  |  |  |  |  |
| Shanbali | 0.009 | 0.003 | 0.004 | 0.006 | * |  |  |  |  |
| Black Tibetan | 0.099 | 0.099 | 0.097 | 0.010 | 0.010 | * |  |  |  |
| Oula | 0.066 | 0.065 | 0.064 | 0.066 | 0.065 | 0.070 | * |  |  |
| Zeku | 0.067 | 0.066 | 0.065 | 0.068 | 0.066 | 0.070 | 0.011 | 0.11 | * |
| Tan | 0.108 | 0.107 | 0.108 | 0.108 | 0.107 | 0.138 | 0.108 | * |  |

**Supplementary Table S4.** The candidate regions spanning genes within the SD_G1 sheep group identified *via* ROH islands.

| Reg. | Chr. | Start | Stop | Size (Mb) | No of  Genes | Genes |
| --- | --- | --- | --- | --- | --- | --- |
| 1 | 1 | 25525099 | 25681293 | 0.156 | 2 | *TTC39A, EPS15* |
| 2 | 1 | 37621965 | 37636235 | 0.014 | 1 | *DOCK7* |
| 3 | 1 | 111964300 | 112078930 | 0.115 | 1 | *SH2D1B* |
| 4 | 1 | 119571896 | 119644393 | 0.072 | 1 | *MRPS6* |
| 5 | 1 | 199686332 | 199724053 | 0.038 | 1 | *IGF2BP2* |
| 6 | 1 | 215916258 | 216083412 | 0.167 | 1 | *ENSOARG00000014783* |
| 7 | 1 | 223073196 | 223225202 | 0.152 | 1 | *ENSOARG00000023528* |
| 8 | 1 | 227300609 | 227413450 | 0.113 | 1 | *ENSOARG00000002537* |
| 9 | 1 | 266692268 | 266761527 | 0.069 | 1 | *CHAF1B* |
| 10 | 1 | 106050370 | 106283918 | 0.234 | 2 | *ENSOARG00000006782, ENSOARG00000006800* |
| 11 | 1 | 163605359 | 163714699 | 0.109 | 2 | *ENSOARG00000018109, ABI3BP* |
| 12 | 1 | 223402260 | 223628019 | 0.226 | 2 | *ENSOARG00000025614, U6* |
| 13 | 1 | 252663891 | 252805582 | 0.142 | 2 | *ENSOARG00000025633, KY* |
| 14 | 1 | 275307838 | 275415508 | 0.108 | 2 | *PP2D1, KAT2B* |
| 15 | 1 | 109703706 | 109957419 | 0.254 | 3 | *VANGL2, ENSOARG00000008861, CD84* |
| 16 | 1 | 128991659 | 129127059 | 0.135 | 3 | *MRPL39, ENSOARG00000022750, U6* |
| 17 | 1 | 154631478 | 154761124 | 0.130 | 3 | *CGGBP1, ZNF654, C3orf38* |
| 18 | 1 | 175393900 | 175503536 | 0.110 | 3 | *SLC9C1, ENSOARG00000019295, CD200* |
| 19 | 1 | 68580173 | 68776536 | 0.196 | 5 | *BTBD8, U6, C1orf146, GLMN, RPAP2* |
| 20 | 1 | 185146440 | 185292803 | 0.146 | 5 | *KPNA1, U6, PARP9, DTX3L, PARP14* |
| 21 | 1 | 255103211 | 255363529 | 0.260 | 5 | *ACAD11, ACKR4, DNAJC13, ENSOARG00000009070, ACP3* |
| 22 | 2 | 9128447 | 9157161 | 0.029 | 1 | *ATP6V1G1* |
| 23 | 2 | 14616917 | 14797915 | 0.181 | 1 | *U6* |
| 24 | 2 | 51119473 | 51298541 | 0.179 | 1 | *ZCCHC7* |
| 25 | 2 | 114004099 | 114213605 | 0.210 | 1 | *ENSOARG00000025798* |
| 26 | 2 | 137449382 | 137501529 | 0.052 | 1 | *TLK1* |
| 27 | 2 | 177930202 | 178094018 | 0.164 | 1 | *ACTR3* |
| 28 | 2 | 184124267 | 184261367 | 0.137 | 1 | *PTPN4, EPB41L5* |
| 29 | 2 | 209555522 | 209682744 | 0.127 | 1 | *ENSOARG00000025845* |
| 30 | 2 | 219621657 | 219649686 | 0.028 | 1 | *STK36* |
| 31 | 2 | 224038745 | 224077728 | 0.039 | 1 | *ACSL3* |
| 32 | 2 | 232780941 | 233033309 | 0.252 | 1 | *DIS3L2* |
| 33 | 2 | 39220967 | 39364679 | 0.144 | 2 | *U6, EBF2* |
| 34 | 2 | 159646847 | 159754039 | 0.107 | 2 | *EPC2, ENSOARG00000023938* |
| 35 | 2 | 232457567 | 232530051 | 0.072 | 2 | *ENSOARG00000020723, PDE6D* |
| 36 | 2 | 14342189 | 14593219 | 0.251 | 4 | *ELP1, ENSOARG00000007143, ACTL7B, ENSOARG00000007153* |
| 37 | 2 | 113009833 | 113113775 | 0.104 | 4 | *TUBGCP5, ENSOARG00000015810, IMP4, PTPN18* |
| 38 | 2 | 234400498 | 234568768 | 0.168 | 10 | *ENSOARG00000000244, ENSOARG00000000261, ENSOARG00000000281, DCDC2B, IQCC, TXLNA, KPNA6, U6, ENSOARG00000022172, TMEM39B* |
| 39 | 3 | 12404574 | 12497707 | 0.093 | 1 | *U6* |
| 40 | 3 | 19658029 | 19658029 | 0.000 | 1 | *NOL10* |
| 41 | 3 | 63368153 | 63561221 | 0.193 | 1 | *BCL11A* |
| 42 | 3 | 106926661 | 107067960 | 0.141 | 1 | *ENSOARG00000024141* |
| 43 | 3 | 124303498 | 124460134 | 0.157 | 1 | *CEP290, TMTC3* |
| 44 | 3 | 129702880 | 129818498 | 0.116 | 1 | *SOCS2* |
| 45 | 3 | 28267506 | 28384405 | 0.117 | 2 | *U4, HS1BP3* |
| 46 | 3 | 28418373 | 28583449 | 0.165 | 2 | *LDAH, U6* |
| 47 | 3 | 46641216 | 46760579 | 0.119 | 2 | *USP34, ENSOARG00000020279* |
| 48 | 3 | 153813836 | 153863913 | 0.050 | 2 | *ENSOARG00000002929, ENSOARG00000023603* |
| 49 | 3 | 154389895 | 154459355 | 0.069 | 2 | *MSRB3, LEMD3* |
| 50 | 3 | 206745061 | 206865854 | 0.121 | 4 | *ENSOARG00000003251, ENSOARG00000003403, ENSOARG00000003494, ENSOARG00000003615* |
| 51 | 3 | 213802419 | 213938323 | 0.136 | 4 | *PICK1, SLC16A8, BAIAP2L2, PLA2G6* |
| 52 | 3 | 132296009 | 132344359 | 0.048 | 5 | *ENSOARG00000026026, HOXC4, HOXC5, ENSOARG00000022473, HOXC6* |
| 53 | 3 | 10727840 | 11494280 | 0.766 | 17 | *RABEPK, PPP6C, SCAI, ENSOARG00000025028, GOLGA1, ARPC5L, ENSOARG00000013275, WDR38, U6, OLFML2A, ENSOARG00000023155, oar-mir-181a-2, NR6A1, NR5A1, ADGRD2, PSMB7, NEK6* |
| 54 | 4 | 36550371 | 36558742 | 0.008 | 1 | *SEMA3A* |
| 55 | 4 | 46204059 | 46281773 | 0.078 | 1 | *KMT2E* |
| 56 | 4 | 69958575 | 70023831 | 0.065 | 1 | *ENSOARG00000025241* |
| 57 | 4 | 78374607 | 78600914 | 0.226 | 1 | *ENSOARG00000008562* |
| 58 | 4 | 78998167 | 79050093 | 0.052 | 1 | *GLI3* |
| 59 | 4 | 86802569 | 86937909 | 0.135 | 1 | *CADPS2* |
| 60 | 4 | 1879559 | 1881006 | 0.001 | 2 | *ENSOARG00000025204, ENSOARG00000025205* |
| 61 | 4 | 101473029 | 102073758 | 0.601 | 3 | *CREB3L2, AKR1D1, TRIM24* |
| 62 | 4 | 48591047 | 48726509 | 0.135 | 5 | *COG5, DUS4L, BCAP29, ENSOARG00000024106, SLC26A4* |
| 63 | 4 | 103745749 | 103933590 | 0.188 | 5 | *ENSOARG00000025257, ADCK2, NDUFB2, BRAF, 5S_rRNA* |
| 64 | 5 | 49139142 | 49171418 | 0.032 | 5 | *ENSOARG00000018230, CD14, TMCO6, NDUFA2, IK* |
| 65 | 5 | 49589412 | 49704569 | 0.115 | 5 | *PCDHB6, PCDHB7, ENSOARG00000013913, ENSOARG00000018839, PCDHB14* |
| 66 | 5 | 48997231 | 49137648 | 0.140 | 8 | *ENSOARG00000017846, 5S_rRNA, U6, SRA1, APBB3, ENSOARG00000018183, SLC35A4, ENSOARG00000018230* |
| 67 | 6 | 36350660 | 36475646 | 0.125 | 1 | *PPM1K* |
| 68 | 6 | 68912541 | 69107989 | 0.195 | 2 | *ENSOARG00000018767, LNX1* |
| 69 | 6 | 24691924 | 24877240 | 0.185 | 4 | *ENSOARG00000013905, ENSOARG00000023729, H2AZ1, DNAJB14* |
| 70 | 6 | 36179174 | 36267547 | 0.088 | 4 | *ENSOARG00000000388, ENSOARG00000000447, HERC5, ENSOARG00000001138* |
| 71 | 6 | 116051931 | 116125336 | 0.073 | 4 | *ENSOARG00000015218, NSD2, LETM1, FGFR3* |
| 72 | 6 | 37236177 | 37621027 | 0.385 | 6 | *FAM184B, NCAPG, DCAF16, ENSOARG00000000478, ENSOARG00000004249, ENSOARG00000004333* |
| 73 | 6 | 69700001 | 70000000 | 0.300 | 0 | *PDGFRA, ENSOARG00000021645* |
| 74 | 6 | 85447324 | 85695088 | 0.248 | 6 | *ENSOARG00000011228, ENSOARG00000008596, AMTN, AMBN, ENAM, JCHAIN* |
| 75 | 6 | 116441474 | 116726342 | 0.285 | 9 | *ENSOARG00000016260, ENSOARG00000016268, ENSOARG00000016282, GAK, CPLX1, ENSOARG00000016536, ENSOARG00000026630, ENSOARG00000026631, ENSOARG00000026632* |
| 76 | 6 | 116791619 | 116991348 | 0.200 | 12 | *ENSOARG00000008606, MAEA, ENSOARG00000016735, ENSOARG00000016756, SLC49A3, PDE6B, PIGG, ENSOARG00000026634, ENSOARG00000026635, ENSOARG00000026636, ENSOARG00000026637, ENSOARG00000026638* |
| 77 | 7 | 19020336 | 19092955 | 0.073 | 1 | *ARIH1* |
| 78 | 7 | 32867531 | 32906308 | 0.039 | 1 | *PAK6* |
| 79 | 7 | 32958587 | 32979020 | 0.020 | 1 | *CCDC9B* |
| 80 | 7 | 58048554 | 58156085 | 0.108 | 1 | *COPS2* |
| 81 | 7 | 57125244 | 57306802 | 0.182 | 2 | *ENSOARG00000026710, ATP8B4* |
| 82 | 8 | 51037707 | 51235881 | 0.198 | 2 | *SNX14, NT5E, U6* |
| 83 | 8 | 80330143 | 80433695 | 0.104 | 2 | *ARID1B, 5S_rRNA* |
| 84 | 8 | 63527401 | 63609947 | 0.083 | 3 | *ENSOARG00000002584, CCDC28A, ECT2L* |
| 85 | 8 | 27957061 | 28318981 | 0.362 | 8 | *ENSOARG00000010506, ENSOARG00000010556, ENSOARG00000010591, CEP57L1, SESN1, 5S_rRNA, U12, ARMC2* |
| 86 | 9 | 30898972 | 31000797 | 0.102 | 1 | *ENSOARG00000011053* |
| 87 | 9 | 68403247 | 68664408 | 0.261 | 1 | *EMC2* |
| 88 | 9 | 77232397 | 77238302 | 0.006 | 1 | *VPS13B* |
| 89 | 9 | 78203773 | 78336078 | 0.132 | 1 | *STK3* |
| 90 | 9 | 60361832 | 60425577 | 0.064 | 2 | *UTP23, EIF3H* |
| 91 | 9 | 77058773 | 77209510 | 0.151 | 2 | *VPS13B, SNORA70* |
| 92 | 9 | 25454929 | 25571827 | 0.117 | 4 | *ENSOARG00000024091, ENSOARG00000022417, ENSOARG00000008005, ENSOARG00000021647* |
| 93 | 9 | 28404754 | 28672894 | 0.268 | 5 | *MTSS1, NDUFB9, TATDN1, RNF139, TMEM65* |
| 94 | 9 | 77291492 | 77777273 | 0.486 | 5 | *VPS13B, ENSOARG00000023445, ENSOARG00000021235, ENSOARG00000014618, ENSOARG00000014632* |
| 95 | 10 | 28729635 | 28804261 | 0.075 | 1 | *N4BP2L2* |
| 96 | 10 | 75296806 | 75373517 | 0.077 | 1 | *DOCK9* |
| 97 | 10 | 7281005 | 7649664 | 0.369 | 2 | *ENSOARG00000006632, ENSOARG00000006641* |
| 98 | 10 | 70812659 | 70863686 | 0.051 | 2 | *ENSOARG00000001156, ENSOARG00000001163* |
| 99 | 10 | 19080326 | 19193184 | 0.113 | 4 | *FNDC3A, ENSOARG00000008427, ENSOARG00000021814, CDADC1* |
| 100 | 10 | 36524922 | 36651187 | 0.126 | 4 | *MPHOSPH8, PARP4, ENSOARG00000024226, U6* |
| 101 | 10 | 35839462 | 36385423 | 0.546 | 12 | *ENSOARG00000026300, LATS2, XPO4, EEF1AKMT1, ENSOARG00000017062, IFT88, CRYL1, GJB6, ENSOARG00000026301, GJB2, GJA3, ZMYM2* |
| 102 | 11 | 32887388 | 32909362 | 0.022 | 1 | *NCOR1* |
| 103 | 11 | 18307327 | 18476924 | 0.170 | 3 | *NF1, EVI2B, OMG* |
| 104 | 11 | 24590659 | 24774048 | 0.183 | 7 | *SPNS2, MYBBP1A, GGT6, TEKT1, SMTNL2, FBXO39, XAF1* |
| 105 | 12 | 42833548 | 43000355 | 0.167 | 1 | *ENSOARG00000009688* |
| 106 | 12 | 52701783 | 52701783 | 0.000 | 1 | *ENSOARG00000025458* |
| 107 | 12 | 45022062 | 45033986 | 0.012 | 2 | *ENSOARG00000011227, ENSOARG00000012422* |
| 108 | 12 | 52537159 | 52571434 | 0.034 | 2 | *ENSOARG00000011419, ENSOARG00000011458* |
| 109 | 12 | 52601005 | 52643767 | 0.043 | 2 | *ENSOARG00000011511, ENSOARG00000011567* |
| 110 | 13 | 49716270 | 49772494 | 0.056 | 1 | *ENSOARG00000004705* |
| 111 | 13 | 53213996 | 53219798 | 0.006 | 1 | *UCKL1* |
| 112 | 13 | 24611544 | 24711730 | 0.100 | 2 | *ARHGAP21, ENSOARG00000021630* |
| 113 | 13 | 38716844 | 38883136 | 0.166 | 2 | *CRNKL1, CFAP61* |
| 114 | 13 | 48812430 | 49113419 | 0.301 | 3 | *ENSOARG00000026237, ENSOARG00000026238, ENSOARG00000018756* |
| 115 | 13 | 53231481 | 53319729 | 0.088 | 3 | *DNAJC5, TPD52L2, ABHD16B* |
| 116 | 13 | 42439103 | 42769907 | 0.331 | 11 | *ENSOARG00000009642, ENSOARG00000009762, ENSOARG00000010028, ENSOARG00000010221, ENSOARG00000010355, ENSOARG00000010449, ENSOARG00000010488, ENSOARG00000010577, ENSOARG00000010661, ENSOARG00000010907, ENSOARG00000011087* |
| 117 | 13 | 53336545 | 53589429 | 0.253 | 16 | *ZBTB46, ENSOARG00000026240, ZGPAT, ARFRP1, ENSOARG00000010322, ENSOARG00000010368, STMN3, GMEB2, FNDC11, SRMS, PTK6, ENSOARG00000010995, EEF1A2, KCNQ2, CHRNA4, ENSOARG00000011265* |
| 118 | 14 | 37445689 | 37549975 | 0.104 | 1 | *ZFHX3* |
| 119 | 14 | 37987739 | 37987739 | 0.000 | 1 | *0* |
| 120 | 14 | 38603036 | 38641537 | 0.039 | 2 | *AP1G1, ENSOARG00000022908* |
| 121 | 14 | 38438487 | 38536041 | 0.098 | 4 | *PKD1L3, IST1, U6, ZNF821* |
| 122 | 14 | 38269991 | 38391153 | 0.121 | 5 | *ENSOARG00000003692, DHX38, TXNL4B, ENSOARG00000003744, DHODH* |
| 123 | 14 | 34418537 | 34595027 | 0.176 | 10 | *ATP6V0D1, AGRP, RIPOR1, CTCF, CARMIL2, ACD, PARD6A, ENKD1, C16orf86, GFOD2* |
| 124 | 15 | 42448209 | 42461989 | 0.014 | 1 | *SBF2* |
| 125 | 15 | 42234160 | 42393160 | 0.159 | 3 | *SBF2, ENSOARG00000017142, ENSOARG00000012115* |
| 126 | 16 | 47135448 | 47228081 | 0.093 | 1 | *ENSOARG00000026972* |
| 127 | 16 | 5353067 | 5454415 | 0.101 | 2 | *ENSOARG00000004192, ENSOARG00000005024* |
| 128 | 17 | 34524230 | 34545454 | 0.021 | 1 | *FGF2* |
| 129 | 17 | 62031439 | 62088590 | 0.057 | 1 | *BICDL1* |
| 130 | 17 | 52348125 | 52500613 | 0.152 | 3 | *HCAR1, ENSOARG00000004617, KNTC1* |
| 131 | 17 | 34270508 | 34501390 | 0.231 | 4 | *FGF2, ENSOARG00000023095, NUDT6, U4* |
| 132 | 18 | 22719475 | 22833399 | 0.114 | 1 | *BNC1* |
| 133 | 18 | 23475785 | 23580801 | 0.105 | 1 | *EFL1* |
| 134 | 18 | 66031948 | 66110749 | 0.079 | 1 | *RCOR1* |
| 135 | 18 | 32241562 | 32450099 | 0.209 | 10 | *PTPN9, SIN3A, MAN2C1, NEIL1, ENSOARG00000021972, COMMD4, ENSOARG00000002552, ENSOARG00000017499, ENSOARG00000002566, C15orf39* |
| 136 | 19 | 23155802 | 23322439 | 0.167 | 1 | *CNTN4* |
| 137 | 19 | 29147239 | 29294986 | 0.148 | 1 | *RYBP* |
| 138 | 19 | 34781609 | 34882846 | 0.101 | 1 | *ENSOARG00000024906* |
| 139 | 19 | 42432876 | 42536372 | 0.103 | 1 | *CFAP20DC* |
| 140 | 21 | 40208911 | 40291993 | 0.083 | 1 | *AHNAK, ROM1* |
| 141 | 21 | 36959560 | 37178672 | 0.219 | 5 | *ENSOARG00000001993, ENSOARG00000002825, ENSOARG00000003195, ENSOARG00000003295, ENSOARG00000003462* |
| 142 | 22 | 8910490 | 8973561 | 0.063 | 1 | *MINPP1* |
| 143 | 23 | 61043741 | 61194971 | 0.151 | 2 | *ENSOARG00000006125, ENSOARG00000006130* |
| 144 | 24 | 13087665 | 13188787 | 0.101 | 1 | *MRTFB* |
| 145 | 24 | 40930324 | 41006116 | 0.076 | 1 | *ENSOARG00000025913* |
| 146 | 25 | 4007616 | 4036762 | 0.029 | 1 | *C1orf131* |
| 147 | 25 | 18899644 | 18979364 | 0.080 | 1 | *ENSOARG00000026405* |
| 148 | 25 | 4069772 | 4138035 | 0.068 | 4 | *GNPAT, EXOC8, SPRTN, ENSOARG00000003271* |
| 149 | 26 | 1344691 | 1394564 | 0.050 | 1 | *ENSOARG00000026782* |
| 150 | 26 | 1506153 | 1925293 | 0.419 | 1 | *CSMD1* |

**Supplementary Table S5.** The candidate regions spanning genes within the SD_G2 sheep group identified *via* ROH islands.

| **Reg.** | **Chr.** | **Start** | **Stop** | **Size (Mb)** | **No. of**  **Genes** | **Genes** |
| --- | --- | --- | --- | --- | --- | --- |
| 1 | 1 | 50968109 | 51049783 | 0.082 | 1 | *SLC44A5* |
| 2 | 1 | 199686332 | 199724053 | 0.038 | 1 | *IGF2BP2* |
| 3 | 1 | 209329026 | 209352597 | 0.024 | 1 | *U6* |
| 4 | 1 | 215916258 | 216083412 | 0.167 | 1 | *ENSOARG00000014783* |
| 5 | 1 | 223176342 | 223213757 | 0.037 | 1 | *ENSOARG00000023528* |
| 6 | 1 | 223409393 | 223483815 | 0.074 | 1 | *ENSOARG00000025614* |
| 7 | 1 | 250519139 | 250845832 | 0.327 | 1 | *STAG1* |
| 8 | 1 | 105567842 | 105641892 | 0.074 | 2 | *ENSOARG00000022142, ETV3* |
| 9 | 1 | 154639663 | 154769287 | 0.130 | 2 | *ZNF654, C3orf38* |
| 10 | 1 | 102680214 | 102786943 | 0.107 | 3 | *NPR1, INTS3, SLC27A3* |
| 11 | 1 | 109751559 | 109957493 | 0.206 | 3 | *VANGL2, ENSOARG00000008861, CD84* |
| 12 | 1 | 128991659 | 129117452 | 0.126 | 3 | *MRPL39, ENSOARG00000022750, U6* |
| 13 | 1 | 255228404 | 255363529 | 0.135 | 3 | *DNAJC13, ENSOARG00000009070, ACP3* |
| 14 | 1 | 185150814 | 185292803 | 0.142 | 5 | *KPNA1, U6, PARP9, DTX3L, PARP14* |
| 15 | 2 | 14415007 | 14593219 | 0.178 | 1 | *ENSOARG00000007153* |
| 16 | 2 | 48753407 | 48767452 | 0.014 | 1 | *ANKS6* |
| 17 | 2 | 71242888 | 71276656 | 0.034 | 1 | *RFX3* |
| 18 | 2 | 177930202 | 178113912 | 0.184 | 1 | *ACTR3* |
| 19 | 2 | 232519985 | 232579393 | 0.059 | 1 | *COPS7B* |
| 20 | 2 | 232886414 | 233031304 | 0.145 | 1 | *DIS3L2* |
| 21 | 2 | 233305567 | 233448785 | 0.143 | 1 | *PHC2* |
| 22 | 2 | 51106073 | 51415207 | 0.309 | 2 | *ZCCHC7, PAX5* |
| 23 | 2 | 200735527 | 200875459 | 0.140 | 2 | *SATB2, U6* |
| 24 | 2 | 113009833 | 113108088 | 0.098 | 3 | *TUBGCP5, ENSOARG00000015810, IMP4* |
| 25 | 2 | 234293272 | 234310960 | 0.018 | 3 | *BSDC1, TSSK3, FAM229A* |
| 26 | 2 | 219201079 | 219324573 | 0.123 | 6 | *ENSOARG00000019492, ENSOARG00000019495, ENSOARG00000025091, AAMP, PNKD, TMBIM1* |
| 27 | 2 | 183938177 | 184484474 | 0.546 | 7 | *CFAP221, ENSOARG00000012769, PTPN4, EPB41L5, U4, TMEM185B, RALB* |
| 28 | 2 | 203429860 | 203630400 | 0.201 | 7 | *SUMO1, ENSOARG00000024976, NOP58, ENSOARG00000021853, ENSOARG00000022874, ENSOARG00000023154, ENSOARG00000023031* |
| 29 | 2 | 234403000 | 234463214 | 0.060 | 7 | *ENSOARG00000000244, ENSOARG00000000261, ENSOARG00000000281, DCDC2B, IQCC, TXLNA, KPNA6* |
| 30 | 3 | 11324243 | 11324243 | 0.000 | 1 | *ADGRD2* |
| 31 | 3 | 11763552 | 11825668 | 0.062 | 1 | *ENSOARG00000013754* |
| 32 | 3 | 46641216 | 46716559 | 0.075 | 1 | *USP34* |
| 33 | 3 | 63368153 | 63453835 | 0.086 | 1 | *BCL11A* |
| 34 | 3 | 154176421 | 154409242 | 0.233 | 1 | *MSRB3* |
| 35 | 3 | 172095135 | 172185595 | 0.090 | 1 | *ENSOARG00000015894* |
| 36 | 3 | 10512650 | 10583705 | 0.071 | 2 | *MAPKAP1, U5* |
| 37 | 3 | 11366422 | 11437859 | 0.071 | 2 | *PSMB7, NEK6* |
| 38 | 3 | 19720993 | 19816057 | 0.095 | 2 | *ATP6V1C2, PDIA6* |
| 39 | 3 | 28418373 | 28588659 | 0.170 | 2 | *LDAH, U6* |
| 40 | 3 | 46380758 | 46521876 | 0.141 | 2 | *ENSOARG00000025972, XPO1* |
| 41 | 3 | 46738461 | 46760579 | 0.022 | 2 | *USP34, ENSOARG00000020279* |
| 42 | 3 | 124326080 | 124428154 | 0.102 | 2 | *CEP290, TMTC3* |
| 43 | 3 | 129387135 | 129958737 | 0.572 | 5 | *ENSOARG00000015830, ENSOARG00000015838, MRPL42, SOCS2, CRADD* |
| 44 | 3 | 136002815 | 136089663 | 0.087 | 6 | *ENSOARG00000017904, COX14, ENSOARG00000016874, GPD1, SMARCD1, ASIC1* |
| 45 | 3 | 106033284 | 107108327 | 1.075 | 10 | *MERTK, TMEM87B, FBLN7, ZC3H8, ZC3H6, ENSOARG00000014519, ENSOARG00000016975, ENSOARG00000014533, ENSOARG00000024141, TSPAN8* |
| 46 | 3 | 10718038 | 11320116 | 0.602 | 16 | *HSPA5, RABEPK, PPP6C, SCAI, ENSOARG00000025028, GOLGA1, ARPC5L, ENSOARG00000013275, WDR38, U6, OLFML2A, ENSOARG00000023155, oar-mir-181a-2, NR6A1, NR5A1, ADGRD2* |
| 47 | 4 | 69907862 | 69988412 | 0.081 | 1 | *oar-mir-148a* |
| 48 | 4 | 69992611 | 70023831 | 0.031 | 1 | *ENSOARG00000025241* |
| 49 | 4 | 101878155 | 102073758 | 0.196 | 1 | *TRIM24* |
| 50 | 4 | 24475270 | 24581181 | 0.106 | 2 | *ENSOARG00000024373, ENSOARG00000024724* |
| 51 | 4 | 101473029 | 101587131 | 0.114 | 2 | *CREB3L2, AKR1D1* |
| 52 | 4 | 103833470 | 103915298 | 0.082 | 2 | *BRAF, 5S_rRNA* |
| 53 | 4 | 48631761 | 48727664 | 0.096 | 3 | *BCAP29, ENSOARG00000024106, SLC26A4* |
| 54 | 4 | 101347469 | 101387317 | 0.040 | 3 | *ENSOARG00000025256, CREB3L2, 7SK* |
| 55 | 4 | 94273495 | 94445213 | 0.172 | 9 | *ENSOARG00000022095, ENSOARG00000021327, ENSOARG00000022560, MEST, ENSOARG00000024045, COPG2, ENSOARG00000025252, ENSOARG00000025253, TSGA13* |
| 56 | 5 | 16684125 | 16761961 | 0.078 | 1 | *KDM4B* |
| 57 | 5 | 49868921 | 49929170 | 0.060 | 1 | *ENSOARG00000000218* |
| 58 | 5 | 57886689 | 57947932 | 0.061 | 1 | *SH3TC2* |
| 59 | 5 | 48741768 | 48852101 | 0.110 | 2 | *ENSOARG00000017448, PFDN1* |
| 60 | 5 | 107463423 | 107559150 | 0.096 | 2 | *CAMK4, ENSOARG00000025339* |
| 61 | 6 | 37438522 | 37615241 | 0.177 | 1 | *ENSOARG00000004333* |
| 62 | 6 | 69016328 | 69017663 | 0.001 | 1 | *LNX1* |
| 63 | 6 | 115404418 | 115416335 | 0.012 | 2 | *ENSOARG00000015067, FAM193A* |
| 64 | 6 | 116529885 | 116599409 | 0.070 | 2 | *ENSOARG00000016282, GAK* |
| 65 | 6 | 24689427 | 24942922 | 0.253 | 6 | *ENSOARG00000013905, ENSOARG00000023729, H2AZ1, DNAJB14, LAMTOR3, DAPP1* |
| 66 | 6 | 116791619 | 116982158 | 0.191 | 12 | *ENSOARG00000008606, MAEA, ENSOARG00000016735, ENSOARG00000016756, SLC49A3, PDE6B, PIGG, ENSOARG00000026634, ENSOARG00000026635, ENSOARG00000026636, ENSOARG00000026637, ENSOARG00000026638* |
| 67 | 7 | 55764564 | 55852119 | 0.088 | 1 | *DMXL2* |
| 68 | 7 | 55892093 | 55901243 | 0.009 | 1 | *DMXL2* |
| 69 | 7 | 55980542 | 56060222 | 0.080 | 1 | *GLDN* |
| 70 | 7 | 21019784 | 21095002 | 0.075 | 2 | *ZFHX2, NGDN* |
| 71 | 7 | 32220200 | 32267905 | 0.048 | 2 | *THBS1, FSIP1* |
| 72 | 7 | 34293259 | 34382138 | 0.089 | 2 | *SPTBN5, EHD4* |
| 73 | 7 | 57125244 | 57422986 | 0.298 | 2 | *ENSOARG00000026710, ATP8B4* |
| 74 | 8 | 51065356 | 51235881 | 0.171 | 3 | *SNX14, NT5E, U6* |
| 75 | 8 | 90416289 | 90503066 | 0.087 | 4 | *ENSOARG00000005108, ENSOARG00000027055, ENSOARG00000027056, ENSOARG00000027057* |
| 76 | 8 | 90545126 | 90680086 | 0.135 | 4 | *FAM120B, PSMB1, ENSOARG00000005142, PDCD2* |
| 77 | 9 | 30898972 | 31000797 | 0.102 | 1 | *ENSOARG00000011053* |
| 78 | 9 | 77215981 | 77216131 | 0.000 | 1 | *VPS13B* |
| 79 | 9 | 78191748 | 78207831 | 0.016 | 1 | *STK3* |
| 80 | 9 | 78234059 | 78323491 | 0.089 | 1 | *STK3* |
| 81 | 9 | 77058773 | 77185575 | 0.127 | 2 | *VPS13B, SNORA70* |
| 82 | 9 | 28378195 | 28622115 | 0.244 | 5 | *MTSS1, NDUFB9, TATDN1, RNF139, TMEM65* |
| 83 | 9 | 77291492 | 77667036 | 0.376 | 5 | *VPS13B, ENSOARG00000023445, ENSOARG00000021235, ENSOARG00000014618, ENSOARG00000014632* |
| 84 | 10 | 35950898 | 35950898 | 0.000 | 2 | *ENSOARG00000026300, XPO4* |
| 85 | 10 | 28729635 | 28905416 | 0.176 | 3 | *N4BP2L2, N4BP2L1, BRCA2* |
| 86 | 10 | 7281005 | 7690948 | 0.410 | 4 | *ENSOARG00000006632, ENSOARG00000006641, ENSOARG00000026268, ENSOARG00000006647* |
| 87 | 10 | 19193184 | 19306682 | 0.113 | 4 | *CDADC1, CAB39L, 5S_rRNA, 5S_rRNA* |
| 88 | 10 | 18972913 | 19183726 | 0.211 | 5 | *ENSOARG00000005934, FNDC3A, ENSOARG00000008427, ENSOARG00000021814, CDADC1* |
| 89 | 10 | 35990480 | 36163403 | 0.173 | 5 | *ENSOARG00000026300, EEF1AKMT1, ENSOARG00000017062, IFT88, CRYL1* |
| 90 | 10 | 36227134 | 36380305 | 0.153 | 5 | *GJB6, ENSOARG00000026301, GJB2, GJA3, ZMYM2* |
| 91 | 11 | 27029410 | 27042413 | 0.013 | 1 | *DNAH2* |
| 92 | 11 | 51283288 | 51361005 | 0.078 | 1 | *RNF213* |
| 93 | 11 | 55341260 | 55372253 | 0.031 | 3 | *SLC16A5, ENSOARG00000011379, ENSOARG00000022741* |
| 94 | 11 | 29018370 | 29105086 | 0.087 | 4 | *ENSOARG00000009609, ENSOARG00000011486, ENSOARG00000012656, ENSOARG00000026363* |
| 95 | 11 | 24548996 | 24774048 | 0.225 | 8 | *SPNS3, SPNS2, MYBBP1A, GGT6, TEKT1, SMTNL2, FBXO39, XAF1* |
| 96 | 11 | 27427935 | 27545955 | 0.118 | 8 | *CTC1, ENSOARG00000000318, ENSOARG00000000380, RANGRF, SLC25A35, ARHGEF15, ENSOARG00000001044, KRBA2, RPL26* |
| 97 | 11 | 26871682 | 27019326 | 0.148 | 10 | *CD68, MPDU1, SOX15, FXR2, SAT2, SHBG, ATP1B2, TP53, WRAP53, EFNB3* |
| 98 | 12 | 26761466 | 26818785 | 0.057 | 1 | *ENAH* |
| 99 | 12 | 42798981 | 42906614 | 0.108 | 1 | *ENSOARG00000009688* |
| 100 | 12 | 52800583 | 52808039 | 0.007 | 1 | *AADACL3* |
| 101 | 12 | 54009320 | 54199922 | 0.191 | 1 | *RABGAP1L* |
| 102 | 13 | 33415419 | 33514937 | 0.100 | 1 | *ZNF438* |
| 103 | 13 | 39638738 | 39642085 | 0.003 | 1 | *KIZ* |
| 104 | 13 | 49686957 | 49950396 | 0.263 | 1 | *ENSOARG00000004705* |
| 105 | 13 | 24627078 | 24705262 | 0.078 | 2 | *ARHGAP21, ENSOARG00000021630* |
| 106 | 13 | 48853122 | 49113419 | 0.260 | 2 | *ENSOARG00000026238, ENSOARG00000018756* |
| 107 | 13 | 56326781 | 56522417 | 0.196 | 2 | *ENSOARG00000015902, ZNF831* |
| 108 | 13 | 51425351 | 51488215 | 0.063 | 3 | *OXT, ENSOARG00000004656, PTPRA* |
| 109 | 13 | 69323416 | 69457126 | 0.134 | 5 | *ENSOARG00000001406, U6, PLCG1, ENSOARG00000021923, ENSOARG00000002038* |
| 110 | 13 | 42554038 | 42769907 | 0.216 | 6 | *ENSOARG00000010449, ENSOARG00000010488, ENSOARG00000010577, ENSOARG00000010661, ENSOARG00000010907, ENSOARG00000011087* |
| 111 | 13 | 53111186 | 53294265 | 0.183 | 10 | *ENSOARG00000008219, RGS19, TCEA2, PRPF6, U6, ENSOARG00000009022, ZNF512B, UCKL1, ENSOARG00000022250, DNAJC5* |
| 112 | 13 | 53336545 | 53589429 | 0.253 | 10 | *ZBTB46, ENSOARG00000026240, ZGPAT, ARFRP1, ENSOARG00000010322, ENSOARG00000010368, STMN3, GMEB2, FNDC11, SRMS* |
| 113 | 14 | 38438487 | 38557450 | 0.119 | 5 | *PKD1L3, IST1, U6, ZNF821, ATXN1L* |
| 114 | 14 | 38603036 | 38730984 | 0.128 | 5 | *AP1G1, ENSOARG00000022908, PHLPP2, U6, MARVELD3* |
| 115 | 14 | 34645791 | 34761020 | 0.115 | 9 | *RANBP10, TSNAXIP1, CENPT, ENSOARG00000003196, NUTF2, EDC4, PSKH1, PSMB10, LCAT* |
| 116 | 14 | 34418537 | 34614488 | 0.196 | 10 | *ATP6V0D1, AGRP, RIPOR1, CTCF, CARMIL2, ACD, PARD6A, ENKD1, C16orf86, GFOD2* |
| 117 | 15 | 52057707 | 52138998 | 0.081 | 1 | *ENSOARG00000010224* |
| 118 | 15 | 42296276 | 42393160 | 0.097 | 3 | *SBF2, ENSOARG00000017142, ENSOARG00000012115* |
| 119 | 16 | 70528271 | 70733910 | 0.206 | 8 | *ENSOARG00000026989, ENSOARG00000015678, ENSOARG00000015729, ENSOARG00000015756, 5S_rRNA, EXOC3, ENSOARG00000026990, SLC9A3* |
| 120 | 17 | 477601 | 824129 | 0.347 | 1 | *CPE* |
| 121 | 17 | 37521 | 245423 | 0.208 | 2 | *TMEM192, KLHL2* |
| 122 | 17 | 62025346 | 62166166 | 0.141 | 4 | *BICDL1, ENSOARG00000010963, RAB35, GCN1* |
| 123 | 17 | 52251562 | 52500613 | 0.249 | 8 | *VPS37B, HIP1R, CCDC62, DENR, SNORA70, HCAR1, ENSOARG00000004617, KNTC1* |
| 124 | 18 | 58634217 | 58681929 | 0.048 | 1 | *DICER1* |
| 125 | 18 | 32269663 | 32451742 | 0.182 | 10 | *PTPN9, SIN3A, MAN2C1, NEIL1, ENSOARG00000021972, COMMD4, ENSOARG00000002552, ENSOARG00000017499, ENSOARG00000002566, C15orf39* |
| 126 | 19 | 23182985 | 23322439 | 0.139 | 1 | *CNTN4* |
| 127 | 19 | 29157205 | 29293044 | 0.136 | 1 | *RYBP* |
| 128 | 19 | 56750636 | 56873922 | 0.123 | 1 | *SYN2* |
| 129 | 20 | 8446449 | 8475063 | 0.029 | 1 | *NUDT3* |
| 130 | 20 | 50142946 | 50206384 | 0.063 | 1 | *GMDS* |
| 131 | 20 | 17148477 | 17156484 | 0.008 | 2 | *POLR1C, XPO5* |
| 132 | 20 | 50958337 | 51083803 | 0.125 | 2 | *IRF4, DUSP22* |
| 133 | 20 | 17177812 | 17254423 | 0.077 | 6 | *XPO5, POLH, GTPBP2, MAD2L1BP, 5S_rRNA, RSPH9* |
| 134 | 20 | 16646531 | 16720282 | 0.074 | 7 | *PTCRA, CNPY3, GNMT, PEX6, PPP2R5D, ENSOARG00000004853, KLHDC3* |
| 135 | 21 | 35604235 | 35669032 | 0.065 | 1 | *ZBTB44* |
| 136 | 21 | 38518624 | 38519133 | 0.001 | 1 | *ENSOARG00000026107* |
| 137 | 21 | 46517968 | 46631317 | 0.113 | 1 | *ANO1* |
| 138 | 21 | 40203904 | 40277812 | 0.074 | 2 | *AHNAK, ROM1* |
| 139 | 22 | 8854834 | 9049564 | 0.195 | 2 | *ENSOARG00000024902, MINPP1* |
| 140 | 22 | 50704118 | 50820764 | 0.117 | 8 | *CYP2E1, ECHS1, ENSOARG00000000663, ENSOARG00000000727, CALY, ZNF511, TUBGCP2, ENSOARG00000014978* |
| 141 | 23 | 36013290 | 36125701 | 0.112 | 1 | *ENSOARG00000009539* |
| 142 | 23 | 47384833 | 47495946 | 0.111 | 1 | *ENSOARG00000026190* |
| 143 | 23 | 61043741 | 61148992 | 0.105 | 1 | *ENSOARG00000006125* |
| 144 | 25 | 39166409 | 39205820 | 0.039 | 1 | *ENSOARG00000000408* |

**Supplementary Table S6** The candidate regions spanning genes within the SD_G1 (SD_G1 vs SD_G2, CN_G1 and CN_G2 sheep group identified via XP-EHH.

| **Reg.** | **Chr.** | **Start** | **Stop** | **Size (Mb)** | **No of**  **Genes** | **Genes** |
| --- | --- | --- | --- | --- | --- | --- |
| 1 | 1 | 122800001 | 123100000 | 0.300 | 4 | *KRTAP11-1, KAP7, KRTAP8-1, ENSOARG00000014128* |
| 2 | 1 | 128900001 | 129200000 | 0.300 | 4 | *JAM2, MRPL39, ENSOARG00000022750, U6* |
| 3 | 1 | 160500001 | 160800000 | 0.300 | 6 | *ARL6, ENSOARG00000017511, ENSOARG00000017529, 5S_rRNA, RIOX2, GABRR3* |
| 4 | 1 | 222800001 | 223100000 | 0.300 | 1 | *5S_rRNA* |
| 5 | 1 | 254900001 | 255500000 | 0.600 | 3 | *RNF11, TTC39A, EPS15* |
| 6 | 1 | 80900001 | 81200000 | 0.300 | 3 | *ENSOARG00000025540, U6, ENSOARG00000011145* |
| 7 | 1 | 101100001 | 101400000 | 0.300 | 4 | *ENSOARG00000021087, ENSOARG00000021093, CRNN, ENSOARG00000025550* |
| 8 | 1 | 185100001 | 185400000 | 0.300 | 6 | *FAM162A, WDR5B, KPNA1, U6, PARP9, DTX3L* |
| 9 | 1 | 234600001 | 234900000 | 0.300 | 5 | *IGSF10, MED12L, P2RY12, P2RY13, GPR87* |
| 10 | 2 | 9000001 | 9300000 | 0.300 | 3 | *TMEM268, ATP6V1G1, WHRN* |
| 11 | 2 | 111500001 | 111800000 | 0.300 | 6 | *MFSD14B, ENSOARG00000015612, ENSOARG00000009336, ENSOARG00000009365, 5S_rRNA, ENSOARG00000015627* |
| 12 | 2 | 14200001 | 14700000 | 0.500 | 8 | *TMEM245, ENSOARG00000021826, CTNNAL1, ABITRAM, ELP1, ENSOARG00000007143, ACTL7B, ENSOARG00000007153* |
| 13 | 2 | 52300001 | 52600000 | 0.300 | 19 | *TMEM8B, FAM221B, HINT2, SPAG8, NPR2, MSMP, RGP1, GBA2, CREB3, TLN1, TPM2, CA9, ARHGEF39, CCDC107, RNase_MRP, ENSOARG00000008318, SIT1,*  *CD72* |
| 14 | 2 | 52800001 | 53100000 | 0.300 | 7 | *ENSOARG00000012072, ENSOARG00000012105, FAM214B, STOML2,*  *PIGO, FANCG, VCP* |
| 15 | 2 | 113000001 | 113700000 | 0.700 | 8 | *TUBGCP5, ENSOARG00000015810, IMP4, PTPN18, ENSOARG00000015841, ENSOARG00000015852, AMER3, ENSOARG00000015869* |
| 16 | 2 | 122400001 | 122700000 | 0.300 | 2 | *FSIP2, ENSOARG00000025803* |
| 17 | 2 | 141000001 | 141300000 | 0.300 | 2 | *XIRP2, ENSOARG00000022371* |
| 18 | 3 | 10700001 | 11800000 | 1.100 | 19 | *HSPA5, RABEPK, PPP6C, SCAI, ENSOARG00000025028, GOLGA1, ARPC5L, ENSOARG00000013275, WDR38, U6, OLFML2A, ENSOARG00000023155, oar-mir-181a-2, NR6A1, NR5A1, ADGRD2, PSMB7, NEK6, LHX2* |
| 19 | 3 | 25400001 | 25700000 | 0.300 | 3 | *RAD51AP2, VSNL1, SMC6* |
| 20 | 3 | 124100001 | 124400000 | 0.300 | 5 | *KRTAP11-1, KAP7, KRTAP8-1, ENSOARG00000014128* |
| 21 | 3 | 206800001 | 207100000 | 0.300 | 7 | *ENSOARG00000023957, C12orf50, C3H12orf29, CEP290, TMTC3* |
| 22 | 3 | 131800001 | 132600000 | 0.800 | 19 | *ENSOARG00000003403, ENSOARG00000003494, ENSOARG00000003615, ENSOARG00000003690, ENSOARG00000003829, ENSOARG00000003988, ENSOARG00000004066* |
| 23 | 3 | 172200001 | 172500000 | 0.300 | 2 | *PPP1R1A, PDE1B, NCKAP1L, GTSF1, ITGA5, ZNF385A, COPZ1, NFE2, CBX5, HOXC4, HOXC5, HOXC6, HOXC8, HOXC9, HOXC10, HOXC11, HOXC12, HOXC13* |
| 24 | 3 | 10400001 | 11800000 | 1.400 | 22 | *STAB2, ENSOARG00000016149* |
| 25 | 3 | 12200001 | 12700000 | 0.500 | 6 | *MAPKAP1, U5, GAPVD1, HSPA5, RABEPK, PPP6C, SCAI, ENSOARG00000025028, GOLGA1, ARPC5L, ENSOARG00000013275, WDR38, U6, OLFML2A, ENSOARG00000023155, oar-mir-181a-2, NR6A1, NR5A1, ADGRD2,*  *PSMB7, NEK6, LHX2* |
| 26 | 3 | 135700001 | 136000000 | 0.300 | 5 | *ENSOARG00000013769, ENSOARG00000021273, CRB2, U6, ENSOARG00000013864, RABGAP1* |
| 27 | 4 | 48600001 | 48900000 | 0.300 | 6 | *ENSOARG00000017702, FAM186A, LIMA1, ENSOARG00000017895, ENSOARG00000017904* |
| 28 | 4 | 50600001 | 50900000 | 0.300 | 2 | *DUS4L, BCAP29, ENSOARG00000024106, SLC26A4, CBLL1, SLC26A3* |
| 29 | 4 | 55200001 | 55500000 | 0.300 | 2 | *CTTNBP2, CFTR* |
| 30 | 4 | 91700001 | 92000000 | 0.300 | 8 | *ZNF800, ENSOARG00000025250, GCC1, ARF5, ENSOARG00000001904, PAX4, SND1, ENSOARG00000022149* |
| 31 | 5 | 5400001 | 5700000 | 0.300 | 18 | *ENSOARG00000016641, MVB12A, BST-2B, BST-2A, PLVAP, U6, GTPBP3, ANO8, DDA1, ENSOARG00000017239, ABHD8, ANKLE1, BABAM1, USHBP1, NR2F6,*  *OCEL1, USE1, MYO9B* |
| 32 | 5 | 11000001 | 11300000 | 0.300 | 5 | *ENSOARG00000012729, ENSOARG00000012765, ENSOARG00000012799, ENSOARG00000013067, ENSOARG00000025290* |
| 33 | 5 | 41700001 | 42000000 | 0.300 | 11 | *ENSOARG00000013233, ENSOARG00000013103, SOWAHA, SHROOM1, GDF9, UQCRQ, LEAP2, AFF4, U6, ZCCHC10, ENSOARG00000013524* |
| 34 | 5 | 48700001 | 49000000 | 0.300 | 6 | *IGIP, ENSOARG00000017448, PFDN1, HBEGF, SLC4A9, ENSOARG00000017846* |
| 35 | 5 | 49000001 | 49400000 | 0.400 | 21 | *ENSOARG00000017846, 5S_rRNA, U6, SRA1, APBB3, ENSOARG00000018183, SLC35A4, ENSOARG00000018230, CD14, TMCO6, NDUFA2, IK, DND1, HARS1, HARS2, ZMAT2, Vault, PCDHA2, PCDHA5, ENSOARG00000018742, PCDHA11* |
| 36 | 5 | 49700001 | 50000000 | 0.300 | 10 | *PCDHB14, PCDHB15, ENSOARG00000014442, TAF7, PCDHGA1, PCDHGA2, ENSOARG00000000218, PCDHGC3, ENSOARG00000023940, DIAPH1* |
| 37 | 6 | 29900001 | 30200000 | 0.300 | 1 | *PDLIM5* |
| 38 | 6 | 85300001 | 85600000 | 0.300 | 6 | *CSN3, ENSOARG00000011161, CABS1, ENSOARG00000011228, ENSOARG00000008596, AMTN* |
| 39 | 6 | 69700001 | 70000000 | 0.300 | 2 | *PDGFRA, ENSOARG00000021645* |
| 40 | 6 | 71000001 | 71300000 | 0.300 | 6 | *PDCL2, ENSOARG00000022516, ENSOARG00000001999, U6, EXOC1L, EXOC1* |
| 41 | 6 | 83200001 | 83600000 | 0.400 | 7 | *CENPC, STAP1, UBA6, GNRHR, ENSOARG00000007652, TMPRSS11D, TMPRSS11A* |
| 42 | 6 | 85300001 | 85900000 | 0.600 | 10 | *CSN3, ENSOARG00000011161, CABS1, ENSOARG00000011228, ENSOARG00000008596, AMTN, AMBN, ENAM, JCHAIN, UTP3* |
| 43 | 6 | 37400001 | 37700000 | 0.300 | 1 | *ENSOARG00000004333* |
| 44 | 7 | 72300001 | 72600000 | 0.300 | 3 | *CMYA5, ENSOARG00000026690, SNORA72* |
| 45 | 7 | 10300001 | 10600000 | 0.300 | 6 | *SNAP23, ENSOARG00000020538, HAUS2, ENSOARG00000020558, CDAN1, TTBK2* |
| 46 | 7 | 34900001 | 35200000 | 0.300 | 5 | *RHOJ, ENSOARG00000021260, ENSOARG00000021143, PPP2R5E, ENSOARG00000000975* |
| 47 | 8 | 27800001 | 28200000 | 0.400 | 11 | *ZBTB24, MICAL1, SMPD2, PPIL6, U6, CD164, ENSOARG00000010506, ENSOARG00000010556, ENSOARG00000010591, CEP57L1, SESN1* |
| 48 | 8 | 59800001 | 60100000 | 0.300 | 5 | *5S_rRNA, ENSOARG00000003580, ALDH8A1, ENSOARG00000014798, ENSOARG00000025142* |
| 49 | 9 | 9800001 | 10100000 | 0.300 | 1 | *ENSOARG00000026490* |
| 50 | 9 | 28200001 | 28500000 | 0.300 | 2 | *PCDHB14, PCDHB15, ENSOARG00000014442, TAF7, PCDHGA1, PCDHGA2, ENSOARG00000000218, PCDHGC3, ENSOARG00000023940, DIAPH1* |
| 51 | 9 | 53600001 | 53900000 | 0.300 | 4 | *MTSS1, NDUFB9, TATDN1, RNF139* |
| 52 | 9 | 57000001 | 57600000 | 0.600 | 8 | *PAG1, ENSOARG00000026536, ENSOARG00000026537, FABP5, PMP2, FABP9, FABP4, FABP12* |
| 53 | 10 | 71700001 | 72100000 | 0.400 | 5 | *ENSOARG00000001553, ENSOARG00000001594, ENSOARG00000001672, ENSOARG00000001678,*  *ENSOARG00000001808* |
| 54 | 10 | 7100001 | 7600000 | 0.500 | 2 | *ENSOARG00000006632, ENSOARG00000006641* |
| 55 | 10 | 35800001 | 36800000 | 1.000 | 23 | *SAP18, ENSOARG00000026299, ENSOARG00000026300, LATS2, XPO4, EEF1AKMT1, ENSOARG00000017062, IFT88, CRYL1, GJB6, ENSOARG00000026301, GJB2, GJA3, ZMYM2, ENSOARG00000014110, ENSOARG00000014118, PSPC1, MPHOSPH8, PARP4, ENSOARG00000024226, U6, CENPJ, RNF17* |
| 56 | 10 | 42200001 | 42500000 | 0.300 | 3 | *ENSOARG00000015005, ENSOARG00000026305, ENSOARG00000026306* |
| 57 | 11 | 24400001 | 24900000 | 0.500 | 15 | *UBE2G1, SNORA62, SPNS3, SPNS2, MYBBP1A, GGT6, TEKT1, SMTNL2, FBXO39, XAF1, SLC13A5, ENSOARG00000001178, MED31, TXNDC17, KIAA0753* |
| 58 | 12 | 61300001 | 61600000 | 0.300 | 2 | *ENSOARG00000022514, ENSOARG00000025424* |
| 59 | 12 | 7500001 | 7800000 | 0.300 | 2 | *GLUL, TEDDM1* |
| 60 | 12 | 31500001 | 31800000 | 0.300 | 2 | *AKT3, SDCCAG8* |
| 61 | 12 | 66900001 | 67200000 | 0.300 | 2 | *CENPF, PTPN14* |
| 62 | 12 | 74000001 | 74300000 | 0.300 | 6 | *ENSOARG00000014055, ENSOARG00000014196, F13B, ENSOARG00000014405, ASPM, ZBTB41* |
| 63 | 13 | 9800001 | 10200000 | 0.400 | 7 | *ENSOARG00000011950, ENSOARG00000024050, U6, SNRPB2, OTOR, 5S_rRNA, NANP* |
| 64 | 13 | 42900001 | 43200000 | 0.300 | 3 | *ZNF438, ENSOARG00000026216, SVIL* |
| 65 | 13 | 33400001 | 33700000 | 0.300 | 6 | *ENSOARG00000011857, ENSOARG00000012196, ENSOARG00000012476, ENSOARG00000012680, AKR1E2, ENSOARG00000004635* |
| 66 | 13 | 42500001 | 42800000 | 0.300 | 9 | *ENSOARG00000010221, ENSOARG00000010355, ENSOARG00000010449, ENSOARG00000010488, ENSOARG00000010577, ENSOARG00000010661, ENSOARG00000010907, ENSOARG00000011087, ENSOARG00000011490* |
| 67 | 13 | 52900001 | 53200000 | 0.300 | 11 | *GINS1, ENSOARG00000007835, MYT1, NPBWR2, OPRL1, ENSOARG00000008219, RGS19, TCEA2, PRPF6, U6, ENSOARG00000009022* |
| 68 | 13 | 53200001 | 53500000 | 0.300 | 20 | *ENSOARG00000009022, ZNF512B, UCKL1, ENSOARG00000022250, DNAJC5, TPD52L2, ABHD16B, ZBTB46, ENSOARG00000026240, ZGPAT, ARFRP1, ENSOARG00000010322, ENSOARG00000010368, STMN3, GMEB2, FNDC11, SRMS, PTK6, ENSOARG00000010995, EEF1A2* |
| 69 | 14 | 38400001 | 38800000 | 0.400 | 14 | *LRRC36, TPPP3, ZDHHC1, HSD11B2, ATP6V0D1, AGRP, RIPOR1, CTCF, CARMIL2, ACD, PARD6A, ENKD1, C16orf86, GFOD2* |
| 70 | 14 | 34300001 | 34600000 | 0.300 | 9 | *PKD1L3, IST1, ZNF821, ATXN1L, AP1G1, PHLPP2, MARVELD3, U6, TAT* |
| 71 | 15 | 11200001 | 11500000 | 0.300 | 1 | *ENSOARG00000024785* |
| 72 | 15 | 42000001 | 42300000 | 0.300 | 1 | *SBF2* |
| 73 | 15 | 48800001 | 49300000 | 0.500 | 17 | *OR51D1, TRIM68, ENSOARG00000000867, ENSOARG00000000931, ENSOARG00000006564, ENSOARG00000006579, ENSOARG00000006597, ENSOARG00000006612, ENSOARG00000006628, TRIM21, ENSOARG00000006646, ENSOARG00000006656, ENSOARG00000006671, ENSOARG00000006682, ENSOARG00000006696, ENSOARG00000001126, RRM1* |
| 74 | 15 | 62900001 | 63200000 | 0.300 | 2 | *KIAA1549L, ENSOARG00000006878* |
| 75 | 15 | 62200001 | 62500000 | 0.300 | 4 | *NAT10, ABTB2, ENSOARG00000024404, CAT* |
| 76 | 17 | 66800001 | 67200000 | 0.400 | 7 | *ENSOARG00000001775, ENSOARG00000025691, PITPNB, ENSOARG00000024980, ENSOARG00000025692, TTC28, ENSOARG00000023311* |
| 77 | 17 | 34400001 | 34800000 | 0.400 | 5 | *FGF2, ENSOARG00000023095, NUDT6, U4, ENSOARG00000017596* |
| 78 | 17 | 62000001 | 62300000 | 0.300 | 9 | *ENSOARG00000025686, BICDL1, ENSOARG00000010963, RAB35, GCN1, ENSOARG00000011805, ENSOARG00000025687, PXN, ENSOARG00000025688* |
| 79 | 18 | 33700001 | 34100000 | 0.400 | 2 | *ENSOARG00000004815, STXBP6* |
| 80 | 18 | 35700001 | 36000000 | 0.300 | 1 | *NOVA1* |
| 81 | 19 | 29200001 | 29500000 | 0.300 | 1 | *RYBP* |
| 82 | 19 | 44800001 | 45100000 | 0.300 | 6 | *ENSOARG00000014502, ENSOARG00000014527, CMC1, AZI2, ENSOARG00000014615, ENSOARG00000024214* |
| 83 | 19 | 2500001 | 2800000 | 0.300 | 1 | *ERC2* |
| 84 | 20 | 44900001 | 45200000 | 0.300 | 1 | *ENSOARG00000016571* |
| 85 | 20 | 3500001 | 3900000 | 0.400 | 1 | *ENSOARG00000005969* |
| 86 | 25 | 42500001 | 42800000 | 0.300 | 2 | *ARHGAP22, WDFY4* |
| 87 | 26 | 1800001 | 2100000 | 0.300 | 1 | *MYOM2* |
| 88 | 26 | 1200001 | 1500000 | 0.300 | 1 | *ENSOARG00000026782* |
| 89 | 26 | 1500001 | 2000000 | 0.500 | 1 | *CSMD1* |

**Supplementary Table S7**. The candidate regions spanning genes within the SD_G2 (SD_G2 vs SD_G1, CN_G1 and CN_G2 sheep group identified via XP-EHH.

| **Reg.** | **Chr.** | **Start** | **Stop** | **Size (Mb)** | **No of**  **Genes** | **Genes** |
| --- | --- | --- | --- | --- | --- | --- |
| 1 | 1 | 102600001 | 102900000 | 0.300 | 12 | *ENSOARG00000025556, S100A16, S100A1, CHTOP, SNAPIN, ILF2, NPR1, INTS3, SLC27A3, ENSOARG00000001449, GATAD2B, DENND4B* |
| 2 | 1 | 198700001 | 199000000 | 0.300 | 7 | *KNG1, HRG, FETUB, AHSG, ENSOARG00000020530, TBCCD1, CRYGS* |
| 3 | 1 | 128900001 | 129200000 | 0.300 | 4 | *JAM2, MRPL39, ENSOARG00000022750, U6* |
| 4 | 1 | 209800001 | 210200000 | 0.400 | 1 | *ENSOARG00000020722* |
| 5 | 1 | 255200001 | 255500000 | 0.300 | 3 | *DNAJC13, ENSOARG00000009070, ACP3* |
| 6 | 1 | 101100001 | 101400000 | 0.300 | 4 | *ENSOARG00000021087, ENSOARG00000021093, CRNN, ENSOARG00000025550* |
| 7 | 1 | 154900001 | 155200000 | 0.300 | 1 | *ENSOARG00000001385* |
| 8 | 1 | 160500001 | 160800000 | 0.300 | 6 | *ARL6, ENSOARG00000017511, ENSOARG00000017529, 5S_rRNA, RIOX2, GABRR3* |
| 9 | 1 | 184300001 | 184600000 | 0.300 | 8 | *POLQ, ENSOARG00000020026, FBXO40, HCLS1, GOLGB1, IQCB1, ENSOARG00000020073, EAF2* |
| 10 | 1 | 185100001 | 185400000 | 0.300 | 9 | *FAM162A, WDR5B, KPNA1, U6, PARP9, DTX3L, PARP14, HSPBAP1, SLC49A4* |
| 11 | 2 | 149300001 | 149600000 | 0.300 | 3 | *WDSUB1, TANC1, ENSOARG00000025811* |
| 12 | 2 | 9000001 | 9300000 | 0.300 | 3 | *TMEM268, ATP6V1G1, WHRN* |
| 13 | 2 | 81800001 | 82100000 | 0.300 | 1 | *ENSOARG00000022981* |
| 14 | 2 | 87900001 | 88300000 | 0.400 | 2 | *5S_rRNA, MLLT3* |
| 15 | 2 | 183700001 | 184600000 | 0.900 | 9 | *TMEM37, SCTR, CFAP221, ENSOARG00000012769, PTPN4, EPB41L5, U4, TMEM185B, RALB* |
| 16 | 2 | 2800001 | 3100000 | 0.300 | 2 | *MEGF9, CDK5RAP2* |
| 17 | 2 | 14200001 | 14700000 | 0.500 | 8 | *TMEM245, ENSOARG00000021826, CTNNAL1, ABITRAM, ELP1, ENSOARG00000007143, ACTL7B, ENSOARG00000007153* |
| 18 | 2 | 114400001 | 114700000 | 0.300 | 1 | *ENSOARG00000025799* |
| 19 | 2 | 122400001 | 122700000 | 0.300 | 2 | *FSIP2, ENSOARG00000025803* |
| 20 | 2 | 141000001 | 141300000 | 0.300 | 2 | *XIRP2, ENSOARG00000022371* |
| 21 | 2 | 184300001 | 184600000 | 0.300 | 4 | *EPB41L5, U4, TMEM185B, RALB* |
| 22 | 2 | 229000001 | 229300000 | 0.300 | 4 | *CCL20, DAW1, U6, SPHKAP* |
| 23 | 3 | 51500001 | 51800000 | 0.300 | 1 | *ENSOARG00000020384* |
| 24 | 3 | 88800001 | 89100000 | 0.300 | 2 | *ENSOARG00000025998, U7* |
| 25 | 3 | 187700001 | 188000000 | 0.300 | 4 | *ENSOARG00000019857, ENSOARG00000019877, ITPR2, 5S_rRNA* |
| 26 | 3 | 192700001 | 193100000 | 0.400 | 5 | *ST8SIA1, ENSOARG00000023574, ENSOARG00000020181, CMAS, ABCC9* |
| 27 | 3 | 213200001 | 213500000 | 0.300 | 10 | *MICAL3, PEX26, TUBA8, CDC42EP1, LGALS2, GGA1, ENSOARG00000013931, PDXP, ENSOARG00000023734, U6* |
| 28 | 3 | 10700001 | 12500000 | 1.800 | 26 | *HSPA5, RABEPK, PPP6C, SCAI, ENSOARG00000025028, GOLGA1, ARPC5L, ENSOARG00000013275, WDR38, U6, OLFML2A, ENSOARG00000023155, oar-mir-181a-2, NR6A1, NR5A1, ADGRD2, PSMB7, NEK6, LHX2, ENSOARG00000013754, ENSOARG00000019124, ENSOARG00000013769, ENSOARG00000021273, CRB2, U6, ENSOARG00000013864* |
| 29 | 3 | 25400001 | 25700000 | 0.300 | 3 | *RAD51AP2, VSNL1, SMC6* |
| 30 | 3 | 107100001 | 107500000 | 0.400 | 4 | *TSPAN8, ENSOARG00000026011, LGR5, U6* |
| 31 | 3 | 124100001 | 124400000 | 0.300 | 5 | *ENSOARG00000023957, C12orf50, C3H12orf29, CEP290, TMTC3* |
| 32 | 3 | 129200001 | 129800000 | 0.600 | 4 | *ENSOARG00000015830, ENSOARG00000015838, MRPL42, SOCS2* |
| 33 | 3 | 143000001 | 143400000 | 0.400 | 2 | *ADAMTS20, U6* |
| 34 | 3 | 192700001 | 193000000 | 0.300 | 4 | *ST8SIA1, ENSOARG00000023574, ENSOARG00000020181, CMAS* |
| 35 | 3 | 206700001 | 207100000 | 0.400 | 8 | *ENSOARG00000003251, ENSOARG00000003403, ENSOARG00000003494, ENSOARG00000003615, ENSOARG00000003690, ENSOARG00000003829, ENSOARG00000003988, ENSOARG00000004066* |
| 36 | 3 | 10200001 | 11800000 | 1.600 | 18 | *ENSOARG00000012658, MAPKAP1, GAPVD1, HSPA5, RABEPK, PPP6C, SCAI, GOLGA1, ARPC5L, ENSOARG00000013275, WDR38, OLFML2A, NR6A1, NR5A1, ADGRD2, PSMB7, NEK6, LHX2* |
| 37 | 3 | 12200001 | 12800000 | 0.600 | 13 | *ENSOARG00000013769, CRB2, ENSOARG00000013864, RABGAP1, RC3H2, ZBTB6, ENSOARG00000021273, ENSOARG00000022036, U6, ENSOARG00000023155, U5, oar-mir-181a-2, ENSOARG00000025028* |
| 38 | 3 | 106100001 | 106500000 | 0.400 | 5 | *TMEM87B, FBLN7, ZC3H8, ZC3H6, ENSOARG00000014519* |
| 39 | 3 | 107500001 | 107800000 | 0.300 | 6 | *LGR5, ZFC3H1, THAP2, TMEM19, ENSOARG00000014640, TBC1D15* |
| 40 | 3 | 129300001 | 129900000 | 0.600 | 5 | *ENSOARG00000015830, ENSOARG00000015838, MRPL42, SOCS2, CRADD* |
| 41 | 3 | 135700001 | 136000000 | 0.300 | 5 | *ENSOARG00000017702, FAM186A, LIMA1, ENSOARG00000017895, ENSOARG00000017904* |
| 42 | 3 | 68400001 | 68900000 | 0.500 | 9 | *PPP4R3B, ENSOARG00000002309, CCDC88A, ENSOARG00000002488, MTIF2, ENSOARG00000016082, RPS27A, CLHC1, RTN4* |
| 43 | 4 | 94100001 | 94400000 | 0.300 | 11 | *CPA5, CPA1, CEP41, ENSOARG00000022095, ENSOARG00000021327, ENSOARG00000022560, MEST, ENSOARG00000024045, COPG2, ENSOARG00000025252, ENSOARG00000025253* |
| 44 | 4 | 98500001 | 98800000 | 0.300 | 5 | *CALD1, AGBL3, U6, TMEM140, CYREN* |
| 45 | 4 | 42400001 | 42800000 | 0.400 | 1 | *MAGI2* |
| 46 | 4 | 48600001 | 48900000 | 0.300 | 6 | *DUS4L, BCAP29, ENSOARG00000024106, SLC26A4, CBLL1, SLC26A3* |
| 47 | 4 | 68500001 | 68900000 | 0.400 | 20 | *ENSOARG00000009300, ENSOARG00000025240, EVX1, ENSOARG00000021905, ENSOARG00000009477, ENSOARG00000021798, ENSOARG00000022652, ENSOARG00000021661, ENSOARG00000009550, ENSOARG00000022033, ENSOARG00000023523, ENSOARG00000021279, ENSOARG00000022189, ENSOARG00000023214, ENSOARG00000021298, HOXA10, ENSOARG00000009680, ENSOARG00000009717, HOXA6, ENSOARG00000008482* |
| 48 | 4 | 1000001 | 1300000 | 0.300 | 2 | *ENSOARG00000013313, ENSOARG00000013322* |
| 49 | 4 | 1300001 | 1600000 | 0.300 | 3 | *U6, ENSOARG00000025201, ENSOARG00000025202* |
| 50 | 4 | 10300001 | 10600000 | 0.300 | 5 | *U6, SAMD9, HEPACAM2, VPS50, ENSOARG00000024682* |
| 51 | 5 | 10300001 | 10600000 | 0.400 | 1 | *SNCAIP* |
| 52 | 5 | 28500001 | 28900000 | 0.300 | 1 | *SNCAIP* |
| 53 | 5 | 75200001 | 75500000 | 0.300 | 3 | *U6, ENSOARG00000022252, ENSOARG00000025319* |
| 54 | 5 | 75000001 | 75300000 | 0.300 | 1 | *ENSOARG00000025319* |
| 55 | 5 | 17000001 | 17300000 | 0.300 | 10 | *DPP9, MYDGF, TNFAIP8L1, SEMA6B, LRG1, PLIN5, ENSOARG00000009400, HDGFL2, UBXN6, CHAF1A* |
| 56 | 5 | 41700001 | 42000000 | 0.300 | 11 | *ENSOARG00000013233, ENSOARG00000013103, SOWAHA, SHROOM1, GDF9, UQCRQ, LEAP2, AFF4, U6, ZCCHC10, ENSOARG00000013524* |
| 57 | 5 | 49700001 | 50000000 | 0.300 | 10 | *PCDHB14, PCDHB15, ENSOARG00000014442, TAF7, PCDHGA1, PCDHGA2, ENSOARG00000000218, PCDHGC3, ENSOARG00000023940, DIAPH1* |
| 58 | 6 | 42000001 | 42300000 | 0.300 | 2 | *ENSOARG00000013524, FSTL4* |
| 59 | 6 | 85300001 | 85600000 | 0.300 | 6 | *CSN3, ENSOARG00000011161, CABS1, ENSOARG00000011228, ENSOARG00000008596, AMTN* |
| 60 | 6 | 24800001 | 25100000 | 0.300 | 7 | *ENSOARG00000023729, H2AZ1, DNAJB14, LAMTOR3, DAPP1, C4orf54, MTTP* |
| 61 | 6 | 55600001 | 55900000 | 0.300 | 2 | *ARAP2, DTHD1* |
| 62 | 7 | 55600001 | 55900000 | 0.300 | 3 | *CMYA5, ENSOARG00000026690, SNORA72* |
| 63 | 7 | 10300001 | 10600000 | 0.300 | 6 | *MGA, MAPKBP1, JMJD7, ENSOARG00000020432, SPTBN5, EHD4* |
| 64 | 7 | 34100001 | 34400000 | 0.300 | 3 | *LYSMD2, SCG3, DMXL2* |
| 65 | 7 | 34900001 | 35200000 | 0.300 | 6 | *SNAP23, ENSOARG00000020538, HAUS2, ENSOARG00000020558, CDAN1, TTBK2* |
| 66 | 8 | 31500001 | 31800000 | 0.300 | 1 | *PREP* |
| 67 | 8 | 32600001 | 32900000 | 0.500 | _ |  |
| 68 | 8 | 37000001 | 37500000 | 0.300 | 8 | *ENSOARG00000011749, PRDM13, U6, CCNC, ENSOARG00000011806, USP45, PNISR, COQ3* |
| 69 | 8 | 70700001 | 71000000 | 0.300 | 2 | *RAB32, ADGB* |
| 70 | 8 | 32900001 | 33200000 | 0.300 | _ |  |
| 71 | 8 | 50900001 | 51200000 | 0.300 | 5 | *ENSOARG00000022331, ENSOARG00000022788, SYNCRIP, SNX14, NT5E* |
| 72 | 9 | 73800001 | 74100000 | 0.300 | 3 | *ENSOARG00000026490, ENSOARG00000026491, ENSOARG00000026492,* |
| 73 | 9 | 9800001 | 10100000 | 0.300 | 4 | *FZD6, ENSOARG00000016701, SNORD22, ATP6V1C1* |
| 74 | 9 | 28200001 | 28500000 | 0.300 | 4 | *MTSS1, NDUFB9, TATDN1, RNF139* |
| 75 | 9 | 74000001 | 74300000 | 0.400 | 3 | *SNORD22, ATP6V1C1, AZIN1* |
| 76 | 10 | 50000001 | 50400000 | 0.300 | _ |  |
| 77 | 10 | 78200001 | 78500000 | 0.500 | 7 | *TPP2, METTL21C, ENSOARG00000005160, TEX30, POGLUT2, ENSOARG00000005295, ENSOARG00000005325* |
| 78 | 10 | 7100001 | 7600000 | 0.300 | 2 | *ENSOARG00000006632, ENSOARG00000006641* |
| 79 | 10 | 42200001 | 42500000 | 0.500 | 3 | *ENSOARG00000015005, ENSOARG00000026305, ENSOARG00000026306* |
| 80 | 11 | 19000001 | 19500000 | 0.500 | 14 | *NOS2, ENSOARG00000017146, ENSOARG00000022598, U6, ENSOARG00000026356, NLK, TMEM97, IFT20, TNFAIP1, POLDIP2, TMEM199, SEBOX, ENSOARG00000018004, SARM1* |
| 81 | 11 | 25500001 | 26000000 | 0.300 | 12 | *NLRP1, ENSOARG00000004247, ENSOARG00000004258, DERL2, DHX33, C1QBP, RPAIN, NUP88, RABEP1, SCIMP, ENSOARG00000004260, ZFP3* |
| 82 | 11 | 18400001 | 18700000 | 0.500 | 2 | *NF1, ENSOARG00000026354* |
| 83 | 11 | 24400001 | 24900000 | 0.600 | 15 | *UBE2G1, SNORA62, SPNS3, SPNS2, MYBBP1A, GGT6, TEKT1, SMTNL2, FBXO39, XAF1, SLC13A5, ENSOARG00000001178, MED31, TXNDC17, KIAA0753* |
| 84 | 12 | 69500001 | 70100000 | 0.400 | 6 | *DTL, INTS7, LPGAT1, ENSOARG00000022944, NEK2, ENSOARG00000004286* |
| 85 | 12 | 72100001 | 72500000 | 0.400 | 2 | *SLC30A1, ENSOARG00000025466* |
| 86 | 12 | 61200001 | 61600000 | 0.300 | 2 | *GLUL, TEDDM1* |
| 87 | 12 | 74000001 | 74300000 | 0.300 | 6 | *ENSOARG00000014055, ENSOARG00000014196, F13B, ENSOARG00000014405, ASPM, ZBTB41* |
| 88 | 13 | 19300001 | 19600000 | 0.400 | 2 | *ENSOARG00000017733, ARL5B* |
| 89 | 13 | 46300001 | 46700000 | 0.400 | 6 | *RASSF2, SLC23A2, TMEM230, PCNA, CDS2, ENSOARG00000022618* |
| 90 | 13 | 52800001 | 53200000 | 0.300 | 13 | *ENSOARG00000007634, ENSOARG00000007639, GINS1, ENSOARG00000007835, MYT1, NPBWR2, OPRL1, ENSOARG00000008219, RGS19, TCEA2, PRPF6, U6, ENSOARG00000009022* |
| 91 | 13 | 53600001 | 53900000 | 0.500 | 16 | *ARFGAP1, ENSOARG00000011497, ENSOARG00000011555, ENSOARG00000011588, YTHDF1, ENSOARG00000022609, SLC17A9, GID8, DIDO1, ENSOARG00000026241, TCFL5, ENSOARG00000012168, ENSOARG00000026242, ENSOARG00000026243, ENSOARG00000012415, MRGBP* |
| 92 | 13 | 55900001 | 56400000 | 0.300 | 3 | *FAM217B, PPP1R3D, SYCP2* |
| 93 | 13 | 56400001 | 56700000 | 0.300 | 8 | *PHACTR3, ENSOARG00000021945, ENSOARG00000015902, ZNF831, PRELID3B, ATP5F1E, TUBB1, CTSZ* |
| 94 | 13 | 57000001 | 57300000 | 0.300 | 4 | *STX16, ENSOARG00000024312, APCDD1L, VAPB* |
| 95 | 13 | 58700001 | 59000000 | 0.300 | 3 | *ZNF438, ENSOARG00000026216, SVIL* |
| 96 | 13 | 33400001 | 33700000 | 0.300 | 5 | *SLC23A2, TMEM230, PCNA, CDS2, ENSOARG00000022618* |
| 97 | 13 | 46400001 | 46700000 | 0.600 | 12 | *ENSOARG00000018131, RTF2, GCNT7, CASS4, 5S_rRNA, CSTF1, AURKA, FAM210B, MC3R, ENSOARG00000018467, SIRPB2, NSFL1C* |
| 98 | 13 | 52900001 | 53500000 | 0.300 | 30 | *GINS1, ENSOARG00000007835, MYT1, NPBWR2, OPRL1, ENSOARG00000008219, RGS19, TCEA2, PRPF6, U6, ENSOARG00000009022, ZNF512B, UCKL1, ENSOARG00000022250, DNAJC5, TPD52L2, ABHD16B, ZBTB46, ENSOARG00000026240, ZGPAT, ARFRP1, ENSOARG00000010322, ENSOARG00000010368, STMN3, GMEB2, FNDC11, SRMS, PTK6, ENSOARG00000010995, EEF1A2* |
| 99 | 13 | 61000001 | 61300000 | 0.400 | 5 | *PLAGL2, POFUT1, KIF3B, ASXL1, NOL4L* |
| 100 | 14 | 12400001 | 12800000 | 0.300 | 8 | *ENSOARG00000011684, FBXO31, ENSOARG00000011861, ZCCHC14, JPH3, ENSOARG00000021615, KLHDC4, ENSOARG00000012077* |
| 101 | 14 | 38400001 | 38700000 | 0.300 | 14 | *LRRC36, TPPP3, ZDHHC1, HSD11B2, ATP6V0D1, AGRP, RIPOR1, CTCF, CARMIL2, ACD, PARD6A, ENKD1, C16orf86, GFOD2* |
| 102 | 14 | 34300001 | 34600000 | 0.300 | 9 | *PKD1L3, IST1, U6, ZNF821, ATXN1L, AP1G1, ENSOARG00000022908, PHLPP2, U6* |
| 103 | 15 | 59000001 | 59300000 | 0.300 | 3 | *ENSOARG00000015419, FSHB, ARL14EP* |
| 104 | 15 | 64600001 | 64900000 | 0.300 | 2 | *KIAA1549L, ENSOARG00000006878* |
| 105 | 15 | 62200001 | 62500000 | 0.300 | 1 | *ENSOARG00000019022* |
| 106 | 17 | 62000001 | 62300000 | 0.400 | 15 | *ENSOARG00000025686, BICDL1, ENSOARG00000010963, RAB35, GCN1, ENSOARG00000011805, ENSOARG00000025687, PXN, ENSOARG00000025688, ENSOARG00000011982, U4, SIRT4, U4, PLA2G1B, MSI1* |
| 107 | 18 | 38700001 | 39100000 | 0.300 | 3 | *ENSOARG00000024961, ENSOARG00000026446, PRKD1* |
| 108 | 18 | 40800001 | 41100000 | 0.400 | 4 | *HEATR5A, DTD2, GPR33, NUBPL* |
| 109 | 19 | 30900001 | 31300000 | 0.300 | 0 | *_* |
| 110 | 19 | 31400001 | 31700000 | 0.500 | 2 | *ENSOARG00000009783, MITF* |
| 111 | 19 | 37200001 | 37700000 | 0.300 | 6 | *PRICKLE2, PSMD6, ATXN7, THOC7, C3orf49, ENSOARG00000011488* |
| 112 | 19 | 47500001 | 47800000 | 0.300 | 5 | *CACNA1D, DCP1A, TKT, PRKCD, ENSOARG00000000381* |
| 113 | 19 | 48100001 | 48400000 | 0.400 | 14 | *NEK4, ENSOARG00000024525, SPCS1, GLT8D1, GNL3, ENSOARG00000023198, ENSOARG00000023719, ENSOARG00000023939, ENSOARG00000023714, PBRM1, SMIM4, NT5DC2, ENSOARG00000003517, NISCH* |
| 114 | 19 | 29100001 | 29500000 | 0.300 | 1 | *RYBP* |
| 115 | 19 | 47900001 | 48200000 | 0.400 | 16 | *SFMBT1, ENSOARG00000000541, ENSOARG00000000606, ITIH4, ITIH3, ITIH1, NEK4, ENSOARG00000024525, SPCS1, GLT8D1, GNL3, ENSOARG00000023198, ENSOARG00000023719, ENSOARG00000023939, ENSOARG00000023714, PBRM1* |
| 116 | 20 | 16600001 | 17000000 | 0.300 | 1 | *ENSOARG00000005969* |
| 117 | 20 | 3600001 | 3900000 | 0.300 | 22 | *ENSOARG00000004164, PTCRA, CNPY3, GNMT, PEX6, PPP2R5D, ENSOARG00000004853, KLHDC3, ENSOARG00000005094, MRPL2, KLC4, PTK7, SRF, ENSOARG00000023073, CUL9, ENSOARG00000006223, DNPH1, TTBK1, ENSOARG00000026857, SLC22A7, CRIP3, U6* |
| 118 | 23 | 25100001 | 25400000 | 0.300 | 4 | *GAREM1, ENSOARG00000024030, MEP1B, RNF138* |
| 119 | 25 | 39000001 | 39300000 | 0.300 | 2 | *CCSER2, ENSOARG00000000408* |
| 120 | 26 | 14900001 | 15200000 | 0.300 | 7 | *TLR3, FAM149A, CYP4V2, KLKB1, F11, MTNR1A, FAT1* |

**Supplementary Tables S8.** The candidate regions spanning genes within the Sudanese sheep (SD_G1 vs SD_G2) sheep group identified *via* F_ST_.

| **Reg.** | **Chr.** | **Start** | **Stop** | **Size (Mb)** | **No of**  **Genes** | **Genes** |
| --- | --- | --- | --- | --- | --- | --- |
| 1 | 1 | 25000001 | 25300000 | 0.300 | 2 | *FAF1, CDKN2C* |
| 2 | 1 | 25500001 | 25800000 | 0.300 | 4 | *TTC39A, EPS15, U6, ENSOARG00000004620* |
| 3 | 1 | 69000001 | 69200000 | 0.200 | 1 | *EVI5* |
| 4 | 1 | 135200001 | 135400000 | 0.200 | 0 | *_* |
| 5 | 1 | 181400001 | 181600000 | 0.200 | 1 | *ENSOARG00000025592* |
| 6 | 1 | 252600001 | 252800000 | 0.200 | 4 | *EPHB1, U6, ENSOARG00000025633, KY* |
| 7 | 2 | 13900001 | 14100000 | 0.200 | 2 | *ENSOARG00000023905, EPB41L4B* |
| 8 | 2 | 104100001 | 104400000 | 0.300 | 6 | *FAM167A, U6, BLK, GATA4, NEIL2, ENSOARG00000015250* |
| 9 | 2 | 160200001 | 160500000 | 0.300 | 4 | *ENSOARG00000025821, ORC4, U2, ACVR2A* |
| 10 | 2 | 184000001 | 184200000 | 0.200 | 1 | *PTPN4* |
| 11 | 2 | 184300001 | 184500000 | 0.200 | 4 | *EPB41L5, U4, TMEM185B, RALB* |
| 12 | 2 | 192000001 | 192300000 | 0.300 | 2 | *GLS, STAT1* |
| 13 | 3 | 92500001 | 92800000 | 0.300 | 5 | *TGFA, ENSOARG00000025999, ADD2, ENSOARG00000016474, ENSOARG00000011205* |
| 14 | 3 | 95900001 | 96100000 | 0.200 | 4 | *BOLA3, MOB1A, ENSOARG00000012350, SLC4A5* |
| 15 | 3 | 107100001 | 107500000 | 0.400 | 4 | *TSPAN8, ENSOARG00000026011, LGR5, U6* |
| 16 | 3 | 119600001 | 120000000 | 0.400 | 1 | *ENSOARG00000024607* |
| 17 | 3 | 165700001 | 166000000 | 0.300 | 2 | *ENSOARG00000012542, ENSOARG00000012678* |
| 18 | 3 | 175000001 | 175200000 | 0.200 | 4 | *RFX4, ENSOARG00000018456, ENSOARG00000018473, RIC8B* |
| 19 | 3 | 175700001 | 175900000 | 0.200 | 1 | *BTBD11* |
| 20 | 3 | 192800001 | 193100000 | 0.300 | 4 | *ST8SIA1, ENSOARG00000020181, CMAS, ABCC9* |
| 21 | 3 | 205600001 | 205800000 | 0.200 | 6 | *ENSOARG00000001186, ENSOARG00000001334, ENSOARG00000020333, ENSOARG00000001353, M6PR, PHC1* |
| 22 | 4 | 87300001 | 87600000 | 0.300 | 2 | *TAS2R16, SLC13A1* |
| 23 | 5 | 12600001 | 12800000 | 0.200 | 9 | *U6, CDC37, PDE4A, KEAP1, ATG4D, KRI1, CDKN2D, AP1M2, SLC44A2* |
| 24 | 5 | 17800001 | 18000000 | 0.200 | 11 | *PIP5K1C, ENSOARG00000025296, CACTIN, TBXA2R, ENSOARG00000012310, HMG20B, MFSD12, ENSOARG00000012488, ENSOARG00000012501, DOHH, ENSOARG00000012622* |
| 25 | 5 | 28700001 | 28900000 | 0.200 | 1 | *SNCAIP* |
| 26 | 5 | 66900001 | 67200000 | 0.300 | 0 | *_* |
| 27 | 5 | 107300001 | 108000000 | 0.700 | 8 | *ENSOARG00000010481, WDR36, ENSOARG00000000146, ENSOARG00000010495, ENSOARG00000025339, CAMK4, STARD4, ENSOARG00000025340* |
| 28 | 6 | 27800001 | 28000000 | 0.200 | 0 | *_* |
| 29 | 6 | 31700001 | 32200000 | 0.500 | 2 | *ENSOARG00000000376, ENSOARG00000018295* |
| 30 | 6 | 69700001 | 70100000 | 0.400 | 2 | *PDGFRA, ENSOARG00000021645* |
| 31 | 6 | 73500001 | 73700000 | 0.200 | 0 | *_* |
| 32 | 6 | 78000001 | 78200000 | 0.200 |  | *_* |
| 33 | 6 | 83100001 | 83300000 | 0.200 | 3 | *CENPC, STAP1, UBA6* |
| 34 | 6 | 85500001 | 85800000 | 0.300 | 8 | *UTP3, ENSOARG00000008596, ENSOARG00000011228, AMTN, AMBN, ENAM, JCHAIN, RUFY3* |
| 35 | 6 | 115700001 | 116100000 | 0.400 | 2 | *ENSOARG00000015218, ENSOARG00000015260* |
| 36 | 7 | 85000001 | 85200000 | 0.200 | 6 | *VASH1, ANGEL1, LRRC74A, 5S_rRNA, ENSOARG00000026721, IRF2BPL* |
| 37 | 7 | 89200001 | 89500000 | 0.300 | 3 | *CEP128, TSHR, GTF2A1* |
| 38 | 8 | 57600001 | 57800000 | 0.200 | 3 | *STX7, U6, TAAR9* |
| 39 | 8 | 62000001 | 62300000 | 0.300 | 3 | *IL20RA, IL22RA2, IFNGR1* |
| 40 | 8 | 71500001 | 71700000 | 0.200 | 2 | *STXBP5, SAMD5* |
| 41 | 8 | 90300001 | 90800000 | 0.500 | 8 | *ENSOARG00000027055, ENSOARG00000005108, ENSOARG00000027056, ENSOARG00000027057, FAM120B, PSMB1, ENSOARG00000005142, PDCD2* |
| 42 | 9 | 49600001 | 49800000 | 0.200 | 2 | *STAU2, ENSOARG00000024274* |
| 43 | 10 | 26400001 | 26800000 | 0.400 | 1 | *NBEA* |
| 44 | 10 | 46100001 | 46300000 | 0.200 | 0 | *_* |
| 45 | 10 | 50200001 | 50400000 | 0.200 | 0 | *_* |
| 46 | 10 | 71800001 | 72100000 | 0.300 | 5 | *ENSOARG00000001553, ENSOARG00000001594, ENSOARG00000001672, ENSOARG00000001678, ENSOARG00000001808* |
| 47 | 10 | 78100001 | 78400000 | 0.300 | 6 | *TPP2, METTL21C, ENSOARG00000005160, TEX30, POGLUT2, ENSOARG00000005295* |
| 48 | 11 | 14200001 | 14400000 | 0.200 | 8 | *MMP28, C17orf50, GAS2L2, RASL10B, AP2B1, PEX12, ENSOARG00000024827, SLFN14* |
| 49 | 11 | 26000001 | 26200000 | 0.200 | 17 | *KIF1C, INCA1, CAMTA2, SPAG7, ENO3, RNF167, ENSOARG00000005695, SLC25A11, ENSOARG00000005796, ENSOARG00000004290, CHRNE, C17orf107, MINK1, PLD2, GLTPD2, VMO1, TM4SF5* |
| 50 | 11 | 43800001 | 44000000 | 0.200 | 10 | *ENSOARG00000000126, MEIOC, CCDC43, DBF4B, ADAM11, GJC1, U6, HIGD1B, EFTUD2, ENSOARG00000023436* |
| 51 | 12 | 34500001 | 34800000 | 0.300 | 1 | *ENSOARG00000009327* |
| 52 | 12 | 69500001 | 69700000 | 0.200 | 2 | *DTL, INTS7* |
| 53 | 13 | 27500001 | 27700000 | 0.200 | 1 | *FRMD4A* |
| 54 | 13 | 46400001 | 46600000 | 0.200 | 1 | *SLC23A2* |
| 55 | 13 | 56200001 | 56600000 | 0.400 | 3 | *PHACTR3, ENSOARG00000015902, ZNF831* |
| 56 | 13 | 62100001 | 62300000 | 0.200 | 7 | *ENSOARG00000006525, BPIFA3, BPIFA1, BPIFB1, ENSOARG00000007192, ENSOARG00000007423, CDK5RAP1* |
| 57 | 14 | 49500001 | 49700000 | 0.200 | 11 | *ERICH4, CYP2B6, ENSOARG00000007316, DMAC2, B3GNT8, BCKDHA, EXOSC5, ENSOARG00000007421, B9D2, TGFB1, CCDC97* |
| 58 | 16 | 35400001 | 35600000 | 0.200 | 3 | *RICTOR, OSMR, ENSOARG00000005699* |
| 59 | 17 | 34200001 | 34600000 | 0.400 | 3 | *FGF2, ENSOARG00000023095, NUDT6, U4* |
| 60 | 17 | 71200001 | 71400000 | 0.200 | 15 | *TOP3B, PPM1F, ENSOARG00000025710, U6, MAPK1, YPEL1, PPIL2, ENSOARG00000025711, ENSOARG00000015779, ENSOARG00000022814, SDF2L1, ENSOARG00000015863, YDJC, ENSOARG00000016007, ENSOARG00000025712* |
| 61 | 18 | 23400001 | 23700000 | 0.300 | 5 | *ENSOARG00000014330, SAXO2, EFL1, MEX3B, ENSOARG00000023213* |
| 62 | 18 | 41300001 | 41600000 | 0.300 | 4 | *ENSOARG00000006406, ARHGAP5, U6, Vault* |
| 63 | 19 | 31400001 | 31800000 | 0.400 | 2 | *ENSOARG00000009783, MITF* |
| 64 | 19 | 37200001 | 37400000 | 0.200 | 1 | *PRICKLE2* |
| 65 | 19 | 47900001 | 48200000 | 0.300 | 16 | *SFMBT1, ENSOARG00000000541, ENSOARG00000000606, ITIH4, ITIH3, ITIH1, NEK4, ENSOARG00000024525, SPCS1, GLT8D1, GNL3, ENSOARG00000023198, ENSOARG00000023719, ENSOARG00000023939, ENSOARG00000023714, PBRM1* |
| 66 | 19 | 51400001 | 51600000 | 0.200 | 12 | *PLXNB1, SPINK8, NME6, ENSOARG00000001285, ENSOARG00000001392, ENSOARG00000001481, ENSOARG00000001605, ENSOARG00000026661, CATHL3, BAC5, ENSOARG00000002027, SC5* |
| 67 | 20 | 16700001 | 16900000 | 0.200 | 11 | *PPP2R5D, ENSOARG00000004853, KLHDC3, ENSOARG00000005094, MRPL2, KLC4, PTK7, SRF, ENSOARG00000023073, CUL9, ENSOARG00000006223* |
| 68 | 20 | 21200001 | 21500000 | 0.300 | 0 | *_* |
| 69 | 20 | 48500001 | 48800000 | 0.300 | 2 | *RPP40, CDYL* |
| 70 | 21 | 600001 | 900000 | 0.300 | 4 | *CEP295, ENSOARG00000026088, SMCO4, DEUP1* |
| 71 | 21 | 19600001 | 19800000 | 0.200 | 5 | *ENSOARG00000007473, 7SK, SNORD15* |
| 72 | 21 | 46500001 | 46700000 | 0.200 |  | *ANO1, FADD* |
| 73 | 23 | 43700001 | 44000000 | 0.300 | 5 | *LDLRAD4, FAM210A, RNMT, MC5R, MC2R* |

**Supplementary Table S9** The candidate regions spanning genes within the SD_G1 (*SD_G1 vs CN_G1* and *CN_G2*) sheep group identified *via* *F_ST_*.

| **Reg.** | **Chr.** | **Start** | **Stop** | **Size (Mb)** | **No of**  **Genes** | **Genes** |
| --- | --- | --- | --- | --- | --- | --- |
| 1 | 1 | 79700001 | 79900000 | 0.200 | 0 | _ |
| 2 | 1 | 105500001 | 105700000 | 0.200 | 3 | *ENSOARG00000022142, ETV3, ENSOARG00000006718* |
| 3 | 1 | 106000001 | 106200000 | 0.200 | 2 | *ENSOARG00000006768, ENSOARG00000006782* |
| 4 | 1 | 239200001 | 239400000 | 0.200 | 0 | *_* |
| 5 | 1 | 68700001 | 68900000 | 0.200 | 3 | *C1orf146, GLMN, RPAP2* |
| 6 | 1 | 118400001 | 118600000 | 0.200 | 2 | *GPR161, TIPRL* |
| 7 | 1 | 129000001 | 129200000 | 0.200 | 3 | *MRPL39, ENSOARG00000022750, U6* |
| 8 | 1 | 177100001 | 177300000 | 0.200 | 4 | *QTRT2, DRD3, U6, TIGIT* |
| 9 | 2 | 73200001 | 73400000 | 0.200 | 2 | *RIC1, ERMP1* |
| 10 | 2 | 122200001 | 123000000 | 0.800 | 3 | *ENSOARG00000016633, FSIP2, ENSOARG00000025803* |
| 11 | 2 | 160000001 | 160200000 | 0.200 | 1 | *ENSOARG00000025821* |
| 12 | 2 | 104600001 | 104800000 | 0.200 | 3 | *ENSOARG00000009177, ENSOARG00000015277, ADAM29* |
| 13 | 2 | 219700001 | 220000000 | 0.300 | 10 | *CYP27A1, PRKAG3, WNT6, WNT10A, ENSOARG00000025849, CDK5R2, FEV, CRYBA2, CFAP65, IHH* |
| 14 | 3 | 153700001 | 153900000 | 0.200 | 1 | *ENSOARG00000002929* |
| 15 | 3 | 185700001 | 185900000 | 0.200 | 0 | *_* |
| 16 | 3 | 213400001 | 213600000 | 0.200 | 9 | *CDC42EP1, LGALS2, GGA1, ENSOARG00000013931, PDXP, LGALS1, ENSOARG00000013969, TRIOBP, ENSOARG00000023973* |
| 17 | 3 | 132100001 | 132400000 | 0.300 | 12 | *NFE2, ENSOARG00000016240, CBX5, ENSOARG00000016261, HOXC4, HOXC5, HOXC6, HOXC8, HOXC9, HOXC10, HOXC11, ENSOARG00000026026* |
| 18 | 3 | 174600001 | 174800000 | 0.200 | 5 | *NUAK1, CKAP4, TCP11L2, POLR3B, ENSOARG00000026040* |
| 19 | 4 | 10200001 | 10400000 | 0.200 | 3 | *U6, SAMD9, HEPACAM2* |
| 20 | 4 | 29600001 | 29800000 | 0.200 | 0 | *_* |
| 21 | 4 | 94100001 | 94300000 | 0.200 | 9 | *CPA5, CPA1, CEP41, ENSOARG00000022095, ENSOARG00000021327, ENSOARG00000022560, MEST, ENSOARG00000024045, COPG2* |
| 22 | 4 | 101600001 | 101800000 | 0.200 | 0 | *_* |
| 23 | 5 | 47500001 | 47700000 | 0.200 | 4 | *5S_rRNA, LRRTM2, CTNNA1, SIL1* |
| 24 | 5 | 107400001 | 108000000 | 0.600 | 7 | *WDR36, ENSOARG00000010495, ENSOARG00000025339, CAMK4, STARD4, ENSOARG00000025340, ENSOARG00000000146* |
| 25 | 5 | 78000001 | 78200000 | 0.200 | 3 | *ANKRD34B, ENSOARG00000014803, MSH3* |
| 26 | 6 | 69700001 | 70000000 | 0.300 | 0 | *PDGFRA, ENSOARG00000021645* |
| 27 | 6 | 116400001 | 116700000 | 0.300 | 10 | *ENSOARG00000016183, ENSOARG00000016260, ENSOARG00000016268, ENSOARG00000016282, GAK, CPLX1, ENSOARG00000016536, ENSOARG00000026630, ENSOARG00000026631, ENSOARG00000026632* |
| 28 | 6 | 116800001 | 117100000 | 0.300 | 12 | *ENSOARG00000008606, MAEA, ENSOARG00000016735, ENSOARG00000016756, SLC49A3, PDE6B, PIGG, ENSOARG00000026634, ENSOARG00000026635, ENSOARG00000026636, ENSOARG00000026637, ENSOARG00000026638* |
| 29 | 7 | 22500001 | 22700000 | 0.200 | 21 | *ENSOARG00000019537, ENSOARG00000019538, TRAV16, ENSOARG00000019543, ENSOARG00000019546, ENSOARG00000019550, TRAV24, ENSOARG00000019555, ENSOARG00000019558, ENSOARG00000019560, ENSOARG00000019562, ENSOARG00000012009, ENSOARG00000019567, TRAV21, ENSOARG00000019571, ENSOARG00000019573, ENSOARG00000019576, ENSOARG00000019580, ENSOARG00000019582, ENSOARG00000019586, ENSOARG00000019589* |
| 30 | 7 | 49000001 | 49200000 | 0.200 | 1 | *AQP9* |
| 31 | 7 | 66900001 | 67100000 | 0.200 | 2 | *SLC35F4, 7SK* |
| 32 | 7 | 79000001 | 79200000 | 0.200 | 4 | *ENSOARG00000001056, ADAM21, ADAM20, MED6* |
| 33 | 9 | 67500001 | 67700000 | 0.200 | 2 | *EBAG9, PKHD1L1* |
| 34 | 10 | 35800001 | 36000000 | 0.200 | 5 | *SAP18, ENSOARG00000026299, ENSOARG00000026300, LATS2, XPO4* |
| 35 | 10 | 36100001 | 36500000 | 0.400 | 10 | *IFT88, CRYL1, GJB6, ENSOARG00000026301, GJB2, GJA3, ZMYM2, ENSOARG00000014110, ENSOARG00000014118, PSPC1* |
| 36 | 10 | 70700001 | 71000000 | 0.300 | 5 | *ENSOARG00000001114, ENSOARG00000001149, ENSOARG00000001156, ENSOARG00000001163, ENSOARG00000001169* |
| 37 | 10 | 71000001 | 71400000 | 0.400 | 4 | *ENSOARG00000001169, ENSOARG00000001221, ENSOARG00000001232, ENSOARG00000001282* |
| 38 | 10 | 71900001 | 72100000 | 0.200 | 2 | *ENSOARG00000001594, ENSOARG00000001808* |
| 39 | 11 | 100000 | 300000 | 0.200 | 2 | *ENSOARG00000003921, ENSOARG00000004061* |
| 40 | 11 | 10200001 | 10400000 | 0.200 | 6 | *CLTC, PTRH2, VMP1, oar-mir-21, TUBD1, RPS6KB1* |
| 41 | 11 | 24600001 | 24800000 | 0.200 | 7 | *SPNS2, MYBBP1A, GGT6, TEKT1, SMTNL2, FBXO39, XAF1* |
| 42 | 13 | 42600001 | 42800000 | 0.200 | 5 | *ENSOARG00000010577, ENSOARG00000010661, ENSOARG00000010907, ENSOARG00000011087, ENSOARG00000011490* |
| 43 | 13 | 56200001 | 56500000 | 0.300 | 3 | *PHACTR3, ENSOARG00000015902, ZNF831* |
| 44 | 13 | 42500001 | 42800000 | 0.300 | 9 | *ENSOARG00000010221, ENSOARG00000010355, ENSOARG00000010449, ENSOARG00000010488, ENSOARG00000010577, ENSOARG00000010661, ENSOARG00000010907, ENSOARG00000011087, ENSOARG00000011490,* |
| 45 | 14 | 34400001 | 34600000 | 0.200 | 10 | *ATP6V0D1, AGRP, RIPOR1, CTCF, CARMIL2, ACD, PARD6A, ENKD1, C16orf86, GFOD2* |
| 46 | 15 | 42400001 | 42600000 | 0.200 | 2 | *SBF2, SWAP70* |
| 47 | 16 | 31600001 | 31800000 | 0.200 | 3 | *ENSOARG00000005684, ENSOARG00000008775, CCDC152* |
| 48 | 16 | 70600001 | 70800000 | 0.200 | 7 | *ENSOARG00000026989, ENSOARG00000015729, ENSOARG00000015756, EXOC3, ENSOARG00000026990, SLC9A3, CEP72* |
| 49 | 17 | 53600001 | 53900000 | 0.300 | 4 | *P2RX7, IFT81, ATP2A2, ANAPC7* |
| 50 | 18 | 400001 | 600000 | 0.200 | 0 | *_* |
| 51 | 18 | 14300001 | 14500000 | 0.200 | 0 | *_* |
| 52 | 18 | 23300001 | 23500000 | 0.200 | 3 | *ENSOARG00000014330, SAXO2, EFL1* |
| 53 | 18 | 32200001 | 32400000 | 0.200 | 6 | *PTPN9, SIN3A, MAN2C1, NEIL1, ENSOARG00000021972, COMMD4* |
| 54 | 19 | 3300001 | 3500000 | 0.200 | 0 | *_* |
| 55 | 19 | 29600001 | 29800000 | 0.200 | 0 | *_* |
| 56 | 19 | 38100001 | 38300000 | 0.200 | 1 | *SYNPR* |
| 57 | 20 | 33800001 | 34100000 | 0.300 | 7 | *ENSOARG00000008416, U6, ENSOARG00000026877, ENSOARG00000008492, ENSOARG00000008523, UBE2N, ENSOARG00000008627* |
| 58 | 20 | 33700001 | 34000000 | 0.300 | 7 | *ENSOARG00000008381, ENSOARG00000008383, U6, ENSOARG00000008416, ENSOARG00000026877, ENSOARG00000008492, ENSOARG00000008523* |
| 59 | 21 | 36900001 | 37200000 | 0.300 | 7 | *ENSOARG00000001096, ENSOARG00000001484, ENSOARG00000001993, ENSOARG00000002825, ENSOARG00000003195, ENSOARG00000003295, ENSOARG00000003462* |
| 60 | 21 | 38500001 | 38800000 | 0.300 | 7 | *ENSOARG00000026107, ENSOARG00000009227, ENSOARG00000009820, ENSOARG00000010340, ENSOARG00000010613, ENSOARG00000011070, PAG6* |
| 61 | 21 | 49600001 | 49800000 | 0.200 | 12 | *ENSOARG00000005947, SIGIRR, ANO9, ENSOARG00000026140, PKP3, B4GALNT4, ENSOARG00000008115, ENSOARG00000008178, ENSOARG00000008211, ENSOARG00000015728, IFITM5, PGGHG* |
| 62 | 23 | 60800001 | 61000000 | 0.200 | 4 | *PIGN, RELCH, ENSOARG00000006094, SNORD36* |
| 63 | 23 | 100000 | 200000 | 0.100 | 3 | *ENSOARG00000003981, ENSOARG00000003748, ENSOARG00000003987* |
| 64 | 24 | 10400001 | 10600000 | 0.200 | 5 | *TXNDC11, ZC3H7A, ENSOARG00000010696, RSL1D1, GSPT1* |
| 65 | 25 | 21400001 | 21600000 | 0.200 | 0 | *_* |

**Supplementary Table S10** The candidate regions spanning genes within the SD_G2 (*SD_G2 vs CN_G1* and *CN_G2*) sheep group identified *via* *F_ST_*.

| **Reg.** | **Chr.** | **Start** | **Stop** | **Size (Mb)** | **No of**  **Genes** | **Genes** |
| --- | --- | --- | --- | --- | --- | --- |
| 1 | 1 | 20400001 | 20600000 | 0.200 | 5 | *ENSOARG00000002180, U6, TSPAN1, POMGNT1, LURAP1* |
| 2 | 1 | 24900001 | 25100000 | 0.200 | 1 | *FAF1* |
| 3 | 1 | 105500001 | 105700000 | 0.200 | 3 | *ENSOARG00000022142, ETV3, ENSOARG00000006718* |
| 4 | 1 | 129000001 | 129200000 | 0.200 | 3 | *MRPL39, ENSOARG00000022750, U6* |
| 5 | 1 | 199600001 | 199800000 | 0.200 | 2 | *IGF2BP2, SENP2* |
| 6 | 1 | 255100001 | 255300000 | 0.200 | 4 | *ACAD11, ACKR4, DNAJC13, ENSOARG00000009070* |
| 7 | 1 | 100100001 | 100300000 | 0.200 | 6 | *ZNF687, PI4KB, RFX5, SELENBP1, PSMB4, POGZ* |
| 8 | 1 | 114900001 | 115100000 | 0.200 | 1 | *ENSOARG00000011294* |
| 9 | 1 | 117900001 | 118100000 | 0.200 | 4 | *CREG1, ENSOARG00000012185, MPZL1, ADCY10* |
| 10 | 1 | 128900001 | 129200000 | 0.300 | 4 | *JAM2, MRPL39, ENSOARG00000022750, U6* |
| 11 | 2 | 122200001 | 122600000 | 0.400 | 2 | *ENSOARG00000016633, FSIP2* |
| 12 | 2 | 184000001 | 184500000 | 0.500 | 5 | *PTPN4, EPB41L5, U4, TMEM185B, RALB* |
| 13 | 2 | 111700001 | 111900000 | 0.200 | 5 | *ENSOARG00000009336, ENSOARG00000009365, 5S_rRNA, 5S_rRNA, ENSOARG00000015627* |
| 14 | 3 | 106100001 | 106400000 | 0.300 | 4 | *TMEM87B, FBLN7, ZC3H8, ZC3H6* |
| 15 | 3 | 106600001 | 106900000 | 0.300 | 2 | *ENSOARG00000016975, ENSOARG00000014533* |
| 16 | 3 | 107100001 | 107300000 | 0.200 | 1 | *TSPAN8* |
| 17 | 3 | 129700001 | 129900000 | 0.200 | 2 | *SOCS2, CRADD* |
| 18 | 3 | 153700001 | 154000000 | 0.300 | 2 | *ENSOARG00000002929, ENSOARG00000023603* |
| 19 | 3 | 163100001 | 163400000 | 0.300 | 18 | *MYL6, ENSOARG00000010153, ESYT1, ZC3H10, ENSOARG00000010335, ERBB3, IKZF4, SUOX, RAB5B, CDK2, PMEL, DGKA, PYM1, MMP19, ENSOARG00000011042, ENSOARG00000011120, ORMDL2, SARNP* |
| 20 | 3 | 213400001 | 213600000 | 0.200 | 9 | *CDC42EP1, LGALS2, GGA1, ENSOARG00000013931, PDXP, LGALS1, ENSOARG00000013969, TRIOBP, ENSOARG00000023973* |
| 21 | 3 | 400001 | 700000 | 0.300 | 16 | *ENSOARG00000000775, DPH7, ENSOARG00000018872, PNPLA7, NSMF, ENSOARG00000000961, ENSOARG00000025923, ENSOARG00000001058, ENSOARG00000025924, ENSOARG00000001078, ENSOARG00000001099, ENSOARG00000001120, FAM166A, ENSOARG00000025925, ENTPD8, NOXA1* |
| 22 | 3 | 129700001 | 129900000 | 0.200 | 2 | *SOCS2, CRADD* |
| 23 | 4 | 29600001 | 29800000 | 0.200 | 0 | *_* |
| 24 | 4 | 48500001 | 48800000 | 0.300 | 8 | *COG5, GPR22, DUS4L, BCAP29, ENSOARG00000024106, SLC26A4, CBLL1, SLC26A3* |
| 25 | 4 | 72500001 | 72700000 | 0.200 | 0 | *_* |
| 26 | 4 | 119100001 | 119400000 | 0.300 | 2 | *VIPR2, ENSOARG00000025276* |
| 27 | 4 | 48600001 | 48800000 | 0.200 | 6 | *DUS4L, BCAP29, ENSOARG00000024106, SLC26A4, CBLL1, SLC26A3* |
| 28 | 5 | 47500001 | 47800000 | 0.300 | 5 | *CTNNA1, 5S_rRNA, LRRTM2, SIL1, ENSOARG00000016879* |
| 29 | 5 | 51500001 | 51700000 | 0.200 | 1 | *ARHGAP26* |
| 30 | 6 | 116400001 | 116700000 | 0.300 | 10 | *ENSOARG00000016183, ENSOARG00000016260, ENSOARG00000016268, ENSOARG00000016282, GAK, CPLX1, ENSOARG00000016536, ENSOARG00000026630, ENSOARG00000026631, ENSOARG00000026632* |
| 31 | 6 | 116800001 | 117100000 | 0.300 | 12 | *ENSOARG00000008606, MAEA, ENSOARG00000016735, ENSOARG00000016756, SLC49A3, PDE6B, PIGG, ENSOARG00000026634, ENSOARG00000026635, ENSOARG00000026636, ENSOARG00000026637, ENSOARG00000026638* |
| 32 | 7 | 22500001 | 22700000 | 0.200 | 21 | *ENSOARG00000019537, ENSOARG00000019538, TRAV16, ENSOARG00000019543, ENSOARG00000019546, ENSOARG00000019550, TRAV24, ENSOARG00000019555, ENSOARG00000019558, ENSOARG00000019560, ENSOARG00000019562, ENSOARG00000012009, ENSOARG00000019567, TRAV21, ENSOARG00000019571, ENSOARG00000019573, ENSOARG00000019576, ENSOARG00000019580, ENSOARG00000019582, ENSOARG00000019586, ENSOARG00000019589* |
| 33 | 7 | 49000001 | 49300000 | 0.300 | 2 | *AQP9, ALDH1A2* |
| 34 | 8 | 90400001 | 90800000 | 0.400 | 8 | *ENSOARG00000005108, FAM120B, PSMB1, ENSOARG00000005142, PDCD2, ENSOARG00000027055, ENSOARG00000027056, ENSOARG00000027057* |
| 35 | 8 | 90500001 | 90800000 | 0.300 | 5 | *FAM120B, PSMB1, ENSOARG00000005142, PDCD2, ENSOARG00000027057* |
| 36 | 10 | 70800001 | 71000000 | 0.200 | 4 | *ENSOARG00000001149, ENSOARG00000001156, ENSOARG00000001163, ENSOARG00000001169* |
| 37 | 10 | 71200001 | 71400000 | 0.200 | 2 | *ENSOARG00000001232, ENSOARG00000001282* |
| 38 | 10 | 7300001 | 7600000 | 0.300 | 2 | *ENSOARG00000006632, ENSOARG00000006641* |
| 39 | 10 | 70600001 | 70900000 | 0.300 | 4 | *ENSOARG00000001114, ENSOARG00000001149, ENSOARG00000001156, ENSOARG00000001163* |
| 40 | 10 | 76600001 | 76800000 | 0.200 | 1 | *TMTC4* |
| 41 | 11 | 26200001 | 26400000 | 0.200 | 9 | *TM4SF5, ZMYND15, ENSOARG00000007683, MED11, ARRB2, PELP1, ALOX15, ENSOARG00000008647, ENSOARG00000008786* |
| 42 | 11 | 1 | 300000 | 0.300 | 2 | *ENSOARG00000003921, ENSOARG00000004061* |
| 43 | 11 | 51200001 | 51400000 | 0.200 | 4 | *NPTX1, ENDOV, RNF213, ENSOARG00000001403* |
| 44 | 13 | 46900001 | 47100000 | 0.200 | 2 | *U6, GPCPD1* |
| 45 | 13 | 42600001 | 42800000 | 0.200 | 5 | *ENSOARG00000010577, ENSOARG00000010661, ENSOARG00000010907, ENSOARG00000011087, ENSOARG00000011490* |
| 46 | 13 | 49700001 | 49900000 | 0.200 | 1 | *ENSOARG00000004705* |
| 47 | 13 | 62900001 | 63100000 | 0.200 | 5 | *RALY, EIF2S2, SNORA73, ENSOARG00000009308, ENSOARG00000009354* |
| 48 | 14 | 13300001 | 13500000 | 0.200 | 11 | *ENSOARG00000012499, ENSOARG00000012590, ZC3H18, IL17C, CYBA, MVD, ENSOARG00000013069, CTU2, RNF166, PIEZO1, ENSOARG00000024324* |
| 49 | 14 | 57200001 | 57500000 | 0.300 | 4 | *ENSOARG00000019898, ENSOARG00000000120, ENSOARG00000026938, ENSOARG00000000134* |
| 50 | 16 | 70500001 | 70800000 | 0.300 | 10 | *ENSOARG00000026989, ENSOARG00000015619, ENSOARG00000015678, ENSOARG00000015729, ENSOARG00000015756, 5S_rRNA, EXOC3, ENSOARG00000026990, SLC9A3, CEP72* |
| 51 | 17 | 52300001 | 52500000 | 0.200 | 6 | *CCDC62, DENR, SNORA70, HCAR1, ENSOARG00000004617, KNTC1* |
| 52 | 18 | 26300001 | 26500000 | 0.200 | 6 | *ENSOARG00000015918, MCEE, MPHOSPH10, FAN1, MTMR10, TRPM1* |
| 53 | 18 | 19200001 | 19400000 | 0.200 | 7 | *ENSOARG00000011097, ENSOARG00000017388, ENSOARG00000017401, ENSOARG00000017413, ENSOARG00000011119, ENSOARG00000011124, ENSOARG00000017428* |
| 54 | 18 | 23300001 | 23500000 | 0.200 | 3 | *ENSOARG00000014330, SAXO2, EFL1* |
| 55 | 18 | 32200001 | 32500000 | 0.300 | 10 | *PTPN9, SIN3A, MAN2C1, NEIL1, ENSOARG00000021972, COMMD4, ENSOARG00000002552, ENSOARG00000017499, ENSOARG00000002566, C15orf39* |
| 56 | 19 | 3300001 | 3500000 | 0.200 | 0 | *_* |
| 57 | 19 | 47900001 | 48200000 | 0.300 | 16 | *SFMBT1, ENSOARG00000000541, ENSOARG00000000606, ITIH4, ITIH3, ITIH1, NEK4, ENSOARG00000024525, SPCS1, GLT8D1, GNL3, ENSOARG00000023198, ENSOARG00000023719, ENSOARG00000023939, ENSOARG00000023714, PBRM1* |
| 58 | 19 | 34500001 | 34700000 | 0.200 | 2 | *SNORD22, ENSOARG00000024915* |
| 59 | 19 | 38100001 | 38300000 | 0.200 | 1 | *SYNPR* |
| 60 | 19 | 47900001 | 48100000 | 0.200 | 6 | *SFMBT1, ENSOARG00000000541, ENSOARG00000000606, ITIH4, ITIH3, ITIH1* |
| 61 | 20 | 33800001 | 34100000 | 0.300 | 7 | *ENSOARG00000008416, U6, ENSOARG00000026877, ENSOARG00000008492, ENSOARG00000008523, UBE2N, ENSOARG00000008627* |
| 62 | 20 | 33700001 | 34000000 | 0.300 | 7 | *ENSOARG00000008381, U6, ENSOARG00000008383, ENSOARG00000008416, ENSOARG00000026877, ENSOARG00000008492, ENSOARG00000008523* |
| 63 | 21 | 49500001 | 49700000 | 0.200 | 13 | *ENSOARG00000005947, ENSOARG00000026139, RASSF7, ENSOARG00000007058, ENSOARG00000007214, HRAS, ENSOARG00000007409, ENSOARG00000007453, SIGIRR, ANO9, ENSOARG00000026140, PKP3, B4GALNT4* |
| 64 | 21 | 36900001 | 37100000 | 0.200 | 5 | *ENSOARG00000001096, ENSOARG00000001484, ENSOARG00000001993, ENSOARG00000002825, ENSOARG00000003195* |
| 65 | 21 | 38500001 | 38800000 | 0.300 | 7 | *ENSOARG00000026107, ENSOARG00000009227, ENSOARG00000009820, ENSOARG00000010340, ENSOARG00000010613, ENSOARG00000011070, PAG6* |
| 66 | 21 | 49500001 | 49800000 | 0.300 | 19 | *ENSOARG00000005947, ENSOARG00000026139, RASSF7, ENSOARG00000007058, ENSOARG00000007214, HRAS, ENSOARG00000007409, ENSOARG00000007453, SIGIRR, ANO9, ENSOARG00000026140, PKP3, B4GALNT4, ENSOARG00000008115, ENSOARG00000008178, ENSOARG00000008211, ENSOARG00000015728, IFITM5, PGGHG* |
| 67 | 25 | 21400001 | 21700000 | 0.300 | 0 | *_* |
| 68 | 26 | 33000001 | 33300000 | 0.300 | 3 | *ENSOARG00000001975, ENSOARG00000002025, ADAM2* |
